# 3D-local noisy shallow quantum circuits defeat unbounded fan-in classical circuits

**DOI:** 10.1038/s41467-026-76560-x

**Published:** 2026-08-11

**Authors:** Libor Caha, Xavier Coiteux-Roy, Robert Koenig

**Affiliations:** 1 School of Computation, Information and Technology, Technical University of Munich, Munich, Germany; 2 Munich Center for Quantum Science and Technology, Munich, Germany; 3 School of Natural Sciences, Technical University of Munich, Munich, Germany; 4 Institute for Quantum Science and Technology, University of Calgary, Calgary, AB Canada

**Keywords:** Quantum information, Computer science

## Abstract

Although quantum computers are believed to be more powerful than classical ones, a convincing experimental demonstration of this fact remains elusive. Proposed schemes either rely on unproven complexity-theoretic hardness assumptions, and/or require universal, fault-tolerant scalable quantum computers to implement. This has motivated the study of restricted models of computation. Constant-depth quantum circuits are known to be more powerful than unbounded fan-in classical (${{\mathsf{AC}}}^{0}$-)circuits. Here we ask if this advantage persists in the presence of noise and under locality constraints. We present a computational problem for which every instance can be solved with near-certainty, despite noise, by a constant-depth quantum circuit with local operations in 3D. In contrast, every ${{\mathsf{AC}}}^{0}$-circuit of size smaller than a certain (sub)exponential fails with near-certainty on a uniformly random instance. This constitutes a proposal with built-in fault-tolerance to experimentally observe the strongest known complexity-theoretic separation between classical and quantum computation.

## Abstract (web summary)

Finding cases in which imperfect quantum information processing can surpass classical protocols would allow to make use of NISQ-era devices without having to wait for full fault-tolerance. Here, the authors describe a search problem that can be solved efficiently by 3D-local constant-depth noisy quantum circuits but not by constant-depth unbounded fan-in classical circuits.

## Introduction

In the present era of near- and intermediate-term quantum devices, fully fault-tolerant, universal, scalable quantum computers belong to the realm of science fiction. Fortunately, even with significantly more modest, imperfect resources, quantum information-processing can be superior to purely classical protocols. A key challenge is to identify such cases and characterize the potential of limited quantum devices. In the context of computation, this not only involves identifying computational problems that straddle the fine line between being amenable to quantum algorithmic solutions and being beyond the reach of comparable classical devices. It also requires designing computational architectures that can withstand noise and can realistically be built.

Circuit complexity provides a natural framework for these tasks. The study of circuit complexity has a long history in classical computer science, where the capabilities and limitations of different classes of circuits can be accurately characterized. Celebrated results include for example the statement that the parity of *n* bits cannot be computed in ${{\mathsf{AC}}}^{0}$, i.e., by polynomial-size, constant-depth circuits with unbounded fan-in AND, OR, and NOT gates^1–3^. It is also known, for example, that ${{\mathsf{AC}}}^{0}$ is richer than the class ${{\mathsf{NC}}}^{0}$ of problems solved by constant-depth bounded fan-in circuits. Trivially, a computational problem separating these two classes is that of computing the AND of *n* bits.

Motivated by the successes of classical circuit complexity, analogous quantum circuit classes have been investigated from the early days of quantum computing, see e.g., ref. ^4^. More recent work studied how quantum circuit complexity classes compare to classical ones. In ref. ^5^, it was shown that there is a relation problem which can be solved by a constant-depth (so-called shallow) quantum circuit, but whose solution by a classical circuit with bounded fan-in gates requires at least logarithmic depth. This provides an unconditional separation between ${{\mathsf{QNC}}}^{0}$ (the class of shallow quantum circuits) and ${{\mathsf{NC}}}^{0}$. A stronger complexity-theoretic result separating ${{\mathsf{QNC}}}^{0}$ from ${{\mathsf{AC}}}^{0}$ was subsequently established by Bene Watts et al.^6^: They showed that a certain problem, the so-called relaxed parity-halving problem, has the property of being solvable by a constant-depth quantum circuit, yet any classical ${{\mathsf{AC}}}^{0}$ circuit with unbounded fan-in gates requires a superpolynomial (in fact subexponential) size for this task.

While these results establish an advantage of certain shallow quantum circuits compared to similarly defined classical circuits (and, correspondingly, for the associated complexity classes), it is important to study whether or not these findings translate to an experimentally observable difference between quantum and classical devices in the real world. That is, is it possible to benefit from quantum information-processing when all operations and building blocks are noisy? Not surprisingly, this central question was also posed immediately after Shor’s discovery of the factoring algorithm and other early quantum algorithms. In that case, the (theoretical) resolution is the fault-tolerance threshold theorem ^7^: It ensures that quantum computations can be rendered fault-tolerant while incurring only a polynomial overhead in resources assuming the noise strength is below some threshold.

When studying shallow (i.e., constant-depth) quantum circuits, the standard constructions used to establish the fault-tolerance threshold theorem do not apply: This is because these typically do not preserve shallowness. This means that rendering complexity-theoretic separations fault-tolerant generally requires new, non-standard error-correction techniques. A first result in this direction was obtained in ref. ^8^, where it was shown that the separation between shallow quantum and bounded fan-in classical circuits (first demonstrated in ref. ^5^) can be made robust to noise: Even noisy shallow quantum circuits beat ideal classical ${{\mathsf{NC}}}^{0}$-circuits at a certain task. Here, we establish the strongest known complexity-theoretic separation between the computational power of noisy, 3*D*-local shallow quantum circuits as opposed to that of shallow classical circuits with unbounded fan-in gates.

## Results

We show that noisy shallow 3*D*-local quantum circuits solve a computational task with higher probability than (ideal) unbounded fan-in classical ${{\mathsf{AC}}}^{0}$-circuits of a certain subexponential size. This can thus be seen as a fault-tolerant counterpart to the work^6^. More precisely, we show the following (see Supplementary Note[Theorems 6.7 and 7.10]^9^):

### Theorem 1

(Fault-tolerant quantum advantage against ${{\mathsf{AC}}}^{0}$, informal version) Let (*μ*, *ν*) ∈ (0, 1)^2^ with *ν* < 1 − *μ* be arbitrary. There is a computational problem with the following properties:

(i) The problem is beyond the reach of ${{\mathsf{AC}}}^{0}$-circuits: Any ${{\mathsf{AC}}}^{0}$-circuit solving the problem with probability at least *ν* on average over a randomly chosen instance has superpolynomial size.
(ii) The problem can be solved with average probability at least 1 − *μ* by a 3*D*-local shallow quantum circuit even in the presence of local stochastic noise. That is, the quantum advantage can be observed using a shallow, noisy quantum circuit which only involves nearest-neighbor gates on qubits arranged on a regular 3*D* lattice.

Our result thus strengthens the findings of^8^: While requiring a comparable amount of (imperfect) quantum resources/capabilities, and only local operations in 3*D*, it establishes a quantum advantage against ${{\mathsf{AC}}}^{0}$ instead of only ${{\mathsf{NC}}}^{0}$. In addition, contrary to earlier work, our result features a classical–quantum gap (1 − *μ*) − *ν* (difference of success probabilities) arbitrarily close to 1, a fact we establish by using Raz’s parallel repetition result^10,11^ for one-round two-player games.

### From single-qubit gate teleportation to complexity theory

**Fig. 1:**
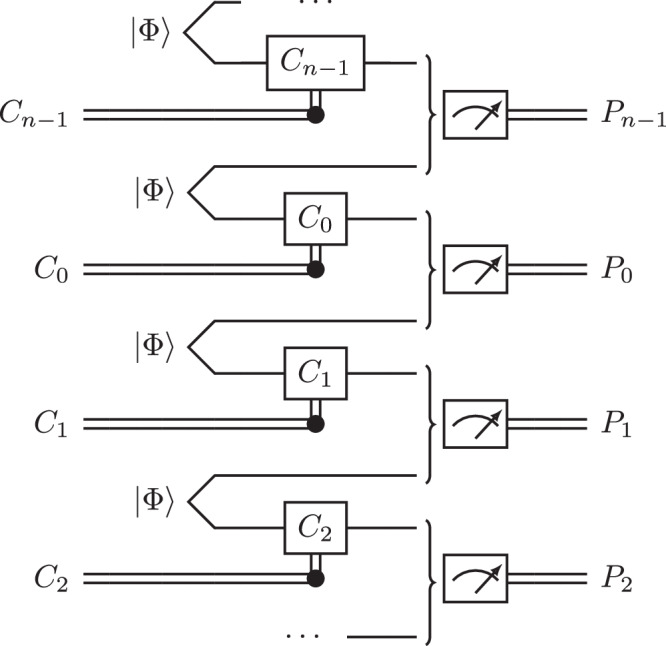
The gate-teleportation circuit. ${U}_{n}^{{\mathsf{Telep}}}$ is a classically controlled Clifford circuit. It takes as input *n* single-qubit Clifford group elements (*C*_0_, …, *C*_*n*−1_). Each of these is applied to half of a maximally entangled state $\left\vert \Phi \right\rangle={2}^{-1/2}(\left\vert 00\right\rangle+\left\vert 11\right\rangle )$ (i.e., this constitutes a classically controlled single-qubit Clifford gate.) If Bell measurements are performed on pairs of qubits (shifted by one), the output (*P*_0_, …, *P*_*n*−1_) is an *n*-tuple of Paulis.

Our result is obtained by identifying a particularly simple computational problem which is motivated by what we call the single-qubit gate-teleportation circuit (see Fig. 1 and ref. ^12^). This circuit is a concatenation of multiple applications of the standard gate-teleportation procedure^13^ for single-qubit Clifford gates, except for the fact that the final “Pauli correction" is not applied – we are only interested in the measurement outcomes produced in the repeated gate-teleportation procedure. In more detail, the single-qubit gate-teleportation circuit is a classically controlled Clifford circuit taking *n* single-qubit Clifford elements *C*_0_, …, *C*_*n*−1_ as input, and outputting the result of *n* Bell measurements, i.e., a sequence of *n* Pauli observables *P*_0_, …, *P*_*n*−1_ (corrections in gate-teleportation).

The computational problem we consider is the following: Given an input (*C*_0_, …, *C*_*n*−1_), output a sequence (*P*_0_, …, *P*_*n*−1_) of Paulis that occurs with nonzero probability in the output distribution of the gate-teleportation circuit. This can be formulated succinctly as follows: a correct output is one that satisfies

1 $${{\rm{tr}}}({P}_{n-1}{C}_{n-1}\cdots {P}_{0}{C}_{0})\,\ne \,0.$$

For more details see the Supplementary Note[Section 2]^9^.

On our route to establishing a quantum advantage of noisy shallow 3*D*-local quantum circuits against ${{\mathsf{AC}}}^{0}$, we show the following result for (ideal) 1*D*-local quantum circuits (see Supplementary Note[Corollary 5.4]^9^).

#### Theorem 2

(Single-qubit gate teleportation yields a quantum advantage against ${{\mathsf{AC}}}^{0}$, informal version) Let ${{\mathcal{C}}}$ be an ${{\mathsf{AC}}}^{0}$-circuit which, for a uniformly chosen sequence $C=({C}_{0},\ldots,{C}_{n-1})\in {{\mathsf{Cliff}}}^{n}$ of *n* single-qubit Clifford gates, produces – with probability at least 0.986 on average – a sequence $({P}_{0},\ldots,{P}_{n-1})\in {{\mathsf{Pauli}}}^{n}$ which occurs with non-zero probability in the output distribution of the single-qubit gate-teleportation circuit on input *C*. Then the size of ${{\mathcal{C}}}$ is superpolynomial (in fact subexponential). On the other hand, this problem is solved with certainty by a 1*D*-local ${{\mathsf{QNC}}}^{0}$ circuit.

This improves our result^12^ where we showed that the single-qubit gate-teleportation problem cannot be solved by an ${{\mathsf{NC}}}^{0}$ circuit.

Theorem 2 gives a version applicable for the proof of Theorem 6.7 with parameter *ν* = 0.986. In Section 7 of the Supplementary Note (Theorem 7.10), we show that this can be replaced by an arbitrary constant *ν* ∈ (0, 1) by considering a relation obtained by parallel repetition. This proof relies on a variant of Raz’s parallel repetition for nonlocal games^10,11^.

In the terminology of^14^, Theorem 2 states that the computational problem of “possibilistically” simulating the single-qubit gate-teleportation circuit is infeasible for ${{\mathsf{AC}}}^{0}$-circuits of polynomial size. The 1*D*-locality of the single-qubit gate-teleportation is what ultimately yields our fault-tolerant quantum advantage proposal with a 3*D*-local quantum circuit. In contrast, the so-called relaxed parity-halving problem considered by the authors of^6,15^ does not have a simple locality structure, and is arguably more complex.

This quantum advantage demonstration based on the single-qubit gate-teleportation circuit shares a few attractive average-case hardness features with prior work such as^6,16^: The bound on the classical circuits considered here involves the average over a fully random input. In contrast, the results of^5,8^ (as well as our results for the noise-tolerant setup) require restricting to a subset of inputs corresponding to valid problem instances.

## Discussion

We have identified a computational problem for which noisy, 3*D*-local shallow quantum circuits achieve a success probability arbitrarily close to one on uniformly random instances. In contrast, any constant-depth Boolean circuit with unbounded fan-in AND and OR gates, together with NOT gates, i.e., an ${{\mathsf{AC}}}^{0}$-circuit, requires superpolynomial size (in fact, exponential in a fractional power of the input size) to achieve even an arbitrarily small constant success probability on average.

A central ingredient of our construction is the single-qubit gate-teleportation problem. The role of gate teleportation in complexity theory has already been recognized in seminal work by Terhal and DiVincenzo^17^. Theorem 2 adds to this by establishing a new, unconditional complexity-theoretic result. Our work is complementary to considerations in quantum fault-tolerance, where gate teleportation is a key mechanism for achieving computational universality. In contrast, we exploit gate teleportation not as a mechanism for noise resilience, but to define a simple and natural computational problem.

One of the results in ref. ^15^ establishes an advantage of noisy shallow quantum circuits over ${{\mathsf{AC}}}^{0}$ without imposing geometric locality. While their result may superficially appear similar to ours, we emphasize that geometric locality is a substantive feature of Theorem 1, not merely an implementation detail. In an abstract circuit without a specified layout, arbitrary two-qubit gates may be treated as elementary operations, with local stochastic noise^18,19^ modeled between its gate layers (see Supplementary Note[Section 6.1]^9^ for a formal definition of this noise model). A quantum advantage achieved by such an abstract circuit with non-local operations may not translate into an experimentally observable advantage in practice. This is because realizing such a circuit on a realistic architecture restricted to short-range interactions typically necessitates additional routing operations; a straightforward SWAP-based compilation, for example, may cause the depth to grow with the system size. Such additional operations impact the noise resilience, and/or lead to a circuit of non-constant depth. Consequently, a quantum advantage of an abstract constant-depth circuit resilient to local stochastic noise will generally not carry over to a geometrically local implementation.

By establishing the separation directly within the smaller class of 3*D*-local circuits, our result (see Theorem 1) addresses this implementation gap while strengthening the complexity-theoretic separation. To the best of our knowledge, no separation from ${{\mathsf{AC}}}^{0}$ realized by a noise-resilient, 3*D*-local shallow quantum circuit was known prior to this work. A natural open question is whether 2*D*-local shallow quantum circuits subject to local stochastic noise can achieve an analogous advantage against ${{\mathsf{AC}}}^{0}$, or at least against ${{\mathsf{NC}}}^{0}$.

## Methods

Here we sketch the key ideas leading to our results. For a mathematically rigorous treatment see the associated Supplementary Note^9^.

The main technical result enabling us to obtain quantum advantage with noisy 3*D*-local constant-depth quantum circuits against ${{\mathsf{AC}}}^{0}$ circuits and thus Theorem 1 is the average-case quantum advantage with *ideal* 1*D*-local constant-depth quantum circuits against ${{\mathsf{AC}}}^{0}$ circuits, i.e., Theorem 2.

### Idea of the proof of Theorem 2

There are two main techniques to prove circuit lower bounds against ${{\mathsf{AC}}}^{0}$ circuits: (i) restriction-based methods^1,2,20,21^, and (ii) polynomial methods^22,23^. To establish our result we use the former and in particular the so-called switching lemmas^3^. The underlying idea is that a random restriction – meaning a random assignment of fixed values to a sufficiently large subset of inputs – considerably simplifies an ${{\mathsf{AC}}}^{0}$ circuit: With high probability, the resulting restricted circuit has an input-output behavior which coincides with that of an ${{\mathsf{NC}}}^{0}$-circuit. This means that a computational no-go (i.e., hardness) result for ${{\mathsf{NC}}}^{0}$-circuits can be “boosted” to establish hardness against ${{\mathsf{AC}}}^{0}$-circuits provided that the computational problem under consideration has the following property: the induced “restricted" problems obtained by random restriction are computationally infeasible for ${{\mathsf{NC}}}^{0}$-circuits. An example is the problem of computing the parity: fixing some random inputs, the remaining problem here is still that of computing the parity (although of a shorter string), thus infeasible for ${{\mathsf{NC}}}^{0}$-circuits.

To prove Theorem 2, assume for the sake of contradiction that there is an ${{\mathsf{AC}}}^{0}$-circuit which can solve the single-qubit gate teleportation problem with a probability strictly larger than *ν* = 0.986 over a uniformly chosen input. Using our own variant of the switching lemma (which restricts input Cliffords represented by blocks of bits), this implies that there is an ${{\mathsf{NC}}}^{0}$-circuit which solves a (randomly) restricted variant of the single-qubit gate-teleportation problem with high probability, for suitably chosen parameters (see the Supplementary Note[Section 5.1]^9^). We argue that this is a contradiction by showing that any ${{\mathsf{NC}}}^{0}$-circuit in fact fails to solve such a restricted variant of the single-qubit gate-teleportation problem.

To this end, we formulate a two-player quantum non-locality game (i.e., a Bell inequality expressed by differences in winning probabilities between classical and quantum players). Here two players are given Clifford unitaries *U* and *V*, respectively, and are each asked to output a Pauli *P* (depending on the input *U*), and *Q* (depending on the input *V*) such that

2 $${{\rm{tr}}}(UPVQ)\,\ne\, 0.$$

We argue that the (restricted) gate teleportation problem expressed by (1) is closely related to this two-player nonlocal game: any ${{\mathsf{NC}}}^{0}$-circuit for the former can be translated to a classical strategy (i.e., two functions *U* ↦ *P*(*U*) and *V* ↦ *Q*(*V*)) for the latter. This argument relies on the lightcone structure of ${{\mathsf{NC}}}^{0}$-circuits in a similar way as argued in ref. ^8^, but more specifically exploits commutation relations between Cliffords and Paulis (see Supplementary Note[Section 4]^9^). A bound on the success probability of any ${{\mathsf{NC}}}^{0}$-circuit thus results from the fact that the two-player game cannot be won with high probability by two classical players (not sharing entanglement).

To establish this latter fact, i.e., to bound the classical value of the two-player game defined by (2), we relate it to a specific variant of the Magic Square game^24,25^: This game is obtained by restricting to certain pairs of inputs (*U*, *V*). What is special about our variant of the Magic Square game is that it can be won by a particular form of quantum strategy for two players sharing two Bell pairs: Each player only applies a single-qubit Clifford (depending on their input), followed by a Bell measurement and classical post-processing (see Supplementary Note[Section 1]^9^). We show that if the classical players can succeed at the two-local game given by (2) with a probability strictly larger than 35/36 over randomly chosen pair of Cliffords, they can win the Magic Square game with probability strictly larger than 8/9 (we note that the slight discrepancy between 0.986 in Theorem 2 and 35/36 results from the extra overhead incurred when mapping Clifford gates to the binary representation used by the ${{\mathsf{AC}}}^{0}$ circuit).

**Fig. 2:**
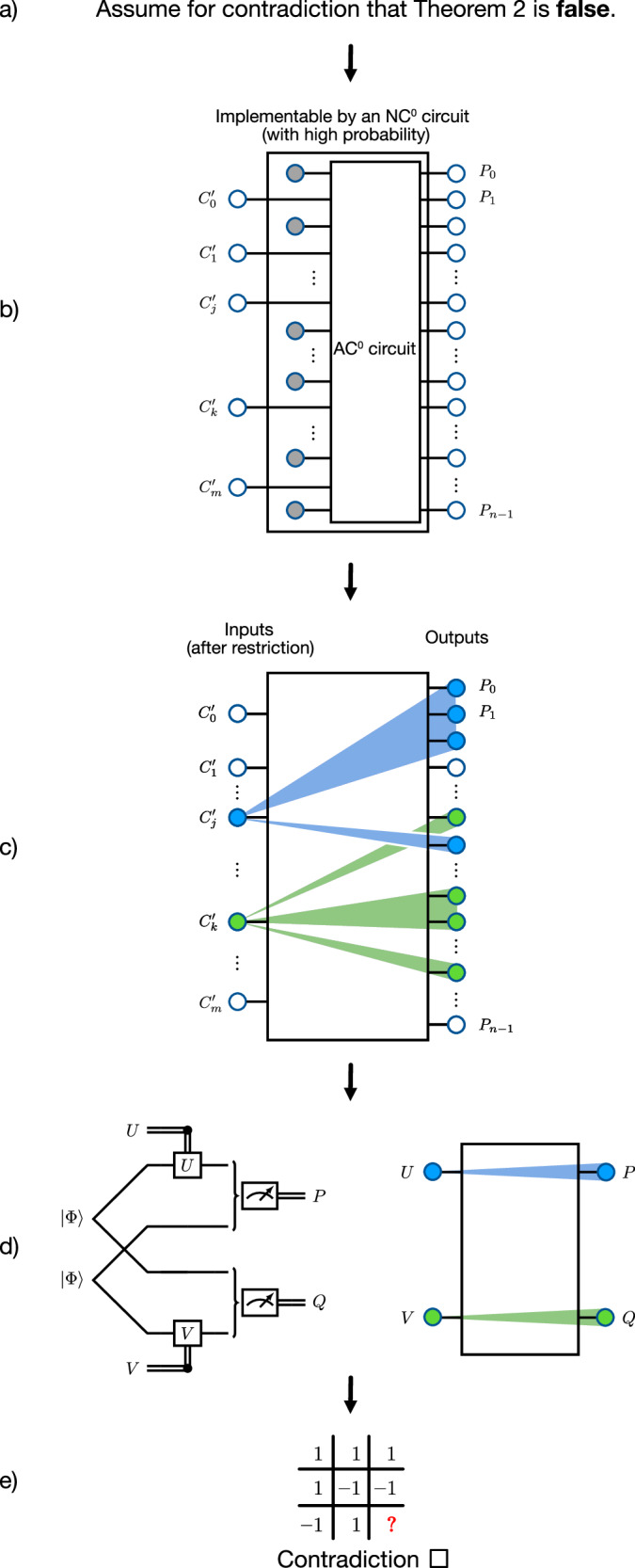
Graphical depiction of the key steps of the proof of Theorem 2. **a** For contradiction, suppose that Theorem 2 is false; **b** by our variant of switching lemma (randomly restricting input Cliffords as blocks of bits) we argue that with high probability there is an ${{\mathsf{NC}}}^{0}$ circuit for a randomly restricted variant of the single-qubit gate-teleportation problem (gray Clifford inputs are assigned randomly); **c** such a circuit has necessarily two inputs with non-intersecting lightcones, illustrated in blue and green; **d** by commutation relations and the cyclic structure of the problem we can relate it to a circuit solving a specific variant of the Magic Square game where each player can apply only a single-qubit Clifford gate; **e** classical postprocessing yields a classical strategy for the Magic Square game that wins with probability strictly larger than 8/9. This is a contradiction.

This completes the sketch of the proof of Theorem 2. We illustrate the key steps in Fig. 2.

### Idea of the proof of Theorem 1

To establish our main result – a quantum advantage with noisy constant-depth 3*D*-local quantum circuits against ${{\mathsf{AC}}}^{0}$ – we borrow non-standard fault-tolerance techniques developed in ref. ^8^. These techniques eliminate the non-constant depth overhead typical of standard fault-tolerance schemes for universal quantum computation with geometrically local circuits. This is achieved by incorporating parts of the fault-tolerance construction such as the decoding problem or correction operations for constant-depth logical state preparation directly into the computational problem considered.

The construction starts with an underlying computational problem and an ideal quantum circuit which solves it with certainty. In our case, these are the single-qubit gate- teleportation problem from Theorem 2 and the associated quantum circuit in Fig. 1. The result of the construction is a modified computational problem and an associated quantum circuit resilient against local stochastic noise below a certain threshold.

To achieve a quantum advantage with noisy constant-depth 3*D*-local circuits, this non-standard approach relies on a number of conditions on the underlying computational problem and on the ideal quantum circuit solving it. Our construction in Theorem 2 meets the following requirements:

(i) the implemented quantum circuit contains only classically controlled Clifford gates,
(ii) the implemented quantum circuit has a 1*D*-local geometry, and
(iii) the targeted complexity-theoretic separation allows to include certain computations such as the decoding into the problem definition.

**Fig. 3:**
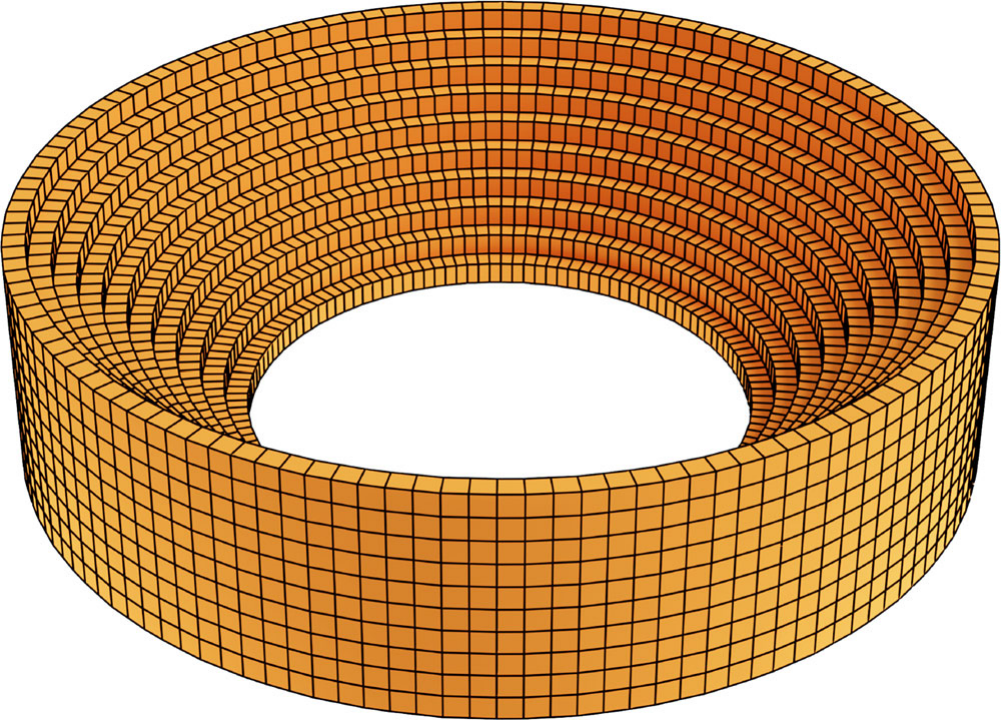
Colosseum-shaped qubit layout of the fault-tolerant circuit. This arrangement allows the circuit to be realized using geometrically local interactions in three dimensions.

This construction leads to the 3*D*-local Colosseum-shaped fault-tolerant quantum circuit, see Fig. 3, which solves a modified single-qubit gate-teleportation problem.

In Section 6 of Supplementary Note^9^ we summarize the techniques of ref. ^8^ and establish the fault-tolerant quantum advantage that underlies Theorem 1.

## Supplementary information

Supplementary Information

Transparent Peer Review file
