# Supplementary material for: 3D-local noisy shallow quantum circuits defeat unbounded fan-in classical circuits
Source: Nat Commun. 2026 Aug 11;17:8174. doi: 10.1038/s41467-026-76560-x (PMC13463102; doi:10.1038/s41467-026-76560-x)
Supplement: Supplementary file 1 [file 41467_2026_76560_MOESM1_ESM.pdf]

Supplementary Note

3D-local noisy shallow quantum circuits defeat unbounded fan-in classical circuits

Libor Caha, Xavier Coiteux-Roy, and Robert König

School of Computation, Information and Technology, Technical University of Munich & Munich Center for Quantum Science and Technology, Munich, Germany.

July 21, 2026

Abstract

In this Supplementary Note we provide additional material and detailed proofs for the claims made in the main paper. The presentation here is self-contained and can be studied independently of the latter.

Contents

1 The magic-square game and its variants 2

1.1 Pseudo-telepathy games and the magic square . . . 2

1.2 Pseudo-telepathy games with single-qubit control . . . 2

2 Single-qubit gate teleportation as a relation problem 8

3 Quantum advantage against NC^0 8

3.1 Average-case hardness of $R_{U_n^{\mathsf{Telep}}}$ for NC^0-circuit . . . 9

4 Towards proving an advantage against AC^0: variants of the quantum advantage against NC^0 15

4.1 Average-case hardness of $R_{U_n^{\mathsf{Telep}}}\restriction_\xi$ for NC^0-circuit . . . 16

4.2 From Clifford inputs to input strings . . . 19

4.3 Average-case hardness of $R_{U_n^{\mathsf{Telep}}}\restriction_\xi$ with bit encoded input . . . 20

5 Quantum advantage against AC^0 22

5.1 Bit-restrictions and block-restrictions . . . 22

5.2 From an AC^0 circuit to an NC^0 circuit by random restrictions . . . 23

5.3 An AC^0 circuit size lower bound for the single-qubit gate-teleportation problem . 27

6 Quantum advantage with noisy shallow 3D-local circuits against AC^0 30

6.1 Noisy quantum circuits: Local stochastic noise . . . 30

6.2 Fault-tolerance building blocks . . . 30

6.3 3D-local fault-tolerant building blocks . . . 34

6.4 Rendering quantum advantages noise-resilient . . . 38

6.5 Noise-resilient quantum advantage against AC^0 with a $3D$-local architecture . . . 39

7 Amplification of the classical–quantum gap **41**
7.1 Parallel repetition for the single-qubit-control pseudo-telepathy game 42
7.2 Hardness amplification for NC^0 and AC^0 circuits 44
7.3 Amplified noise-resilient quantum advantage against AC^0 with $3D$-local architecture 48

Supplementary References **49**

1 The magic-square game and its variants

Our demonstration of quantum advantage is built upon a connection between the single-qubit teleportation circuit and pseudo-telepathy games.

1.1 Pseudo-telepathy games and the magic square

Pseudo-telepathy games are non-local games that cannot be won with certainty by classical players with shared randomness, yet can be won with certainty by quantum players sharing entanglement. The celebrated magic-square game [20, 18] is an example of such a game in the bipartite setting. In this game, Alice's input is an element $\alpha \in \{1, 2, 3\}$, whereas Bob's input is $\beta \in \{1, 2, 3\}$. Alice outputs $(x_1, x_2, x_3) \in \{-1, 1\}^3$ and Bob outputs $(y_1, y_2, y_3) \in \{-1, 1\}^3$. The two players win the game if

$$\begin{aligned} x_1x_2x_3 &= \ \ 1 \\ y_1y_2y_3 &= -1 \end{aligned} \qquad \text{and} \qquad y_\alpha x_\beta = \ 1\ . \tag{1}$$

$$\begin{array}{ccc} X \otimes I & I \otimes X & X \otimes X \\ I \otimes Z & Z \otimes I & Z \otimes Z \\ -X \otimes Z & -Z \otimes X & Y \otimes Y \end{array}$$

Supplementary Figure 1: These Pauli observables form a magic square: the product of any row of observables is $I \otimes I$, while the product of any column of observables is $-I \otimes I$.

Two non-communicating classical players can win this game for a uniformly chosen instance $(\alpha, \beta) \in \{1, 2, 3\}^2$ with probability at most 8/9. In contrast, the game can be won with certainty by players sharing two copies $\Phi_{A_1B_1} \otimes \Phi_{A_2B_2}$ of the maximally entangled state $|\Phi\rangle = \frac{1}{\sqrt{2}}(|00\rangle + |11\rangle)$. A corresponding protocol can be obtained from the "magic square," see Supplementary Figure 1. On input $\alpha \in \{1, 2, 3\}$, Alice performs a measurement of the three commuting observables in the α-th row of the square, outputting the corresponding eigenvalues. Similarly, on input $\beta \in \{1, 2, 3\}$, Bob measures the three commuting observables in the β-th column of the square to obtain (y_1, y_2, y_3).

1.2 Pseudo-telepathy games with single-qubit control

Here we need a special variant of the magic-square game. It has the special feature that there is a quantum strategy that always allows Alice and Bob to choose the correct measurement basis by making a Bell measurement preceded by a single-qubit rotation depending only on their respective input.

In more detail, we consider two sets $\{U_\alpha\}_{\alpha=1}^3$ and $\{V_\beta\}_{\beta=1}^3$ of single-qubit unitaries defined as follows. For $P \in \{X, Y, Z\}$, define the Clifford gate

$$R_P = \frac{1}{\sqrt{2}}(I - iP)\ .$$

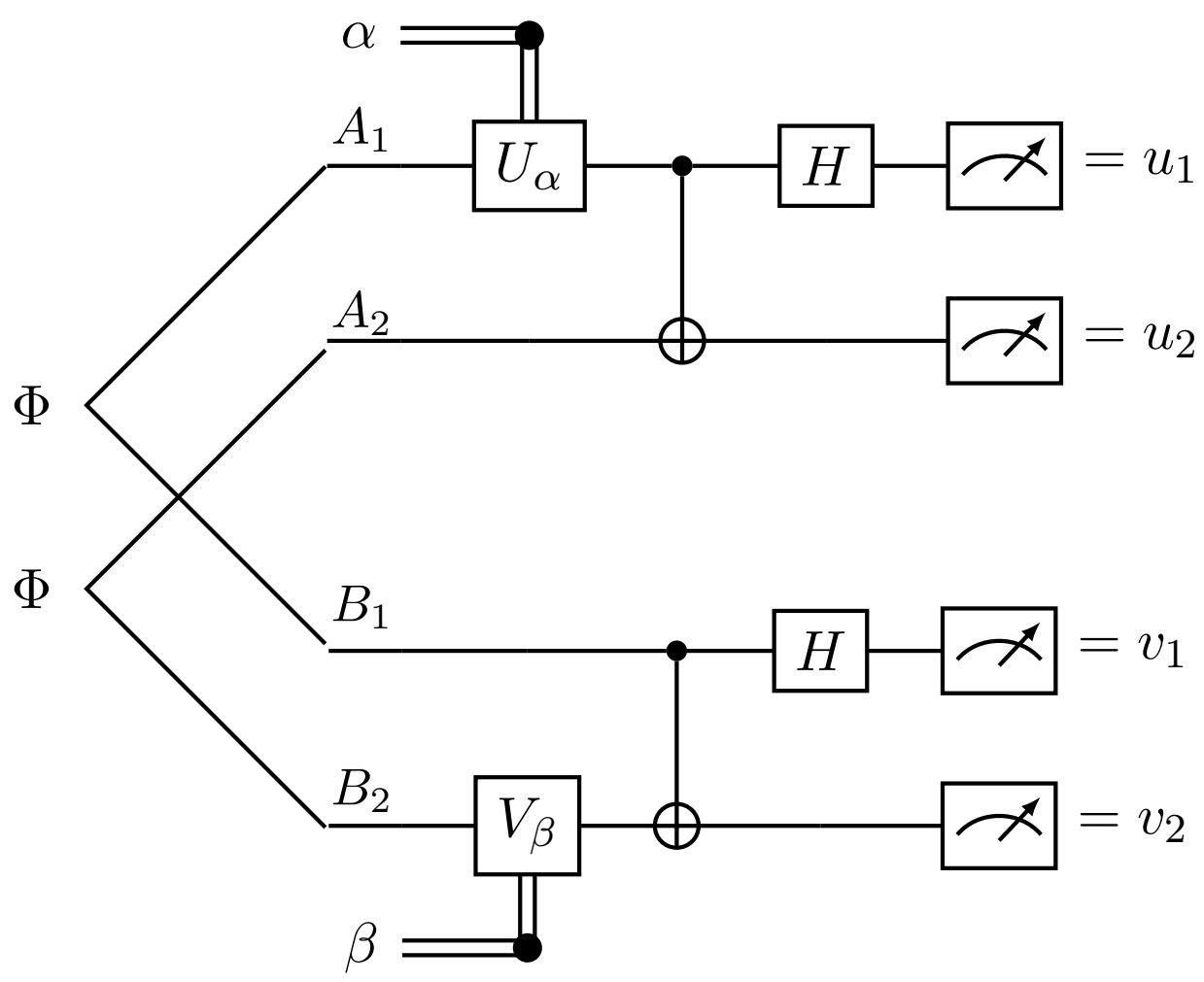

Supplementary Figure 2: Quantum strategy for the pseudotelepathy game $\mathcal{G}$.

(Conjugation by R_P realizes a counterclockwise rotation of the Bloch sphere around the axis defined by P with angle $\pi/2$.) We then define the single-qubit Clifford unitaries

$$(U_1, U_2, U_3) = (R_X, ZR_Y^\dagger, R_Z^\dagger)$$

and

$$(V_1, V_2, V_3) = (I, R_Z R_Y^\dagger, R_X^\dagger R_Y) \ .$$

Consider the following procedure:

(i) Alice and Bob share two copies $\Phi_{A_1B_1} \otimes \Phi_{A_2B_2}$ of the maximally entangled state Φ.

(ii) On input $\alpha \in \{1, 2, 3\}$, Alice applies the unitary U_α to system A_1.

(iii) Similarly, on input $\beta \in \{1, 2, 3\}$, Bob applies the unitary V_β to system B_2.

(iv) Alice performs a Bell basis change by applying $(H \otimes I)_{A_1A_2}\mathsf{CNOT}_{A_1A_2}$. She then measures in the computational basis, obtaining an outcome $(u_1, u_2) \in \{0, 1\}^2$.

(v) Bob similarly applies $(H \otimes I)_{B_1B_2}\mathsf{CNOT}_{B_1B_2}$ and subsequently measures in the computational basis, obtaining an outcome $(v_1, v_2) \in \{0, 1\}^2$.

This strategy is illustrated in Supplementary Figure 2. We argue the following:

Lemma 1.1. *The described protocol is a quantum strategy of a pseudotelepathy game $\mathcal{G}$ which achieves winning probability* 1*. For $(\alpha, \beta) \in \{1, 2, 3\}^2$ chosen uniformly at random, two non-communicating classical players can win $\mathcal{G}$ with probability at most* 8/9*.*

Proof. We will argue that the outputs of Alice and Bob produced by the described protocol can be post-processed locally to provide a solution to the standard magic-square problem. That is, there are functions $\{f_\alpha\}_{\alpha\in\{1,2,3\}}$ with $f_\alpha : \{0, 1\}^2 \to \{-1, 1\}^3$ and functions $\{g_\beta\}_{\beta\in\{1,2,3\}}$ where $g_\beta : \{0, 1\}^2 \to \{-1, 1\}^3$ such that the following holds. Suppose the protocol produces outputs $(u_1, u_2), (v_1, v_2)$ on input $(\alpha, \beta) \in \{1, 2, 3\}^2$. Set

$$x = f_\alpha(u_1, u_2) \qquad \text{and} \qquad y = g_\beta(v_1, v_2) \ . \tag{2}$$

$$\begin{array}{ccc} X_{A_1}X_{A_2} & Y_{A_1}Z_{A_2} & Z_{A_1}Y_{A_2} \\ Y_{A_1}Y_{A_2} & Z_{A_1}X_{A_2} & X_{A_1}Z_{A_2} \\ Z_{A_1}Z_{A_2} & X_{A_1}Y_{A_2} & Y_{A_1}X_{A_2} \end{array}$$

Supplementary Figure 3: We consider a modified version of the magic square as given by these operators. Again, the product of any row of observables is I and the product of any column of observables is $-I$. We consider operators acting on Alice's system only here.

Then $x = (x_1, x_2, x_3)$ and $y = (y_1, y_2, y_3)$ satisfy the winning condition (1) of the magic-square game.

The fact that any output of the described quantum protocol yields a solution to the magic-square game by local processing implies that producing such an output with certainty is infeasible for non-communicating classical players. In other words, we can define a non-local game $\mathcal{G}$ where the task is to "possibilistically" simulate the circuit in Supplementary Figure 2: Given $(\alpha, \beta) \in \{1,2,3\}^2$, the task is to output a pair $(u, v) \in \{0,1\}^2 \times \{0,1\}^2$ that occurs with non-zero probability in the output distribution of the circuit. Because of the existence of the functions $\{f_\alpha\}_\alpha, \{g_\beta\}_\beta$, i.e., the relation with the magic-square game, the game $\mathcal{G}$ can only be won with probability 8/9 by classical strategies, yet can (trivially) be won with certainty by the quantum strategy illustrated in Supplementary Figure 2.

It remains to show the existence of functions $\{f_\alpha\}_{\alpha\in\{1,2,3\}}$ and $\{g_\beta\}_{\beta\in\{1,2,3\}}$ with the desired property. To do so, we relate the measurement result to the magic square of operators given in Supplementary Figure 3.

Let us first consider Alice's measurement results $(u_1, u_2) \in \{0,1\}^2$ on input $\alpha \in \{1,2,3\}$. They correspond to the eigenvalues $(-1)^{u_1}$ and $(-1)^{u_2}$ of the observables $\tilde{U}_\alpha^\dagger(Z\otimes I)\tilde{U}_\alpha$ and $\tilde{U}_\alpha^\dagger(I\otimes Z)\tilde{U}_\alpha$, where

$$\tilde{U}_\alpha = (H\otimes I)\mathsf{CNOT}(U_\alpha\otimes I)\ .$$

Straightforward computation then gives the following table:

α	U_α	$(-1)^{u_1}$ eigenvalue of	$(-1)^{u_2}$ eigenvalue of
1	R_X	$X_{A_1}X_{A_2}$	$Y_{A_1}Z_{A_2}$
2	$ZR_Y^\dagger$	$Z_{A_1}X_{A_2}$	$X_{A_1}Z_{A_2}$
3	$R_Z^\dagger$	$Y_{A_1}X_{A_2}$	$Z_{A_1}Z_{A_2}$

Observe that for each $\alpha \in \{1,2,3\}$, the measurement outcomes (u_1, u_2) directly determine the eigenvalues of two observables in row α of the table given in Supplementary Figure 3. The eigenvalue of the third observable is obtained by taking the product $(-1)^{u_1+u_2}$ since the product of these observables is I. In other words, the list of eigenvalues is given by

$\alpha=1$	$X_{A_1}X_{A_2}$	$Y_{A_1}Z_{A_2}$	$Z_{A_1}Y_{A_2}$	$((-1)^{u_1},(-1)^{u_2},(-1)^{u_1+u_2}) =: f_1(u_1,u_2)$
$\alpha=2$	$Y_{A_1}Y_{A_2}$	$Z_{A_1}X_{A_2}$	$X_{A_1}Z_{A_2}$	$((-1)^{u_1+u_2},(-1)^{u_1},(-1)^{u_2}) =: f_2(u_1,u_2)$
$\alpha=3$	$Z_{A_1}Z_{A_2}$	$X_{A_1}Y_{A_2}$	$Y_{A_1}X_{A_2}$	$((-1)^{u_2},(-1)^{u_1+u_2},(-1)^{u_1}) =: f_3(u_1,u_2)$

Here we defined the functions $\{f_\alpha\}_{\alpha\in\{1,2,3\}}$ in such a way that $f_\alpha(u_1,u_2)$ is the triple of eigenvalues associated with the row α in Supplementary Figure 3.

Similarly, the measurement results $(v_1, v_2) \in \{0,1\}^2$ of Bob correspond to eigenvalues $(-1)^{v_1}$ and $(-1)^{v_2}$ of the observables $\tilde{V}_\beta^\dagger(Z\otimes I)\tilde{V}_\beta$ and $\tilde{V}_\beta^\dagger(I\otimes Z)\tilde{V}_\beta$, where

$$\tilde{V}_\beta = (H\otimes I)\mathsf{CNOT}(I\otimes V_\beta)\ ,$$

that is,

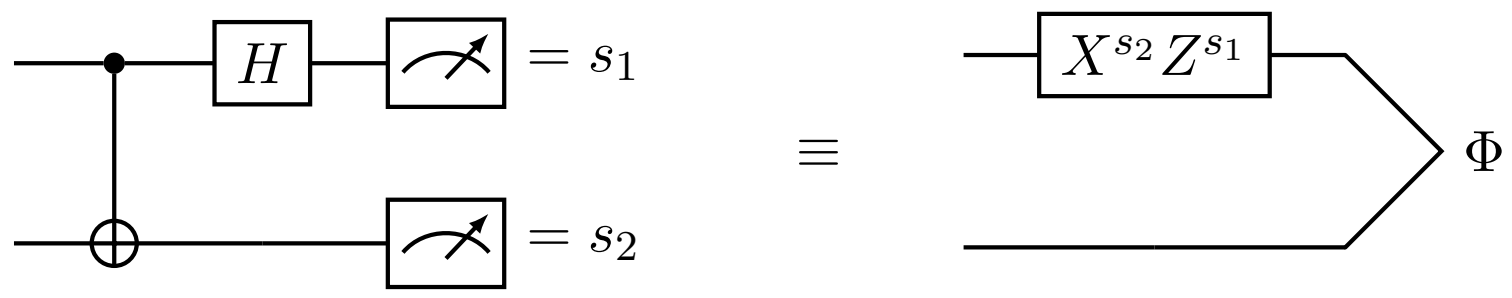

Supplementary Figure 4: Bell measurement: Instead of labeling outcomes by $(s_1, s_2) \in \{0,1\}^2$, we may equivalently use $P \in \{I, X, Y, Z\}$. The outcome (s_1, s_2) corresponds to the Pauli $X^{s_2}Z^{s_1}$. Overall signs can be ignored.

β	V_β	$(-1)^{v_1}$ eigenvalue of	$(-1)^{v_2}$ eigenvalue of
1	I	$X_{B_1}X_{B_2}$	$Z_{B_1}Z_{B_2}$
2	$R_Z R_Y^\dagger$	$-X_{B_1}Y_{B_2}$	$Z_{B_1}X_{B_2}$
3	$R_X^\dagger R_Y$	$X_{B_1}Z_{B_2}$	$-Z_{B_1}Y_{B_2}$

Similarly as before, we can define functions $\{g_\beta\}_{\beta\in\{1,2,3\}}$ such that $g_\beta(v_1, v_2)$ corresponds to a triple of eigenvalues of commuting operators whose product is $-I$: We have

$\beta = 1$	$X_{B_1}X_{B_2}$	$Y_{B_1}Y_{B_2}$	$Z_{B_1}Z_{B_2}$	$((-1)^{v_1}, -(-1)^{v_1+v_2}, (-1)^{v_2}) =: g_1(v_1, v_2)$
$\beta = 2$	$-Y_{B_1}Z_{B_2}$	$Z_{B_1}X_{B_2}$	$-X_{B_1}Y_{B_2}$	$(-(-1)^{v_1+v_2}, (-1)^{v_1}, (-1)^{v_2}) =: g_2(v_1, v_2)$
$\beta = 3$	$-Z_{B_1}Y_{B_2}$	$X_{B_1}Z_{B_2}$	$-Y_{B_1}X_{B_2}$	$((-1)^{v_2}, (-1)^{v_1}, -(-1)^{v_1+v_2}) =: g_3(v_1, v_2)$

It is clear from these definitions that (x, y) computed according to (2) satisfy the conditions $x_1x_2x_3 = 1$ and $y_1y_2y_3 = -1$.

To check the remaining condition $y_\alpha x_\beta = 1$, observe that for any $\beta \in \{1, 2, 3\}$, the three relevant observables correspond to the β-th column of the magic square in Supplementary Figure 3 up to signs depending on the number (parity) of Pauli-Y-operators, and the fact that these act on B_1B_2 instead of A_1A_2. In our quantum strategy, the state being measured is $\Phi_{A_1B_1} \otimes \Phi_{A_2B_2}$. Because the maximally entangled state Φ satisfies

$$(\Gamma \otimes I)\Phi = (I \otimes \Gamma^T)\Phi \qquad \text{for any} \qquad \Gamma \in \mathsf{Mat}_{2\times 2}(\mathbb{C}) \ , \tag{3}$$

and $Y^T = -Y$ is antisymmetric (whereas $X^T = X$ and $Z^T = Z$ are symmetric), it follows that the eigenvalues computed from (v_1, v_2) using g_β actually correspond to the operators in the β-th column in Supplementary Figure 3 (i.e., the operators act on A_1A_2 and there are no signs). The claim follows from this. □

Let us state the definition of the game $\mathcal{G}$ more explicitly. For inputs $(\alpha, \beta) \in \{1, 2, 3\}^2$, the task is to output $((u_1, u_2), (v_1, v_2)) \in \{0,1\}^2 \times \{0,1\}^2$ such that the outcome probability

$$p_{\alpha,\beta}(u_1, u_2, v_1, v_2) = \left| (\langle u_1, u_2| \otimes \langle v_1, v_2|)(\tilde{U}_\alpha^{A_1A_2} \otimes \tilde{V}_\beta^{B_1B_2})(|\Phi_{A_1B_1}\rangle \otimes |\Phi_{A_2B_2}\rangle) \right|^2 \tag{4}$$

is non-zero.

In the following, we will rephrase the game $\mathcal{G}$ slightly in order to simplify the notation. To this end, observe that the steps (iv) and (v) realize Bell measurements: We have

$$(\langle s_1| \otimes \langle s_2|)\mathsf{CNOT}(H \otimes I) \propto \langle \Phi| (X^{s_2}Z^{s_1} \otimes I) \qquad \text{for all} \qquad (s_1, s_2) \in \{0,1\}^2 \ ,$$

see Supplementary Figure 4. That is, every outcome (s_1, s_2) is uniquely associated with a Pauli operator $P \propto X^{s_2}Z^{s_1}$. The overall phase of P is irrelevant here and we may without loss of generality assume that $P \in \{I, X, Y, Z\} =: \mathsf{Pauli}$. In other words, we are performing a von Neumann measurement with operators $\{|\Phi_P\rangle\langle\Phi_P|\}_{P\in\mathsf{Pauli}}$, where $\Phi_P := (P \otimes I)\Phi$ for $P \in \mathsf{Pauli}$ defines the Bell basis.

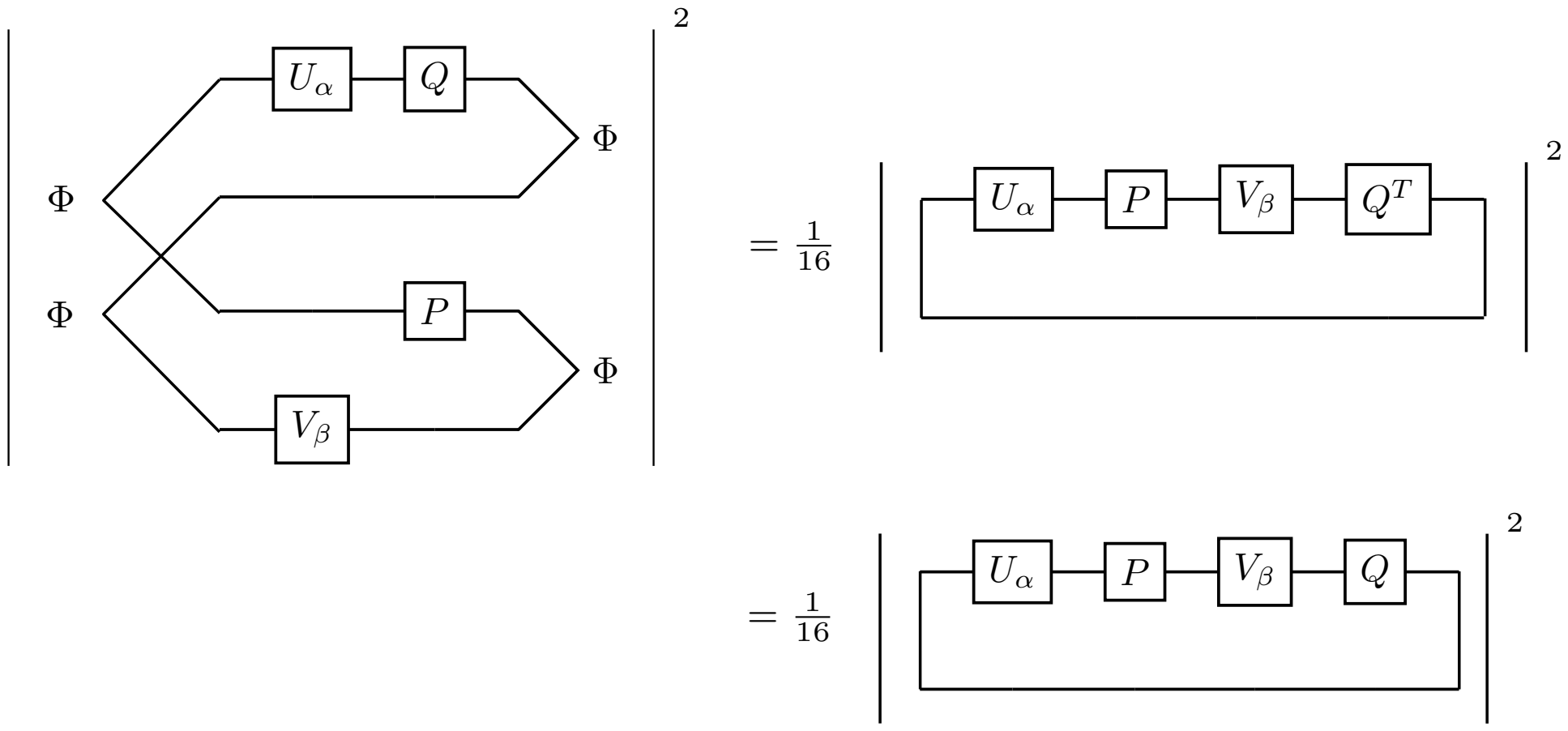

Supplementary Figure 5: Probability $p_{\alpha,\beta}(P,Q)$ of the outcome $(P,Q) \in \mathsf{Pauli}^2$ for the circuit (2) when expressing Bell measurements in terms of Pauli outcomes.

In the following, we directly use the set Pauli to label measurement outcomes instead of using two bits for each Pauli operator. For the game $\mathcal{G}$, this means that instead of asking for an output of the form $((u_1,u_2),(v_1,v_2)) \in \{0,1\}^2 \times \{0,1\}^2$, we seek $(P,Q) \in \mathsf{Pauli}^2$ (where P is associated with (u_1,u_2) and Q with (v_1,v_2), respectively). The corresponding output probability (4) then takes the form

$$p_{\alpha,\beta}(P,Q) = \left|(\langle\Phi_P|_{A_1A_2}\langle\Phi_Q|_{B_1B_2})(U_\alpha^{A_1} \otimes I_{A_2} \otimes I_{B_1} \otimes V_\beta^{B_2})\left(|\Phi\rangle_{A_1B_1}|\Phi\rangle_{A_2B_2}\right)\right|^2$$

for $(P,Q) \in \mathsf{Pauli}^2$. A diagrammatic representation of the probability $p_{\alpha,\beta}(P,Q)$ is given in Supplementary Figure 5. It shows that the probability of interest can be written as

$$p_{\alpha,\beta}(P,Q) = \frac{1}{16}\left|\mathrm{tr}(U_\alpha P V_\beta Q)\right|^2 \tag{5}$$

Formally, this follows from the substitution rule (3) and the fact that $P^T \in \{\pm P\}$ for every $P \in \mathsf{Pauli}$.

In the following, let $\mathsf{Cliff} := \mathsf{Pauli} \cdot \{I, H, S, HS, SHS, (HS)^2\}$ denote the 1-qubit Clifford group modulo global phases. Such phases are irrelevant in our considerations. (As for Paulis, we often represent elements of Cliff by bitstrings but leave this implicit to avoid clutter. Since $|\mathsf{Cliff}| = 24$, 5 bits are sufficient.) Then we can formulate the following corollary, which will be an essential tool in our analysis.

Corollary 1.2. *Let $F : \{1,2,3\} \to \mathsf{Pauli}$ and $G : \{1,2,3\} \to \mathsf{Pauli}$ be arbitrary functions. Then there exists at least one pair $(\alpha,\beta) \in \{1,2,3\}^2$ such that*

$$\mathrm{tr}(U_\alpha F(\alpha) V_\beta G(\beta)) = 0 \ .$$

Proof. For the sake of contradiction, suppose that

$$\mathrm{tr}(U_\alpha F(\alpha) V_\beta G(\beta)) \neq 0 \qquad \text{for all} \qquad (\alpha,\beta) \in \{1,2,3\}^2 \ . \tag{6}$$

Consider the classical, local (non-communicating) strategy where Alice, on input α, outputs $F(\alpha)$, and Bob, on input β, outputs $G(\beta)$. By the assumption (6) and Eq. (5), we get $p_{\alpha,\beta}(F(\alpha),G(\beta)) \neq 0$ for all $(\alpha,\beta) \in \{1,2,3\}^2$. That is, this strategy succeeds with certainty, contradicting Lemma 1.1. □

We generalize the previous corollary to uniformly random Clifford inputs.

Lemma 1.3. *Let* $F' : \mathsf{Cliff} \to \mathsf{Pauli}$ *and* $G' : \mathsf{Cliff} \to \mathsf{Pauli}$ *be arbitrary functions. Then*

$$\Pr_{(U,V)\in\mathsf{Cliff}^2}\left[\mathrm{tr}(UF'(U)VG'(V)) = 0\right] \geq \frac{1}{36} \ . \tag{7}$$

In Section 7, we argue that the constant 1/36 can be replaced by any constant (arbitrarily close to 1), see Lemma 7.2. This involves the use of the parallel repetition theorem for two-prover one-round games by Raz [22].

Proof. Define the set

$$\Gamma := \left\{(U,V) \in \mathsf{Cliff}^2 \mid \mathrm{tr}\left(UF'(U)VG'(V)\right) = 0\right\} \ .$$

By Corollary 1.2, there is at least one pair $(\alpha, \beta) \in \{1,2,3\}^2$ such that $(U_\alpha, V_\beta) \in \Gamma$, i.e., $|\Gamma| \geq 1$. We can improve this lower bound as follows.

Let $(P,Q) \in \mathsf{Pauli}^2$ be arbitrary. Define $F, G : \{1,2,3\} \to \mathsf{Pauli}$ by setting

$$F(\alpha) := PF'(U_\alpha P) \qquad \text{and} \qquad G(\beta) := QG'(V_\beta Q)$$

for $(\alpha, \beta) \in \{1,2,3\}^2$. By Corollary 1.2, it follows that there exist $(\alpha, \beta) \in \{1,2,3\}^2$ such that

$$\mathrm{tr}(U_\alpha F(\alpha) V_\beta G(\beta)) = \mathrm{tr}(U_\alpha P F'(U_\alpha P) V_\beta Q G'(V_\beta Q)) = 0 \ .$$

This shows that for every pair $(P,Q) \in \mathsf{Pauli}^2$ there is a pair $(\alpha_{P,Q}, \beta_{P,Q}) \in \{1,2,3\}^2$ such that

$$(U_{\alpha_{P,Q}} P, V_{\beta_{P,Q}} Q) \in \Gamma \ .$$

For each (P,Q), choose one such pair $(\alpha_{P,Q}, \beta_{P,Q})$. It is easy to check that U_1, U_2, U_3 lie in pairwise distinct right Pauli-cosets of Cliff (and similarly for V_1, V_2, V_3). Therefore, the map

$$(P,Q) \mapsto \left(U_{\alpha_{P,Q}} P, V_{\beta_{P,Q}} Q\right)$$

is injective: equality of two images forces equality of the corresponding right Pauli-cosets in both coordinates, hence equality of the corresponding indices, and then equality of the Paulis. We thus have

$$\left|\left\{(U_{\alpha_{P,Q}} P, V_{\beta_{P,Q}} Q) \mid (P,Q) \in \mathsf{Pauli}^2\right\}\right| = 16 \ ,$$

and therefore

$$|\Gamma| \geq 16 \ .$$

The claim (7) now follows because

$$\Pr_{(U,V)\in\mathsf{Cliff}^2}\left[(U,V) \in \Gamma\right] = \frac{|\Gamma|}{24^2} \geq \frac{16}{24^2} \ .$$

□

2 Single-qubit gate teleportation as a relation problem

The computational problem we use to establish complexity-theoretic separations is a relation (also called search) problem. Such a problem is defined by a subset $R \subset \mathcal{I} \times \mathcal{O}$ of input/output pairs. For a given input $i \in \mathcal{I}$, the task is to output $o \in \mathcal{O}$ such that $(i, o) \in R$. We call any $o \in \mathcal{O}$ with this property a valid solution to the input instance $i \in \mathcal{I}$.

In our case, the relation is indexed by its size $n \in \mathbb{N}$. We denote it by $R_{U_n^{\mathsf{Telep}}}$ for reasons that will be clarified below. The associated input set is the set

$$\mathsf{Cliff}^n = \{(C_0, \ldots, C_{n-1}) \mid C_j \in \mathsf{Cliff} \text{ for any } j \in \mathbb{Z}_n\}$$

of n-tuples of single-qubit Clifford group elements. Here and below we use indices $j \in \mathbb{Z}_n$ instead of $[n] = \{1, \ldots, n\}$ to emphasize a certain cyclic symmetry of the problem. Similarly, the associated output set of the problem is the set of n-tuples

$$\mathsf{Pauli}^n = \{(P_0, \ldots, P_{n-1}) \mid P_j \in \mathsf{Pauli} \text{ for any } j \in \mathbb{Z}_n\}$$

of single-qubit Pauli elements. The relation $R_{U_n^{\mathsf{Telep}}} \subset \mathsf{Cliff}^n \times \mathsf{Pauli}^n$ is now defined as follows: We have

$$((C_0, \ldots, C_{n-1}), (P_0, \ldots, P_{n-1})) \in R_{U_n^{\mathsf{Telep}}} \qquad \text{if and only if} \qquad \mathrm{tr}(P_{n-1}C_{n-1} \cdots P_0 C_0) \neq 0 \ .$$

Equivalently, we may say that the computational problem is to output, for a given n-tuple $(C_0, \ldots, C_{n-1})$ of single-qubit Clifford unitaries, a sequence $(P_0, \ldots, P_{n-1})$ of Pauli operators such that the quantity

$$p(P_0, \ldots, P_{n-1} | C_0, \ldots, C_{n-1}) := 4^{-n} \left|\mathrm{tr}(P_{n-1}C_{n-1} \cdots P_0 C_0)\right|^2 \tag{8}$$

is non-zero. According to this reformulation and simple algebra, the computational problem given by the relation $R_{U_n^{\mathsf{Telep}}}$ is that of "possibilistically" simulating the output distribution of the gate-teleportation circuit U_n^{Telep} given in Supplementary Figure 6a. Indeed, Eq. (8) is the outcome distribution of U_n^{Telep} when the state prepared by U_n^{Telep} with input $(C_0, \ldots, C_{n-1})$ is measured using Bell measurements. This means that $R_{U_n^{\mathsf{Telep}}}$ is the problem of producing, for a given input $(C_0, \ldots, C_{n-1})$, an output $(P_0, \ldots, P_{n-1})$ that occurs with non-zero probability in the output distribution of the circuit U_n^{Telep}. For this reason, we will refer to the problem defined by $R_{U_n^{\mathsf{Telep}}}$ as the possibilistic simulation of the single-qubit gate-teleportation circuit. This computational problem was introduced in Ref. [9].

Because the circuit U_n^{Telep} is a constant-depth quantum circuit with nearest-neighbor gates arranged on a circle, we can observe the following:

Theorem 2.1 (Relation problem $R_{U_n^{\mathsf{Telep}}}$ is contained in $1D$-local QNC^0)**.** *There is a classically controlled Clifford circuit U_n^{Telep} which is $1D$-local and of constant depth, and which solves the relation problem $R_{U_n^{\mathsf{Telep}}}$ with certainty for any input $C = (C_0, \ldots, C_{n-1}) \in \mathsf{Cliff}^n$, i.e., it produces $P = (P_0, \ldots, P_{n-1}) \in \mathsf{Pauli}^n$ such that $(C, P) \in R_{U_n^{\mathsf{Telep}}}$.*

3 Quantum advantage against NC^0

In the following, we consider the hardness of possibilistically simulating the gate-teleportation circuit by classical circuits. Without loss of generality, we can restrict to deterministic circuits. A classical circuit $\mathcal{C}_n$ for the problem $R_{U_n^{\mathsf{Telep}}}$ can then be seen as a function $\mathcal{C}_n : \mathsf{Cliff}^n \to \mathsf{Pauli}^n$.

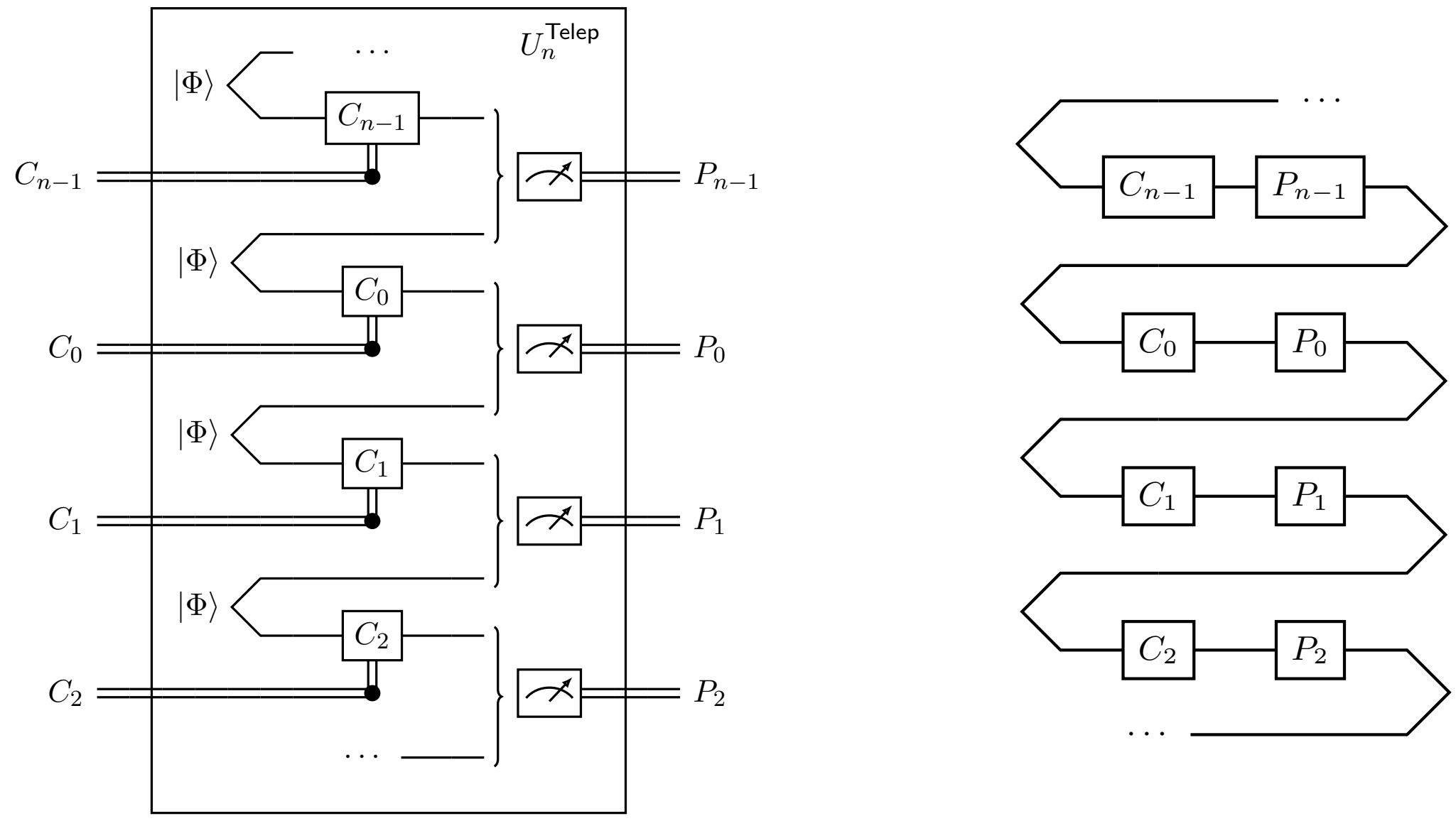

(a) The gate-teleportation circuit U_n^{Telep} is a classically controlled Clifford circuit. It takes as input n Clifford group elements $(C_0, \dots, C_{n-1})$. Each of these is applied to half of a maximally entangled state Φ. If Bell measurements are performed on pairs of qubits (shifted by one), the output $(P_0, \dots, P_{n-1})$ is an n-tuple of Paulis jointly distributed according to Eq. (8).

(b) Tensor-network representation of the gate-teleportation circuit U_n^{Telep}. The Clifford gates $\{C_i\}$ represent the input to the circuit, while the Pauli gates $\{P_i\}$ represent the output.

Supplementary Figure 6: Single-qubit gate teleportation circuit.

3.1 Average-case hardness of $R_{U_n^{\mathsf{Telep}}}$ for NC^0-circuit

In this section, we show an average-case lower bound on the circuit depth of any bounded fan-in classical circuit solving $R_{U_n^{\mathsf{Telep}}}$ for a uniformly random input.

Theorem 3.1. *Let $\mathcal{C}_n : \mathsf{Cliff}^n \to \mathsf{Pauli}^n$ be a circuit with gates of fan-in bounded by a constant K, such that*

$$\Pr_{C \in \mathsf{Cliff}^n} \left[(C, \mathcal{C}_n(C)) \in R_{U_n^{\mathsf{Telep}}} \right] > \frac{35}{36} \ .$$

Then the circuit depth of $\mathcal{C}_n$ satisfies

$$\mathsf{depth}(\mathcal{C}_n) \in \Omega(\log n) \ .$$

We will refer to constant $\nu := 35/36$ in Theorem 3.1 as the classical soundness parameter. In Section 7 (see Theorem 7.3), we use parallel repetition to show ν can be replaced by a constant arbitrarily close to 0 by modifying the relation.

An immediate implication of Theorem 3.1 is the following corollary. It constitutes an extension of the worst-case result proven in Ref. [9] to the average case. We note, however, that [9] uses different proof techniques, relying on a reduction to circuit-complexity lower bounds for computing parity. In more detail, the work [9] considers a particular subset of instances and shows that a circuit succeeding on this subset implies a constant-depth circuit computing parity, a contradiction. For Theorem 3.1 and Corollary 3.2, we consider the uniform distribution over all instances (i.e., n-tuples of Cliffords) instead. This is essential to be able to apply random restriction techniques (see Section 4), as random restrictions are generally not compatible with specific constraints on the set of considered instances.

Corollary 3.2. *No* NC^0 *circuit can solve the relation problem* $R_{U_n^{\mathsf{Telep}}}$ *of possibilistic simulation of gate teleportation for a uniformly chosen instance with average probability greater than* $35/36$*. In particular, the relation problem* $R_{U_n^{\mathsf{Telep}}}$ *separates the (relational) complexity classes* NC^0 *and* QNC^0 *of constant-depth classical and constant-depth quantum circuits, i.e., we have* $\mathsf{QNC}^0 \not\subseteq \mathsf{NC}^0$*.*

Theorem 3.1 is a consequence of a no-signaling argument similar in spirit to those used in [7, 6]. The main idea is to show that any classical, limited-depth circuit achieving an average success probability above some threshold value implies a local strategy for a certain generalization of the magic-square game captured in Lemma 1.3.

Our proof relies on non-signaling properties of NC^0 circuits. Let us formalize the non-signaling property. A circuit $\mathcal{C}_n : \mathsf{Cliff}^n \to \mathsf{Pauli}^n$ as considered in this section has n inputs, where the r-th input C_r takes a single-qubit Clifford $C_r \in \mathsf{Cliff}$. Similarly, there are n outputs, with the s-th output labeled as P_s (outputting a Pauli $P_s \in \mathsf{Pauli}$)[1]. Given an input C_r, $r \in \mathbb{Z}_n$ to the circuit, we define the forward light cone $\mathcal{L}^{\rightarrow}_{\mathcal{C}_n}(C_r)$ of C_r as the set of outputs whose associated output depends non-trivially on the input C_r. That is, the set $\mathcal{L}^{\rightarrow}_{\mathcal{C}_n}(C_r)$ contains every output P_s, $s \in \mathbb{Z}_n$ with the following property: There are two n-tuples $C, C' \in \mathsf{Cliff}^n$ of Cliffords such that C and C' agree in each entry $m \in \mathbb{Z}_n \backslash \{r\}$, yet lead to two outputs $\mathcal{C}_n(C), \mathcal{C}_n(C') \in \mathsf{Pauli}^n$ differing in the entry $s \in \mathbb{Z}_n$, i.e., $\mathcal{C}_n(C)_s \neq \mathcal{C}_n(C')_s$. Similarly, we can define the backward light cone $\mathcal{L}^{\leftarrow}_{\mathcal{C}_n}(P_s)$ of output P_s as the set of inputs that non-trivially affect output P_s. That is, the set $\mathcal{L}^{\leftarrow}_{\mathcal{C}_n}(P_s)$ contains every input $C_r, r \in \mathbb{Z}_n$ such that $P_s \in \mathcal{L}^{\rightarrow}_{\mathcal{C}_n}(C_r)$.

The relevant no-signaling property can now be concisely stated in these terms, as follows. We say that inputs C_j and C_k have non-intersecting light cones or are non-signaling if and only if

$$\mathcal{L}^{\rightarrow}_{\mathcal{C}_n}(C_j) \cap \mathcal{L}^{\rightarrow}_{\mathcal{C}_n}(C_k) = \emptyset \ .$$

This property implies that the output Paulis can be partitioned into three disjoint sets namely:

(i) output Paulis that depend on C_j (indexed by $P_s \in \mathcal{L}^{\rightarrow}_{\mathcal{C}_n}(C_j)$),

(ii) output Paulis that depend on C_k (indexed by $P_s \in \mathcal{L}^{\rightarrow}_{\mathcal{C}_n}(C_k)$), and

(iii) output Paulis that do not depend on either C_j or C_k (indexed by $P_s \in \left(\mathcal{L}^{\rightarrow}_{\mathcal{C}_n}(C_j) \cup \mathcal{L}^{\rightarrow}_{\mathcal{C}_n}(C_k)\right)^c$).

(Here A^c denotes the complement of a subset $A \subset \mathbb{Z}_n$.) Importantly, there are no output entries depending simultaneously on both (C_j, C_k) if the inputs C_j and C_k are non-signaling.

The following statement shows that any limited-depth circuit with bounded fan-in gates has at least two inputs C_j, C_k that are non-signaling. We refer to [6] for a proof, where an essentially analogous setup is considered.

Lemma 3.3 ([6, Lemma 7])**.** *There is a constant* $c > 0$ *such that the following holds. Let* $\mathcal{C}_n : \mathsf{Cliff}^n \to \mathsf{Pauli}^n$ *be a circuit with gates of fan-in bounded by a constant* K*. If*

$$\mathsf{depth}(\mathcal{C}_n) \leq c \frac{\log n}{\log K} \ ,$$

then there are two inputs C_j, C_k *with* $(j < k)$ *that are non-signaling.*

[1] For convenience, we will slightly abuse the notation and use inputs and outputs for the input nodes and output nodes of the circuit.

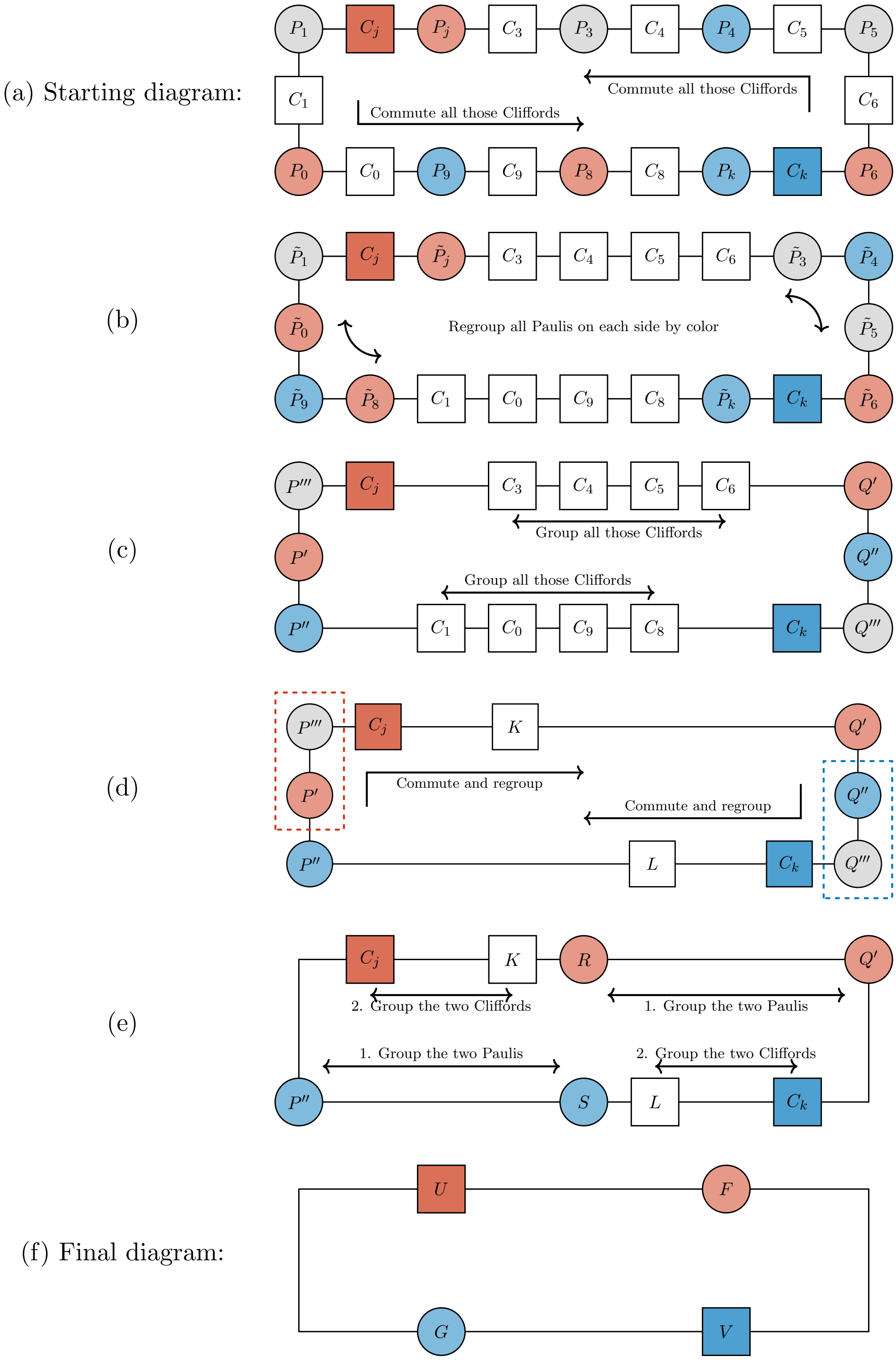

Supplementary Figure 7: Tensor-network representation illustrating the various commutation relations used in the proof of Theorem 3.1. We give an example of dimension $n = 10$, with $j = 2$ and $k = 7$. The input Cliffords are represented by squares, while the output Paulis are represented by circles of various colours: (i) the Paulis in red belong to the forward light cone of the Clifford C_j in red. (ii) the Paulis in blue belong to the forward light cone of the Clifford C_k in blue. (iii) The Paulis in grey are in neither of those light cones.

Proof of Theorem 3.1. Supplementary Figure 7 illustrates the main steps of the proof. We first rewrite the condition $(C,P) \in R_{U_n^{\mathsf{Telep}}}$ in a form that is convenient for our analysis. For this purpose, let $j,k \in \mathbb{Z}_n$ with $j < k$ be arbitrary but fixed. Observe that $(C,P) \in R_{U_n^{\mathsf{Telep}}}$ if and only if

$$A := \left|\mathrm{tr}\left(\left(\prod_{r=k+1}^{n-1} P_r C_r\right) P_k C_k \left(\prod_{s=j+1}^{k-1} P_s C_s\right) P_j C_j \left(\prod_{t=0}^{j-1} P_t C_t\right)\right)\right| > 0 \ . \tag{9}$$

Using the cyclicity of trace, we can rewrite the quantity A as

$$A = \left|\mathrm{tr}\left(C_j \left(\prod_{s\in[k+1,\ldots,n-1,0,1,\ldots,j-1]} P_s C_s\right) P_k C_k \left(\prod_{t\in[j+1,\ldots,k-1]} P_t C_t\right) P_j\right)\right| \ , \tag{10}$$

where we used the product indexed by elements of $\mathbb{Z}_n$:

$$\prod_{s\in[k+1,\ldots,n-1,0,1,\ldots,j-1]} P_s C_s = (P_{j-1}C_{j-1})\cdots(P_{n-1}C_{n-1})(P_0C_0)(P_1C_1)\cdots(P_{k+1}C_{k+1})$$
$$\prod_{t\in[j+1,\ldots,k-1]} P_t C_t = (P_{k-1}C_{k-1})\cdots(P_{j+1}C_{j+1}) \ .$$

We will simply write $[k+1,\ldots,j-1]$ instead of $[k+1,\ldots,n-1,0,1,\ldots,j-1]$ when using the product indexed by elements of $\mathbb{Z}_n$.

We now use the fact that Clifford group normalizes the Pauli group. This allows us to commute all Paulis in the two products in Eq. (10) to the right. That is, we define Pauli

$$\tilde{P}_s := \left(\prod_{t\in[k+1,\ldots,s]} C_t\right)^\dagger P_s \left(\prod_{r\in[k+1,\ldots,s]} C_r\right) \qquad \text{for all} \qquad s \in [k+1,\ldots,j-1] \tag{11}$$

$$\tilde{P}_t := \left(\prod_{t\in[j+1,\ldots,s]} C_t\right)^\dagger P_s \left(\prod_{r\in[j+1,\ldots,s]} C_r\right) \qquad \text{for all} \qquad t \in [j+1,\ldots,k-1]$$

$$\tilde{P}_j := P_j$$
$$\tilde{P}_k := P_k \tag{12}$$

such that

$$\prod_{s\in[k+1,\ldots,j-1]} P_s C_s = \left(\prod_{u\in[k+1,\ldots,j-1]} C_u\right)\left(\prod_{v\in[k+1,\ldots,j-1]} \tilde{P}_v\right) \tag{13}$$

$$\prod_{t\in[j+1,\ldots,k-1]} P_t C_t = \left(\prod_{u\in[j+1,\ldots,k-1]} C_u\right)\left(\prod_{v\in[j+1,\ldots,k-1]} \tilde{P}_v\right) \ . \tag{14}$$

Substituting (13) and (14) into Eq. (10), we obtain the necessary and sufficient condition

$$A = \left|\mathrm{tr}\left(\left(\prod_{u\in[k+1,\ldots,j]} C_u\right)\left(\prod_{s\in[k,\ldots,j-1]} \tilde{P}_s\right)\left(\prod_{u\in[j+1,\ldots,k]} C_u\right)\left(\prod_{t\in[j,\ldots,k-1]} \tilde{P}_t\right)\right)\right| > 0 \tag{15}$$

for $(C,P) \in R_{U_n^{\mathsf{Telep}}}$. For later use, we note that each $\tilde{P}_s$, $s \in \mathbb{Z}_n$ defined by Eqs. (11)– (12) is obtained by commuting Paulis past Clifford operators belonging to the $(n-2)$-tuple

$$C^{\mathrm{c}}_{j,k} := (C_0,\ldots,C_{j-1},C_{j+1},\ldots,C_{k-1},C_{k+1},\ldots,C_{n-1}) \ , \tag{16}$$

that is, we have

$$\tilde{P}_s = \tilde{P}_s(P_s, C^c_{j,k}) \tag{17}$$

for a certain function $\tilde{P}_s$, for every $s \in \mathbb{Z}_n$. In particular, given P_s, $\tilde{P}_s$ is determined by (16) and there is no dependence on (C_j, C_k).

To prove Theorem 3.1, suppose for the sake of contradiction that there is a circuit $\mathcal{C}_n$ with gates of bounded fan-in K satisfying

$$\mathsf{depth}(\mathcal{C}_n) \leq c\frac{\log n}{\log K} ,$$

where c is the constant from Lemma 3.3, and achieving an average success probability

$$\Pr_{C \in \mathsf{Cliff}^n}\left[(C, \mathcal{C}_n(C)) \in R_{U_n^{\mathsf{Telep}}}\right] > \frac{35}{36} . \tag{18}$$

By Lemma 3.3, there are $j < k$ such that

$$\mathcal{L}^{\rightarrow}_{\mathcal{C}_n}(C_j) \cap \mathcal{L}^{\rightarrow}_{\mathcal{C}_n}(C_k) = \emptyset . \tag{19}$$

As discussed before Lemma 4.2, this means that for every $s \in \mathbb{Z}_n$, the outputs have the following property: Either

(i) $P_s \in \mathcal{L}^{\rightarrow}_{\mathcal{C}_n}(C_j)$,

(ii) $P_s \in \mathcal{L}^{\rightarrow}_{\mathcal{C}_n}(C_k)$, or

(iii) $P_s \notin \left(\mathcal{L}^{\rightarrow}_{\mathcal{C}_n}(C_j) \cup \mathcal{L}^{\rightarrow}_{\mathcal{C}_n}(C_k)\right)$.

This implies the following:

(I) In case (i), the Pauli P_s depends non-trivially on C_j, i.e., there is a function $P_s : \mathsf{Cliff} \times (\mathsf{Cliff})^{n-2} \to \mathsf{Pauli}$ and write $P_s(C_j, C^c_{j,k})$.

Combined with Eq. (17), this implies that there is a function $\tilde{P}_s : \mathsf{Cliff} \times (\mathsf{Cliff})^{n-2} \to \mathsf{Pauli}$ such that $\tilde{P}_s = \tilde{P}_s(C_j, C^c_{j,k})$.

(II) In case (ii), the Pauli P_s depends non-trivially on the input C_k, i.e., there is a function $P_s : \mathsf{Cliff} \times (\mathsf{Cliff})^{n-2} \to \mathsf{Pauli}$ such that $P_s = P_s(C_k, C^c_{j,k})$.

Combined with Eq. (17), this implies that there is a function $\tilde{P}_s : \mathsf{Cliff} \times (\mathsf{Cliff})^{n-2} \to \mathsf{Pauli}$ such that $\tilde{P}_s = \tilde{P}_s(C_k, C^c_{j,k})$.

(III) In case (iii), the Pauli P_s depends only on $C^c_{j,k}$, i.e., there is a function $P_s : (\mathsf{Cliff})^{n-2} \to \mathsf{Pauli}$ such that $P_s = P_s(C^c_{j,k})$.

Because of Eq. (17), there is a function $\tilde{P}_s : (\mathsf{Cliff})^{n-2} \to \mathsf{Pauli}$ such that $\tilde{P}_s = \tilde{P}_s(C^c_{j,k})$.

Now consider the products of Pauli operators appearing in Eq. (15). We can reorder these according to the three cases (I)–(III) above since Pauli operators either commute or anticommute. This gives factorizations of the form

$$\prod_{t \in [j,\ldots,k-1]} \tilde{P}_t(C) = \pm P''_{C^c_{j,k}}(C_k) P'_{C^c_{j,k}}(C_j) P'''_{C^c_{j,k}} \quad \text{where} \quad \begin{array}{rcl} P'_{C^c_{j,k}}(C_j) & := & \prod_{t \in [j,\ldots,k-1] \cap \mathcal{L}^{\rightarrow}_{\mathcal{C}_n}(C_j)} \tilde{P}_t(C_j, C^c_{j,k}) \\ P''_{C^c_{j,k}}(C_k) & := & \prod_{t \in [j,\ldots,k-1] \cap \mathcal{L}^{\rightarrow}_{\mathcal{C}_n}(C_k)} \tilde{P}_t(C_k, C^c_{j,k}) \\ P'''_{C^c_{j,k}} & := & \prod_{t \in [j,\ldots,k-1] \setminus (\mathcal{L}^{\rightarrow}_{\mathcal{C}_n}(C_j) \cup \mathcal{L}^{\rightarrow}_{\mathcal{C}_n}(C_k))} \tilde{P}_t(C^c_{j,k}) \end{array}$$

and

$$\prod_{s\in[k,\ldots,j-1]} \tilde{P}_s(C) = \pm Q'_{C^c_{j,k}}(C_j) Q''_{C^c_{j,k}}(C_k) Q'''_{C^c_{j,k}} \text{ where } \begin{array}{lll} Q'_{C^c_{j,k}}(C_j) & := & \prod_{s\in[k,\ldots,j-1]\cap\mathcal{L}^{\rightarrow}_{\mathcal{C}_n}(C_j)} \tilde{P}_s(C_j, C^c_{j,k}) \\ Q''_{C^c_{j,k}}(C_k) & := & \prod_{s\in[k,\ldots,j-1]\cap\mathcal{L}^{\rightarrow}_{\mathcal{C}_n}(C_k)} \tilde{P}_s(C_k, C^c_{j,k}) \\ Q'''_{C^c_{j,k}} & := & \prod_{s\in[k,\ldots,j-1]\setminus(\mathcal{L}^{\rightarrow}_{\mathcal{C}_n}(C_j)\cup\mathcal{L}^{\rightarrow}_{\mathcal{C}_n}(C_k))} \tilde{P}_s(C^c_{j,k}) \end{array} ,$$

where we choose a slightly different order of the three factors in the two products for convenience only. Inserting this into (15), and using curly brackets to emphasize associativity we obtain

$$\begin{aligned} A &= \left| \mathrm{tr}\left(\left(\left(\prod_{u\in[k+1,\ldots,j]} C_u \right) \left(\prod_{s\in[k,\ldots,j-1]} \tilde{P}_s \right) \left(\prod_{u\in[j+1,\ldots,k]} C_u \right) \left(\prod_{t\in[j,\ldots,k-1]} \tilde{P}_t \right) \right) \right) \right| \\ &= \left| \mathrm{tr}\left(\left\{ C_j K_{C^c_{j,k}} \right\} Q'_{C^c_{j,k}}(C_j) Q''_{C^c_{j,k}}(C_k) Q'''_{C^c_{j,k}} \left\{ C_k L_{C^c_{j,k}} \right\} P''_{C^c_{j,k}}(C_k) P'_{C^c_{j,k}}(C_j) P'''_{C^c_{j,k}} \right) \right| , \end{aligned} \quad (20)$$

where we defined the Clifford operators

$$K_{C^c_{j,k}} := \prod_{u\in[k+1,\ldots,j-1]} C_u \qquad \text{and} \qquad L_{C^c_{j,k}} := \prod_{u\in[j+1,\ldots,k-1]} C_u \ .$$

In the following, we will temporarily suppress the dependence on the tuple $C^c_{j,k}$, omitting this subscript. Again using the fact that Paulis commute or anticommute and the cyclicity of the trace, we rearrange (20) by moving all factors depending on C_j and C_k, respectively, next to each other. We obtain

$$\begin{aligned} A &= \left| \mathrm{tr}\left(\{C_j K\} Q'(C_j) Q''(C_k) Q''' \{C_k L\} P''(C_k) \left\{ P'(C_j) P''' \right\} \right) \right| \\ &= \left| \mathrm{tr}\left(\left\{ P'(C_j) P''' \right\} \{C_j K\} Q'(C_j) Q''(C_k) Q''' \{C_k L\} P''(C_k) \right) \right| \quad \text{by the cyclicity of the trace} \\ &= \left| \mathrm{tr}\left(\left\{ P'(C_j) P''' \right\} \{C_j K\} Q'(C_j) \left\{ Q''(C_k) Q''' \right\} \{C_k L\} P''(C_k) \right) \right| \\ &= \left| \mathrm{tr}\left(\{C_j K\} R(C_j) Q'(C_j) \{C_k L\} S(C_k) P''(C_k) \right) \right| \end{aligned} \quad (21)$$

where we introduced the Paulis

$$\begin{aligned} R(C_j) &= \{C_j K\}^\dagger \left\{ P'(C_j) P''' \right\} \{C_j K\} \\ S(C_k) &= \{C_k L\}^\dagger \left\{ Q''(C_k) Q''' \right\} \{C_k L\} \end{aligned}$$

by commuting $P'(C_j)P'''$ to the right of the factor C_jK, and $Q''(C_k)Q'''$ to the right of the factor C_jL. Finally, we can use associativity and define Pauli operators $\tilde{F}$ and $\tilde{G}$ by Eq. (21) as follows

$$\begin{aligned} A &= \left| \mathrm{tr}\left(C_j K \underbrace{R(C_j) Q'(C_j)}_{=:\tilde{F}} C_k L \underbrace{S(C_k) P''(C_k)}_{=:\tilde{G}} \right) \right| \\ &= \left| \mathrm{tr}\left(C_j K \tilde{F} C_k L \tilde{G} \right) \right| \end{aligned}$$

Let us briefly summarize what we have obtained recalling the different dependencies. We have, for each $(n-2)$ tuple $C^c_{j,k} \in \mathsf{Cliff}^{n-2}$, two functions

$$\begin{array}{rccc} \tilde{F}_{C^c_{j,k}} : & \mathsf{Cliff} & \to & \mathsf{Pauli} \\ & C_j & \mapsto & \pm R_{C^c_{j,k}}(C_j) Q'_{C^c_{j,k}}(C_j) \end{array}$$

and

$$\begin{array}{rccc} \tilde{G}_{C^c_{j,k}} : & \mathsf{Cliff} & \to & \mathsf{Pauli} \\ & C_k & \mapsto & \pm S_{C^c_{j,k}}(C_k) P''_{C^c_{j,k}}(C_k) \end{array}$$

where the signs are chosen appropriately such that the expression belongs to Pauli, as well as Clifford operators $K_{C^c_{j,k}}, L_{C^c_{j,k}} \in \mathsf{Cliff}$ such that $(C, \mathcal{C}_n(C)) \in R_{U_n^{\mathsf{Telep}}}$ if and only if

$$\left|\mathrm{tr}\left(C_j K_{C^c_{j,k}} \tilde{F}_{C^c_{j,k}}(C_j) C_k L_{C^c_{j,k}} \tilde{G}_{C^c_{j,k}}(C_k)\right)\right| > 0 \ . \tag{22}$$

Now consider the probability $p_{|C^c_{j,k}}$ that, conditioned on a fixed choice of $C^c_{j,k} \in \mathsf{Cliff}^{n-2}$, the circuit $\mathcal{C}_n$ produces a valid solution to a randomly chosen instance from the set of n-tuples of Cliffords wich coincide with $C^c_{j,k}$ in entries $\ell \notin \{j,k\}$, and are arbitrary otherwise (i.e., in the entries j and k). By (22), the probability $p_{|C^c_{j,k}}$ is given by

$$\begin{aligned} p_{|C^c_{j,k}} &= \frac{1}{|\mathsf{Cliff}|^2}\left|\left\{(U,V) \in \mathsf{Cliff}^2 \ : \ \left|\mathrm{tr}\left(U K_{C^c_{j,k}} \tilde{F}_{C^c_{j,k}}(U) V L_{C^c_{j,k}} \tilde{G}_{C^c_{j,k}}(V)\right)\right| > 0\right\}\right| \\ &= \frac{1}{|\mathsf{Cliff}|^2}\left|\left\{(U,V) \in \mathsf{Cliff}^2 \ : \ \left|\mathrm{tr}\left(U \tilde{F}_{C^c_{j,k}}(U K^{-1}_{C^c_{j,k}}) V \tilde{G}_{C^c_{j,k}}(V L^{-1}_{C^c_{j,k}})\right)\right| > 0\right\}\right| \ , \end{aligned} \tag{23}$$

where the last line is obtained by variable substition.

By definition of $p_{|C^c_{j,k}}$ and assumption (18), we have

$$\mathbb{E}_{C^c_{j,k}}[p_{|C^c_{j,k}}] = \Pr_{C \in \mathsf{Cliff}^n}\left[(C, \mathcal{C}_n(C)) \in R_{U_n^{\mathsf{Telep}}}\right] > \frac{35}{36}$$

for a uniformly random choice of the $(n-2)$-tuple $C^c_{j,k} \in \mathsf{Cliff}^{n-2}$. This shows that there is at least one choice of $C^c_{j,k}$ such that $p_{|C^c_{j,k}} > \frac{35}{36}$. In particular, for such a choice, we obtain

$$\frac{1}{|\mathsf{Cliff}|^2}\left|\left\{(U,V) \in \mathsf{Cliff}^2 \mid \left|\mathrm{tr}\left(U \tilde{F}_{C^c_{j,k}}(U K^{-1}_{C^c_{j,k}}) V \tilde{G}_{C^c_{j,k}}(V L^{-1}_{C^c_{j,k}})\right)\right| > 0\right\}\right| > \frac{35}{36} \tag{24}$$

by Eq. (23). Defining the functions

$$\begin{array}{rccc} F: & \mathsf{Cliff} & \to & \mathsf{Pauli} \\ & U & \mapsto & \tilde{F}_{C^c_{j,k}}(U K^{-1}_{C^c_{j,k}}) \end{array} \quad \text{and} \quad \begin{array}{rccc} G: & \mathsf{Cliff} & \to & \mathsf{Pauli} \\ & V & \mapsto & \tilde{G}_{C^c_{j,k}}(V L^{-1}_{C^c_{j,k}}) \ , \end{array}$$

Eq. (24) becomes

$$\Pr_{(U,V) \in \mathsf{Cliff}^2}[|\mathrm{tr}(UF(U)VG(V)| > 0] > \frac{35}{36} \ .$$

The existence of two such functions F, G contradicts Lemma 1.3. This concludes the proof of the theorem. □

4 Towards proving an advantage against AC^0: variants of the quantum advantage against NC^0

In this section, we prove a few auxiliary results extending the results for NC^0-circuits from the previous section. These are needed to show the AC^0-circuit size lower bound we prove by restriction based methods [24, 3] in Section 5. In particular, to prepare for the proof that the possibilistic simulation of the gate-teleportation circuit is not solvable by an AC^0 circuit of polynomial size, we show here that the gate-teleportation problem cannot be solved by an NC^0 circuit even when some of the inputs of the NC^0 circuit are fixed.

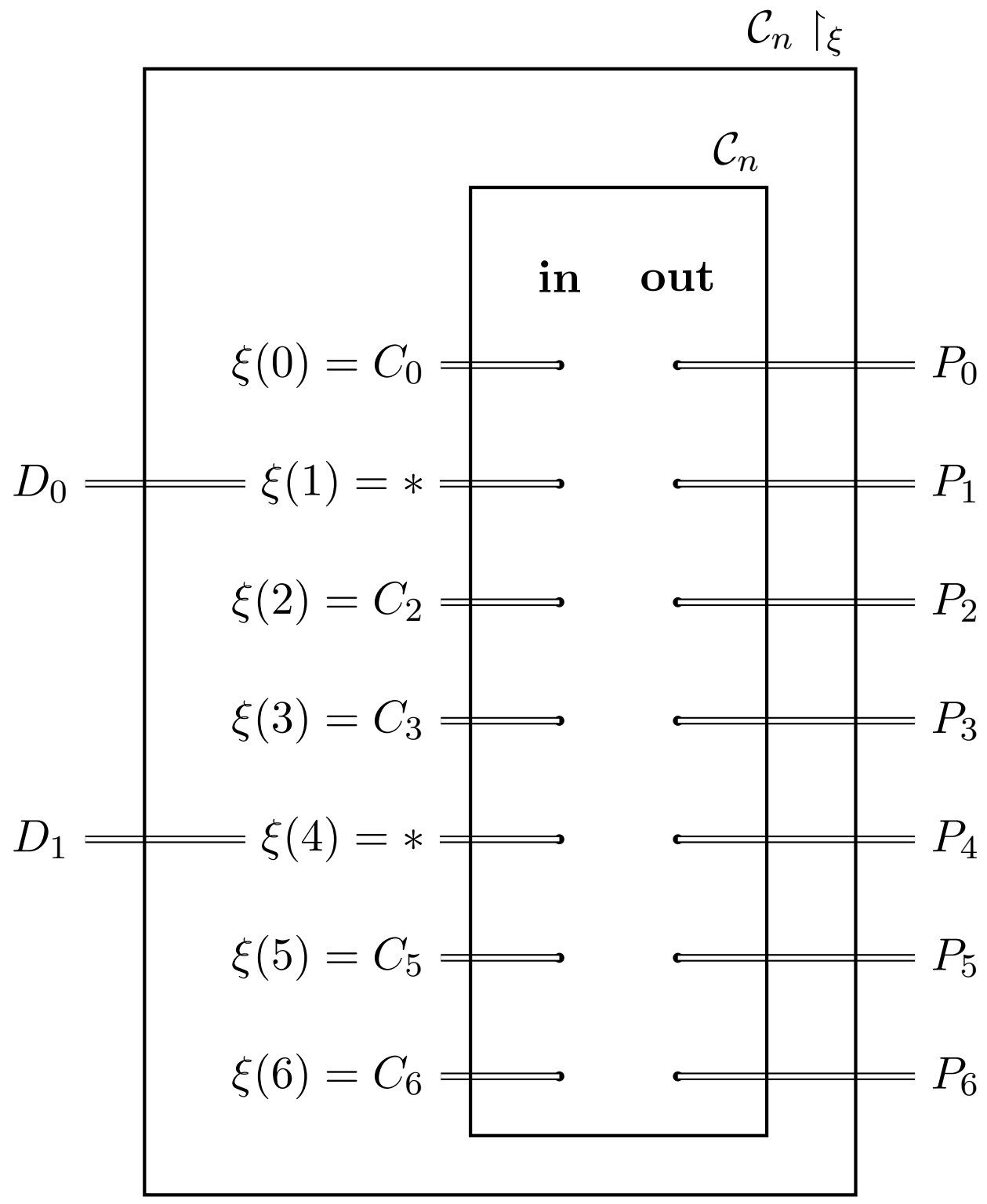

Supplementary Figure 8: Schematic representation of the effect of a restriction $\xi : \mathbb{Z}_n \to \mathsf{Cliff} \cup \{*\}$ on the circuit $\mathcal{C}_n$. The inputs C_i are fixed while the inputs D_i are active; it gives rise to the restricted circuit $\mathcal{C}_n \restriction_\xi$.

4.1 Average-case hardness of $R_{U_n^{\mathsf{Telep}}} \restriction_\xi$ for NC^0-circuit

The first strengthening of the result for NC^0-circuits from the previous section concerns the problem of possibilistically simulating gate-teleportation with some fixed inputs. To formalize this, we define a restriction of n Clifford inputs as a function $\xi : \mathbb{Z}_n \to \mathsf{Cliff} \cup \{*\}$ which fixes some Clifford elements and leaves the remaining inputs marked by "$*$" active (or free). Let $m = |\xi^{-1}(\{*\})|$ be the number of active inputs. We use $\xi^{-1}(\{*\}) = \{\iota(0) < \iota(1) < \ldots < \iota(m-1)\} \subset \mathbb{Z}_n$ to denote the active inputs. The application of restriction ξ to a circuit $\mathcal{C}$ is defined as

$$\begin{array}{cccc} \mathcal{C}_n \restriction_\xi : & \mathsf{Cliff}^m & \to & \mathsf{Pauli}^n \\ & (x_0, \ldots, x_{m-1}) & \mapsto & \mathcal{C}(x'_0, \ldots, x'_{n-1}) \ , \end{array}$$

where for $j \in \mathbb{Z}_m$

$$x'_j = \begin{cases} x_{\iota^{-1}(j)} & \text{if } \xi(j) = * \\ \xi(j) & \text{otherwise} \ . \end{cases}$$

See Supplementary Figure 8 for an illustration of a restriction applied to a classical circuit for the gate-teleportation problem. Here we define a restriction of Clifford inputs, but restrictions can be naturally defined on any type of inputs. In Section 4.3 and Section 5.2 we will use restrictions that fixes bits and blocks of bits, i.e., strings.

We will use restriction to define simulation problem with some inputs fixed. Let $\xi : \mathbb{Z}_n \to \mathsf{Cliff} \cup \{*\}$ be a restriction. We define the restricted problem of possibilistic simulation of gate-teleportation $R_{U_n^{\mathsf{Telep}}} \restriction_\xi \subset \mathsf{Cliff}^m \times \mathsf{Pauli}^n$ as the possibilistic simulation of the quantum circuit

$U_n^{\mathsf{Telep}} \restriction_\xi$. Formally, let $D = (D_0, \ldots, D_{m-1}) \in \mathsf{Cliff}^m$ and let $P = (P_0, \ldots, P_{n-1}) \in \mathsf{Pauli}^n$, then

$$(D, P) \in R_{U_n^{\mathsf{Telep}}} \restriction_\xi \qquad \text{if and only if} \qquad (C, P) \in R_{U_n^{\mathsf{Telep}}} ,$$

where $C = (C_0, \ldots, C_{n-1})$ is such that for all $\ell \in \mathbb{Z}_n$

$$C_\ell = \begin{cases} \xi(\ell) & \text{if} \qquad \xi(\ell) \neq * \\ D_{\iota^{-1}(\ell)} & \text{otherwise} \quad . \end{cases}$$

We extend the bound for NC^0-circuits for the problem $R_{U_n^{\mathsf{Telep}}}$ given in Theorem 3.1 to the restricted gate-teleportation problem $R_{U_n^{\mathsf{Telep}}} \restriction_\xi$ with some inputs fixed.

Theorem 4.1. *Let $0 < \gamma \leq 1$ and $1/2 < \delta \leq 1$ be constants. Let $\xi : \mathbb{Z}_n \to \mathsf{Cliff} \cup \{*\}$ be a restriction that keeps $m \geq \gamma n^\delta$ inputs active. Then any NC^0-circuit $\mathcal{C}_n : \mathsf{Cliff}^m \to \mathsf{Pauli}^n$ solves the restricted gate-teleportation problem $R_{U_n^{\mathsf{Telep}}} \restriction_\xi$ with average probability at most*

$$\Pr_{D \in \mathsf{Cliff}^m} \left[(D, \mathcal{C}_n(D)) \in R_{U_n^{\mathsf{Telep}}} \restriction_\xi \right] < \frac{35}{36}$$

for an instance D chosen uniformly from the set Cliff^m.

We refer to Theorem 7.4 in Section 7 for a variant of this result (obtained by parallel repetition) where the classical soundness parameter $\nu := 35/36$ is replaced by a constant arbitrarily close to 0.

The proof of this theorem follows the proof of Theorem 3.1 and also relies on a light-cone argument. There is one important difference we need to consider. The NC^0-circuit solving the restricted problem has an imbalanced number of inputs and outputs. We show that if such an imbalance is "reasonable" the light-cone argument leading to non-signaling inputs still holds. We capture this in the following lemma.

Lemma 4.2. *Let $0 < \gamma \leq 1$ and $1/2 < \delta \leq 1$. Let $\mathcal{C}_n$ be an NC^0-circuit with constant depth d and bounded fan-in K, and with $m \geq \gamma n^\delta$ inputs and n outputs (the inputs and the outputs can be bits or have higher alphabet size). Then there exist two inputs $j, k \in \mathbb{Z}_n$ with non-intersecting light cones, i.e.,*

$$\mathcal{L}^{\rightarrow}_{\mathcal{C}_n}(j) \cap \mathcal{L}^{\rightarrow}_{\mathcal{C}_n}(k) = \emptyset \ .$$

Proof. We first bound the expected number of outputs in the forward light cone of a randomly chosen input. Let the locality ℓ be defined as $\ell := K^d$ where $d := \mathsf{depth}(\mathcal{C}_n)$ is the depth of $\mathcal{C}_n$. Notice that the locality is the maximum number of inputs that can be in a backward light cone $\mathcal{L}^{\leftarrow}_{\mathcal{C}_n}(o)$ of any output $o \in \mathbb{Z}_n$ that is

$$|\mathcal{L}^{\leftarrow}_{\mathcal{C}_n}(o)| \leq \ell \qquad \text{for all} \qquad o \in \mathbb{Z}_n \ . \tag{25}$$

Consider a bipartite graph $G = (V_I \cup V_O, E)$, where the set of vertices $V_I = \mathbb{Z}_m$ is the set of inputs and the set $V_O = \mathbb{Z}_n$ of outputs. There is an edge connecting vertices v_i and v_o whenever $v_o \in \mathcal{L}^{\rightarrow}(v_i)$. By (25) the total number of edges is bounded by

$$|E| \leq \ell \cdot n \ .$$

This allows us to bound the expected degree of an input node $j \in V_I$ picked uniformly at random. By definition of G this is the expected size of the forward light cone of an input j picked uniformly at random, i.e.,

$$\mathop{\mathbb{E}}_j [|\mathcal{L}^{\rightarrow}(j)|] = \mathop{\mathbb{E}}_i [\deg(v_i)] \leq \ell n^{1-\delta} \gamma^{-1} \ , \tag{26}$$

where we used that $m = |V_I| \geq \gamma n^\delta$ by assumption. From Eq. (26), we conclude that there exists at least one input $j \in V_I$ for which

$$|\mathcal{L}^{\rightarrow}(j)| \leq \ell n^{1-\delta}\gamma^{-1} \ .$$

We note that for any output o, $|\mathcal{L}^{\leftarrow}(o)| \leq \ell$, and that therefore for the previously mentioned input j we have

$$|\mathcal{L}^{\leftarrow}(\mathcal{L}^{\rightarrow}(j))| \leq \ell^2 n^{1-\delta}\gamma^{-1} \ .$$

We conclude the proof by picking any element k from the set

$$S = \{k \in V_I : \mathcal{L}^{\rightarrow}_{\mathcal{C}_n}(j) \cap \mathcal{L}^{\rightarrow}_{\mathcal{C}_n}(k) = \emptyset\} \ ,$$

and by remarking that it is non-empty for sufficiently large n because

$$|S| \geq \gamma n^\delta - \ell^2 n^{1-\delta}\gamma^{-1}$$

since $\delta > 1/2$ by the assumption. □

Proof of Theorem 4.1. The argument is almost the same as in the proof of Theorem 3.1, but with every input instance having some of the Clifford inputs fixed. By definition of $R_{U_n^{\mathsf{Telep}}}\restriction_\xi$ for any instance $D \in \mathsf{Cliff}^m$ there is $C = (C_0, \ldots, C_{n-1}) \in \mathsf{Cliff}^n$ where for $\ell \in \mathbb{Z}_n$

$$C_\ell = \begin{cases} \xi(\ell) & \text{if} \quad \xi(\ell) \neq * \\ D_{\iota^{-1}(\ell)} & \text{otherwise} \end{cases} ,$$

such that $(D, \mathcal{C}_n(D)) \in R_{U_n^{\mathsf{Telep}}}\restriction_\xi$ if and only if $(C, \mathcal{C}_n(D)) \in R_{U_n^{\mathsf{Telep}}}$.

Let $\xi^{-1}(\{*\}) = \{\iota(0) < \iota(1) < \ldots < \iota(m-1)\}$ be the active inputs of the restriction ξ. Assume $j' < k' \in \mathbb{Z}_m$ are arbitrary but fixed and define $j := \iota(j')$ and $k := \iota(k')$. In the proof of Theorem 3.1 (Eqs. (9) – (15)) we derived a necessary and sufficient condition for $P \in \mathsf{Pauli}^n$ to be the solution of $R_{U_n^{\mathsf{Telep}}}$ problem for instance C. Following this argument and using the definition of $R_{U_n^{\mathsf{Telep}}}\restriction_\xi$ for an instance D we similarly obtain

$$\left| \mathrm{tr}\left(\left(\prod_{u\in[k+1,\ldots,j]} C_u \right) \left(\prod_{s\in[k,\ldots,j-1]} \tilde{P}_s \right) \left(\prod_{u\in[j+1,\ldots,k]} C_u \right) \left(\prod_{t\in[j,\ldots,k-1]} \tilde{P}_t \right) \right) \right| > 0 \ ,$$

where $\tilde{P}_s$ and $\tilde{P}_t$ are defined in Eq. (11)–(12).

For the sake of contradiction, assume there is an NC^0 circuit $\mathcal{C}_n$ satisfying

$$\Pr_{D\in\mathsf{Cliff}^m}\left[(D, \mathcal{C}_n(D)) \in R_{U_n^{\mathsf{Telep}}}\restriction_\xi\right] \geq \frac{35}{36} \ .$$

By Lemma 4.2 we have that any NC^0 circuit $\mathcal{C}_n$ with at least $m \geq \gamma n^\delta$ inputs, where $0 < \gamma \leq 1$ and $1/2 < \delta \leq 1$, and n outputs necessarily has two inputs $D_{j'}, D_{k'}$ for $j', k' \in \mathbb{Z}_m$ with the non-signalling property. By definition, these correspond to C_j and C_k in the instance C of the $R_{U_n^{\mathsf{Telep}}}$ problem. By exactly following the light-cone argument below (19) in the proof of Theorem 3.1, we obtain the same contradiction with Lemma 1.3. □

4.2 From Clifford inputs to input strings

To establish our results for AC^0, it is more convenient to work with circuits taking bitstrings as input. Since $|\mathsf{Cliff}| = 24$, there is an injective map $\iota : \mathsf{Cliff} \to \{0,1\}^5$ that we fix throughout the following. Consider a map $\mathsf{Enc} : \{0,1\}^5 \to \mathsf{Cliff}$ which satisfies

$$\begin{aligned} \mathsf{Enc}: \quad \{0,1\}^5 \quad &\to \quad \mathsf{Cliff} \\ x \quad &\mapsto \quad \mathsf{Enc}(x) := \begin{cases} \iota^{-1}(x) & \text{if } x \in \iota(\mathsf{Cliff}) \\ C_x & \text{otherwise} \end{cases} \end{aligned} \tag{27}$$

for a family $\{C_x\}_{x\in\{0,1\}^5\setminus\iota(\mathsf{Cliff})} \subset \mathsf{Cliff}$ of Clifford group elements. We note such a map is surjective, and completely specified by ι and the family $\{C_x\}_{x\in\{0,1\}^5\setminus\iota(\mathsf{Cliff})}$. Correspondingly, we sometimes write $\mathsf{Enc} = \mathsf{Enc}_{\{C_x\}_{x\in\{0,1\}^5\setminus\iota(\mathsf{Cliff})}}$. We will denote by $\mathcal{E}$ the set of such maps (each one parametrized by the associated family of Clifford group elements), and call this the set of valid encodings.

In the following, we consider n-tuples $\mathsf{Enc} = (\mathsf{Enc}^{(0)}, \ldots, \mathsf{Enc}^{(n-1)}) \in \mathcal{E}^n$ of valid encoding maps. Such a tuple defines a map

$$\begin{aligned} \mathsf{Enc}: \qquad (\{0,1\}^5)^n \qquad &\to \qquad \mathsf{Cliff}^n \\ x = (x^{(0)}, \ldots, x^{(n-1)}) \quad &\mapsto \quad \Big(\mathsf{Enc}^{(0)}(x^{(0)}), \ldots, \mathsf{Enc}^{(n-1)}(x^{(n-1)})\Big) \ . \end{aligned}$$

which we again denote by Enc by a slight abuse of notation.

We can now show that bounds on the average success probability over uniformly random sequences of Cliffords and uniformly random bitstrings encoding Cliffords are related.

Lemma 4.3. *Let $\mathcal{R} \subset \mathsf{Cliff}^n \times \mathsf{Pauli}^n$ be a relation. Let $F : \mathsf{Cliff}^n \to \mathsf{Pauli}^n$ and $\epsilon > 0$ be arbitrary. Then there is an n-tuple $\mathsf{Enc} = (\mathsf{Enc}^{(0)}, \ldots, \mathsf{Enc}^{(n-1)}) \in \mathcal{E}^n$ of valid encoding maps such that the composition $F \circ \mathsf{Enc} : (\{0,1\}^5)^n \to \mathsf{Pauli}^n$ satisfies*

$$\Pr_{x\in(\{0,1\}^5)^n} [(\mathsf{Enc}(x), (F \circ \mathsf{Enc})(x)) \in \mathcal{R}] > (1-\epsilon) \Pr_{C\in\mathsf{Cliff}^n} [(C, F(C)) \in \mathcal{R}] \ .$$

Here $x \in (\{0,1\}^5)^n$ and $C \in \mathsf{Cliff}^n$, respectively, are chosen uniformly at random.

Proof. We define a distribution over the set $\mathcal{E}^n$ of random n-tuples Enc of valid encoding maps as follows. Consider random variables

$$\{C_x^{(j)}\}_{\substack{j\in\mathbb{Z}_n \\ x\in\{0,1\}^5\setminus\iota(\mathsf{Cliff})}}$$

obtained by picking $C_x^{(j)} \in \mathsf{Cliff}$ uniformly and independently at random for every $j \in \mathbb{Z}_n$ and every $x \notin \iota(\mathsf{Cliff})$. Then, for each $j \in \mathbb{Z}_n$, let $\mathcal{E}^{(j)} = \mathcal{E}_{\{C_x^{(j)}\}_{x\in\{0,1\}^5\setminus\iota(\mathsf{Cliff})}}$ be the valid encoding map defined by (27). Equivalently, this means that the n-tuple $\mathsf{Enc} = \{\mathsf{Enc}^{(j)}\}_{j\in\mathbb{Z}_n}$ is a random variable with uniform distribution on $\mathcal{E}^n$.

Now consider the probability taken over the choice of Enc and the choice of $x \in (\{0,1\}^5)^n$ (chosen independently and uniformly) that the composition $F \circ \mathsf{Enc}$ provides a valid solution to the relation problem $\mathcal{R}$ on instance $\mathsf{Enc}(x) \in \mathsf{Cliff}^n$. Because the induced distribution of the random variable $\mathsf{Enc}(x)$ is the uniform distribution on Cliff^n, it follows that

$$\Pr_{\substack{\mathsf{Enc}\in\mathcal{E} \\ x\in(\{0,1\}^5)^n}} [(\mathsf{Enc}(x), (F \circ \mathsf{Enc})(x)) \in \mathcal{R}] = \Pr_{C\in\mathsf{Cliff}^n} [(C, F(C)) \in \mathcal{R}] \ . \tag{28}$$

The claim then follows immediately from Markov's inequality: By (28) we have

$$\mathbb{E}_{\mathsf{Enc}}[p(\mathsf{Enc})] = \Pr_{C \in \mathsf{Cliff}^n} [(C, F(C)) \in \mathcal{R}] \ ,$$

where

$$p(\mathsf{Enc}) := \Pr_{x \in (\{0,1\}^5)^n} [(\mathsf{Enc}(x), (F \circ \mathsf{Enc})(x))) \in \mathcal{R}] \ .$$

Applying Markov's inequality $\Pr[X \geq a] \leq \frac{\mathbb{E}[X]}{a}$, $a > 0$ to the random variable $X := 1 - p(\mathsf{Enc})$ and using the abbreviation $q := \Pr_{C \in \mathsf{Cliff}^n} [(C, F(C)) \in \mathcal{R}]$ gives

$$\Pr_{\mathsf{Enc}} [1 - p(\mathsf{Enc}) \geq a] \leq \frac{1-q}{a} \ .$$

With the choice

$$a = 1 - (1-\varepsilon)q$$

we obtain

$$\Pr_{\mathsf{Enc}} [p(\mathsf{Enc}) \leq (1-\varepsilon)q] \leq \frac{1-q}{(1-q) + \varepsilon q} < 1$$

for any $\epsilon \in (0,1)$. This shows the existence of $\mathsf{Enc} \in \mathcal{E}^n$ with the desired property, that is, $p(\mathsf{Enc}) > (1-\varepsilon)q$. □

4.3 Average-case hardness of $R_{U_n^{\mathsf{Telep}}} \restriction_\xi$ with bit encoded input

In the previous section, we defined mappings between bitstrings and Cliffords and used blocks of 5 bits to encode sequences of Clifford gates. To show our AC^0 circuit size lower bound we rely on a restriction-based method which works with circuits having binary inputs and outputs. One of the building blocks is an extension of the NC^0-result for the restricted relation problem $R_{U_n^{\mathsf{Telep}}} \restriction_\xi$ to the setting of circuits with binary inputs and corresponding restrictions fixing individual bits (rather than Clifford group elements). This is what we provide in this section. Specifically, we encode each Clifford by a bitstring $\{0,1\}^n$ and consider restrictions of the form $\xi : \mathbb{Z}_n \to \{0,1\}^5 \cup \{*\}$. We will then define an associated relation problem $R_{U_n^{\mathsf{Telep}}} \restriction_{\mathsf{Enc}(\xi)}$.

To show a result for NC^0-circuits with bits (strings) as input we need a bound on the success probability of a bit encoded version of the generalized variant of the magic-square game with random Cliffords. The following is a bit-string encoded version of Lemma 1.3.

Lemma 4.4. *Let* $\mathsf{Enc}^{(1)}, \mathsf{Enc}^{(2)} \in \mathcal{E}$ *be valid encodings. Let* $F : \{0,1\}^5 \to \mathsf{Pauli}$ *and* $G : \{0,1\}^5 \to \mathsf{Pauli}$ *be arbitrary functions. Then*

$$\Pr_{(x^{(1)},x^{(2)}) \in (\{0,1\}^5)^2} \left[\mathrm{tr}\left(\mathsf{Enc}^{(1)}(x^{(1)})F(x^{(1)})\mathsf{Enc}^{(2)}(x^{(2)})G(x^{(2)})\right) = 0\right] \geq \frac{1}{64}$$

Proof. First observe that

$$\Pr_{(x^{(1)},x^{(2)}) \in (\{0,1\}^5)^2} [(x^{(1)}, x^{(2)}) \in (\iota(\mathsf{Cliff}))^2] = \frac{9}{16}$$

The bound then follows by applying Lemma 1.3: We have

$$\begin{aligned}
&\Pr_{(x^{(1)},x^{(2)}) \in (\{0,1\}^5)^2} \left[\mathrm{tr}\left(\mathsf{Enc}^{(1)}(x^{(1)})F(x^{(1)})\mathsf{Enc}^{(2)}(x^{(2)})G(x^{(2)})\right) = 0\right] \\
\geq &\Pr_{(x^{(1)},x^{(2)}) \in (\iota(\mathsf{Cliff}))^2} \left[\mathrm{tr}\left(\mathsf{Enc}^{(1)}(x^{(1)})F(x^{(1)})\mathsf{Enc}^{(2)}(x^{(2)})G(x^{(2)})\right) = 0\right] \cdot \frac{9}{16} \\
\geq &\frac{1}{36} \cdot \frac{9}{16} = \frac{1}{64} \ .
\end{aligned}$$

□

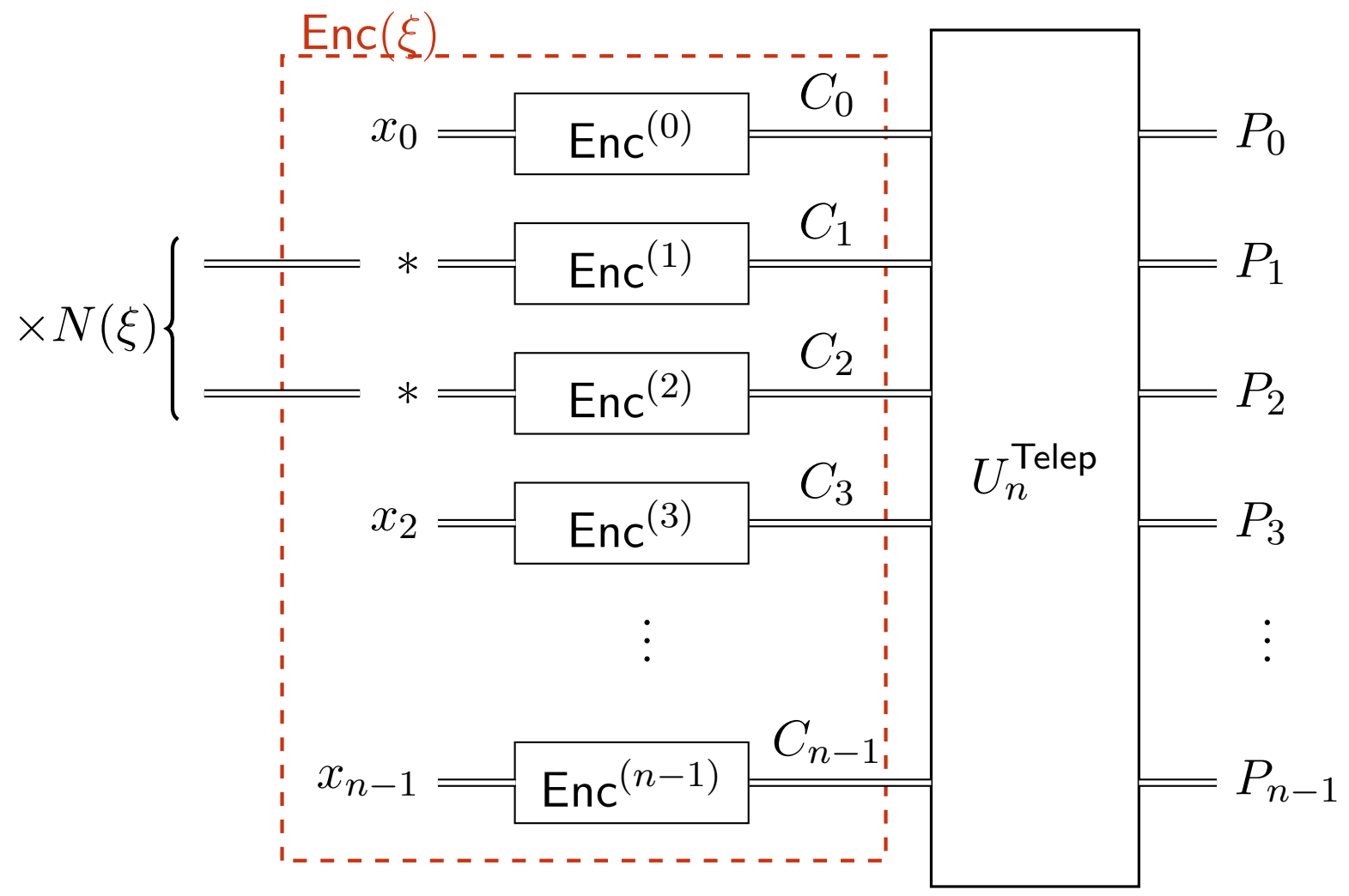

Supplementary Figure 9: The bit-string encoded version of the restricted gate-teleportation problem. Starting from a restriction $\xi : \mathbb{Z}^n \to \{0,1\}^5 \cup \{*\}$, the task can be understood as that of producing, on a given input $x \in (\{0,1\}^5)^m$, a Pauli P such that the pair $(\widehat{\mathsf{Enc}}(x), P)$ appears in the output distribution of the illustrated circuit. Here $\widehat{\mathsf{Enc}} \in \mathcal{E}^m$ is obtained by suitably restricting (according to active inputs of ξ) an n-tuple $\mathsf{Enc} \in \mathcal{E}^n$ of valid encodings of Cliffords.

The following is an "encoded" version of Theorem 4.1. We consider a restriction of the form $\xi : \mathbb{Z}^n \to \{0,1\}^5 \cup \{*\}$, i.e., it associates either a 5-tuple of bits or the symbol $*$ to each $i \in \mathbb{Z}_n$. We call $\xi^{-1}(\{*\}) \subset \mathbb{Z}_n$ the set of active inputs. Using an encoding function Enc, such a restriction ξ gives rise to a restriction which associates a Clifford or a symbol $*$ to every $i \in \mathbb{Z}_n$. By a slight abuse of notation, we denote this new restriction as $\mathsf{Enc}(\xi)$. It is defined as

$$\begin{array}{rccl} \mathsf{Enc}(\xi): & \mathbb{Z}_n & \to & \mathsf{Cliff} \cup \{*\} \\ & i & \mapsto & \mathsf{Enc}(\xi)(i) := \begin{cases} * & \text{if } \xi(i) = * \\ \mathsf{Enc}(\xi(i)) & \text{otherwise} \end{cases} \end{array} \qquad \text{for every } i \in \mathbb{Z}_n \ .$$

We may then consider the associated restricted problem for the restriction $\mathsf{Enc}(\xi)$, that is, the problem $R_{U_n^{\mathsf{Telep}}}\restriction_{\mathsf{Enc}(\xi)}$. It is illustrated in Supplementary Figure 9. We have the following statement:

Theorem 4.5. *Let $0 < \gamma \leq 1$ and $1/2 < \delta \leq 1$ be constants. Let $\mathsf{Enc} = (\mathsf{Enc}^{(0)}, \ldots, \mathsf{Enc}^{(n-1)}) \in \mathcal{E}^n$ be an n-tuple of valid encoding maps. Let $\xi : \mathbb{Z}_n \to \{0,1\}^5 \cup \{*\}$ be a restriction that keeps $m \geq \gamma n^\delta$ inputs active. Assume that the set of active inputs is $\xi^{-1}(\{*\}) = \{i_0 < \ldots < i_{m-1}\}$. Then any NC^0 circuit $\mathcal{C} : (\{0,1\}^5)^m \to \mathsf{Pauli}^n$ satisfies*

$$\Pr_{x \in (\{0,1\}^5)^m} \left[(\widehat{\mathsf{Enc}}(x), \mathcal{C}(x)) \in R_{U_n^{\mathsf{Telep}}}\restriction_{\mathsf{Enc}(\xi)} \right] < \frac{63}{64}$$

for a uniformly randomly chosen input $x = (x^{(0)}, \ldots, x^{(m-1)}) \in (\{0,1\}^5)^m$, where we introduced

$$\widehat{\mathsf{Enc}}(x) = (\mathsf{Enc}^{(i_0)}(x^{(0)}), \ldots, \mathsf{Enc}^{(i_{m-1})}(x^{(m-1)})) \in \mathsf{Cliff}^m \qquad \textit{for} \qquad x \in (\{0,1\}^5)^m \ .$$

A version of this result where the classical soundness parameter $\nu := 63/64$ can be substituted by an arbitrarily small positive constant is given in Theorem 7.6 in Section 7.

Proof. The proof is an immediate consequence of the proof of Theorem 4.1. The only difference is that we use forward light cones of blocks of input bits encoding Clifford gates, similar to Lemma 4.2. To reach a contradiction, we use Lemma 4.4 instead of Lemma 1.3. It gives a bound on the success probability of the bitstring-encoded variant of the magic-square game with random Cliffords. □

5 Quantum advantage against AC^0

One of the most important methods for circuit size lower bounds going beyond the light-cone arguments are the so-called restriction-based methods and in particular switching lemmas [13, 1, 26, 16, 24, 23] (for a nice exposition see Beame's article [3]). Informally, a strong variant of this technique asserts that if we randomly fix all but $O(n^\delta)$ (for $\delta \leq 1$) inputs of an AC^0-circuit, then with overwhelming probability, it is possible to fix a few additional bits to ensure that the restricted circuit can be implemented by an NC^0 circuit.

5.1 Bit-restrictions and block-restrictions

We will be interested in circuits $\mathcal{C}_n^{\mathsf{block}} : (\{0,1\}^5)^n \to (\{0,1\}^2)^n$ whose input-strings $x = (x^{(0)}, \ldots, x^{(n-1)})$ consist of $5n$ bits, and are naturally grouped into blocks (strings) of length 5: Each five-tuple $x^{(j)} \in \{0,1\}^5$ represents a Clifford group element $C_j \in \mathsf{Cliff}$, for $j \in \mathbb{Z}_n$. (The output can be interpreted similarly, but this is less important for this section.)

We will often interpret the circuit $\mathcal{C}_n^{\mathsf{block}}$ as a function $\mathcal{C}_n : \{0,1\}^{n'} \to \{0,1\}^{m'}$ where $n' = 5n$ and $m' = 2n$. (We omit the superscript block to distinguish this interpretation from the case where we group the input bits into blocks of 5.) We may then consider a "standard" restriction $\xi : \mathbb{Z}_{n'} \to \{0,1,*\}$ of the input bits. Applying such a restriction to the circuit $\mathcal{C}_n$, i.e., considering the circuit $\mathcal{C}_n \restriction_\xi$ (defined analogously as in Section 4.1 but for individual bits) may, however, not be compatible with the actual block-structure of the input of $\mathcal{C}_n$. In the encoded bit representation of Clifford group elements, a general restriction can mean that individual Clifford inputs are partially fixed (rather than being completely free or completely fixed), i.e., the restriction may mean that we have to consider a subset of Cliff in some entries.

Nevertheless, it will be convenient in this section to work with restrictions $\xi : \mathbb{Z}_{n'} \to \{0,1,*\}$ of individual bits. Let us call ξ a block restriction if it is compatible with the block structure. That is, we say that $\xi : \mathbb{Z}_{5n} \to \{0,1,*\}$ is a block restriction if and only if

$$(\xi(5j), \xi(5j+1), \ldots, \xi(5j+4)) \in \{0,1\}^5 \cup \{(*,*,*,*,*)\} \qquad \text{for every} \qquad j \in \mathbb{Z}_n\ . \quad (29)$$

Condition (29) expresses the fact that for every block of 5 input bits, the restriction assigns a 5-bit string or leaves the entire block (i.e., each bit in the block) free (i.e., active). We will interchangeably refer to a block restriction by the function $\xi : \mathbb{Z}_{5n} \to \{0,1,*\}$ (satisfying (29)) or the associated function $\xi^{\mathsf{block}} : \mathbb{Z}_n \to \{0,1\}^5 \cup \{*^{\mathsf{block}}\}$. Here $*^{\mathsf{block}}$ is a new symbol indicating that the whole block of 5 bits is not fixed.

Naturally, any block restriction $\xi : \mathbb{Z}_{5n} \to \{0,1,*\}$ determines a function

$$\begin{aligned}\xi^{\mathsf{block}} :\ \mathbb{Z}_n &\to \{0,1\}^5 \cup \{*^{\mathsf{block}}\}\\ j &\mapsto \xi^{\mathsf{block}}(j) := \begin{cases} *^{\mathsf{block}} & \text{if } \xi(j) = (*,*,*,*,*)\\ (\xi(5j), \xi(5j+1), \ldots, \xi(5j+4)) & \text{otherwise}\end{cases}\end{aligned}$$

as considered in Section 4.3. To connect to the corresponding result, our analysis will involve, in particular, the question of how likely it is that a randomly chosen restriction $\xi : \mathbb{Z}_{n'} \to \{0,1,*\}$ is a block restriction. We will also need to study the number $N(\xi^{\mathsf{block}}) = |(\xi^{\mathsf{block}})^{-1}\{*^{\mathsf{block}}\}|$ of free blocks in a block restriction arising in this way.

5.2 From an AC^0 circuit to an NC^0 circuit by random restrictions

Let $p_* \in [0,1]$. We consider the distribution R_{p_*} over random restrictions $\rho : \mathbb{Z}_n \to \{0,1,*\}$ defined as follows: To draw $\rho \sim \mathsf{R}_{p_*}$ according to R_{p_*}, independently set

$$\rho(\ell) = \begin{cases} * & \text{with probability} \quad p_* \\ 0 & \qquad\qquad\qquad (1-p_*)/2 \\ 1 & \qquad\qquad\qquad (1-p_*)/2 \end{cases}$$

for each input $\ell \in \mathbb{Z}_n$ at random.

Let us define concatenation of restrictions. Let $\rho : \mathbb{Z}_n \to \{0,1,*\}$ be restriction such that $N(\rho) = |\rho^{-1}(\{*\})|$ and $\rho^{-1}(*) = \{\iota(1) < \cdots < \iota(N(\xi))\}$. Let $\eta : \mathbb{Z}_{N(\rho)} \to \{0,1,*\}$ be another restriction. We define the concatenated restriction $\rho \restriction \eta$, i.e, first restricting by ρ and subsequently restricting by η, as

$$\begin{aligned} \rho \restriction \eta : \quad \mathbb{Z}_n \quad &\to \quad \{0,1,*\} \\ \ell \quad &\mapsto \quad (\rho \restriction \eta)(\ell) := \begin{cases} \rho(\ell) & \text{if} \;\; \rho(\ell) \neq * \\ \eta(\iota^{-1}(\ell)) & \text{if} \;\; \rho(\ell) = * \;\; \text{and} \;\; \eta(\iota^{-1}(\ell)) \neq * \;. \\ * & \text{otherwise} \;\; . \end{cases} \end{aligned}$$

The key result we build on here is the multi-switching lemma established in [4], which is itself adapted from [23] and extended to circuits outputting multiple bits. For any AC^0-circuit $\mathcal{C}$, this gives a bound on the probability that for a randomly chosen restriction ρ, the restricted function $\mathcal{C} \restriction_\rho$ has a certain structure (namely, that it is a composition of a decision tree with tuples of decision trees computing the output bitstring associated to each leaf). Here we do not need this structural statement, but only one consequence of it (which has also been discussed in [4]): the restricted circuit $\mathcal{C} \restriction_\rho$ can be restricted further by an additional restriction η to yield a circuit $(\mathcal{C} \restriction_\rho) \restriction_\eta$ which can be implemented by an NC^0 circuit. While the additional restriction η is a function of ρ and the circuit $\mathcal{C}$, this dependence only specifies which additional input bits should be fixed but the values can be arbitrary and hence also random.

To state this concisely, suppose $\mathcal{C} \colon \{0,1\}^n \to \{0,1\}^m$ and $\rho \colon \mathbb{Z}_n \to \{0,1,*\}$ are given. Let $N(\rho) = |\rho^{-1}(\{*\})|$ be the number of input bits that are free when the restriction ρ is applied to $\mathcal{C}$, i.e., the restricted circuit $\mathcal{C} \restriction_\rho$ is a function $\mathcal{C} \restriction_\rho \colon \{0,1\}^{N(\rho)} \to \{0,1\}^m$. We then say that a restriction $\eta : \mathbb{Z}_{N(\rho)} \to \{0,1,*\}$ fixes additional t bits of $\mathcal{C}$ if $N(\rho) - t$ bits are active when applying this restriction to $\mathcal{C} \restriction_\rho$, i.e., if $(\mathcal{C} \restriction_\rho) \restriction_\eta$ is a function $(\mathcal{C} \restriction_\rho) \restriction_\eta \colon \{0,1\}^{N(\rho)-t} \to \{0,1\}^m$.

Lemma 5.1 (Multi-switching lemma of [4], paraphrased). *Let $\mathcal{C} : \{0,1\}^n \to \{0,1\}^m$ be an AC^0 circuit of size $s = \mathsf{size}(\mathcal{C})$ and depth $d = \mathsf{depth}(\mathcal{C})$. Let q, t be arbitrary parameters. Set*

$$p_* := \frac{1}{m^{1/q} O(\ln s)^{d-1}} \;.$$

Suppose a random restriction $\rho : \mathbb{Z}_n \to \{0,1,*\}$ is chosen according to $\rho \sim \mathsf{R}_{p_*}$. Then the following holds with probability at least $1 - s2^{-t}$ taken over the choice of ρ. There is a subset $T \subset \rho^{-1}(\{*\})$ of the $N(\rho) = |\rho^{-1}(\{*\})|$ remaining free input bits (after application of ρ) of size at most $|T| \leq 2t$ such that for any restriction $\eta : \mathbb{Z}_{N(\rho)} \to \{0,1,*\}$ fixing exactly the inputs belonging to T, the restricted circuit $(\mathsf{C} \restriction_\rho) \restriction_\eta$ can be implemented by an NC^0 circuit (built from fan-in 2 AND and OR gates and NOT gates) of depth at most q.

In our problem we deal with inputs that represent Clifford gates and outputs that represents Pauli operators. With a binary encoding, we represent the input Clifford gates by blocks of

5 bits and the output Pauli operators by blocks of 2 bits. That is, an AC^0 circuit of interest have the block structure $\mathcal{C}_n : (\{0,1\}^5)^n \to (\{0,1\}^2)^n$. We will assume that the circuit depth is a constant $\mathsf{depth}(\mathcal{C}_n) = O(1)$.

We will use the multi-switching lemma (Lemma 5.1) to show that for suitably chosen parameters, there is a distribution P over block restrictions $\xi^{\mathsf{block}} : \mathbb{Z}_n \to \{0,1\}^5 \cup \{*^{\mathsf{block}}\}$ such that the following holds with high probability, i.e., with probability $1 - o(1)$, over the choice of $\xi^{\mathsf{block}} \sim P$. The restriction ξ^{block} has at least $N(\xi^{\mathsf{block}}) = |(\xi^{\mathsf{block}})^{-1}(\{*^{\mathsf{block}}\})| = \Omega(n^{3/4})$ active blocks and the block restricted circuit $\mathcal{C}_n \restriction_{\xi^{\mathsf{block}}}$ is implementable by an NC^0 circuit. Furthermore, let $S = (\xi^{\mathsf{block}})^{-1}(\{*^{\mathsf{block}}\})$ be the set of active blocks, then the distribution P conditioned on having S as the set of active blocks is uniform over block restrictions having the same set of active blocks. We show the following.

Theorem 5.2. *Let $\mathcal{C}_n : (\{0,1\}^5)^n \to (\{0,1\}^2)^n$ be an AC^0 circuit of size $s = \mathsf{size}(\mathcal{C}_n)$ and depth $d = \mathsf{depth}(\mathcal{C}_n)$. Assume that*

$$s \le e^{n^{1/(20d)}} . \tag{30}$$

Let n be sufficiently large. Then there is a probability distribution P over block restrictions

$$\xi^{\mathsf{block}} : \mathbb{Z}_n \to \{0,1\}^5 \cup \{*^{\mathsf{block}}\}$$

such that for $\xi^{\mathsf{block}} \sim P$ chosen at random the following properties are satisfied with probability at least

$$1 - \exp(-c_1(d) n^{1/2}) ,$$

where $c_1(d) > 0$ is a constant that only depends on d.

(i) The number $N(\xi^{\mathsf{block}}) = |(\xi^{\mathsf{block}})^{-1}(\{*^{\mathsf{block}}\})|$ of active blocks is greater than $c_2(d)n^{3/4}$, where $c_2(d) > 0$ is a constant that only depends on d.

(ii) The block restricted circuit $\mathcal{C}_n \restriction_{\xi^{\mathsf{block}}}: (\{0,1\}^5)^{N(\xi^{\mathsf{block}})} \to (\{0,1\}^2)^n$ is implementable by an NC^0-circuit (built from fan-in 2 AND and OR gates and NOT gates) of depth at most $20d$.

Furthermore $\xi^{\mathsf{block}} \sim P$ satisfies the following.

(iii) For any subset $S \subset \mathbb{Z}_n$, the distribution of ξ^{block} conditioned on $(\xi^{\mathsf{block}})^{-1}(\{*^{\mathsf{block}}\}) = S$ is uniform over the set of all block restrictions $\tilde{\xi}^{\mathsf{block}} : \mathbb{Z}_n \to \{0,1\}^5 \cup \{*^{\mathsf{block}}\}$ having $S = (\tilde{\xi}^{\mathsf{block}})^{-1}(\{*^{\mathsf{block}}\})$ as the set of active blocks.

Proof. We consider $\mathcal{C}_n$ as a circuit $\mathcal{C} : \{0,1\}^{n'} \to \{0,1\}^{m'}$ where

$$\begin{aligned} n' &= 5n , \\ m' &= 2n \end{aligned}$$

and apply the multi-switching lemma (Lemma 5.1) with the following choice of parameters:

$$\begin{aligned} q &= 20d , \\ t &= \frac{p_*^5 n'}{5} \cdot \frac{1}{5} = \frac{p_*^5 n}{5} \end{aligned} \tag{31}$$

and with the star probability from the assumption of Lemma 5.1

$$p_* = \frac{1}{(m')^{1/q} \cdot O(\ln s)^{d-1}} = \frac{1}{(2n)^{1/(20d)} \cdot O(\ln s)^{d-1}} .$$

This choice of parameters and the size assumption (30) means that the probability p_* can be bounded from below by

$$p_* \geq \frac{1}{(2n)^{1/(20d)} O(n^{1/(20d)})^{d-1}} = c(d) n^{-1/20} \;, \tag{32}$$

for some $c(d) > 0$ depending only on d, where we used that

$$(\ln s)^{d-1} \leq \left(n^{1/(20d)}\right)^{d-1} = n^{(d-1)/(20d)} \;,$$

by Eq. (30).

To estimate the probability $1 - s2^{-t}$ appearing in multi-switching lemma (Lemma 5.1), we argue that our choice of parameters also implies that, for all sufficiently large n,

$$s \leq 2^{t/2} \;. \tag{33}$$

Indeed, we have, for all sufficiently large n,

$$\begin{aligned}
\ln(2^{t/2}) &= \frac{t}{2} \ln 2 \\
&= \frac{\ln 2}{2 \cdot 5} \cdot p_*^5 n \\
&\geq \frac{\ln 2}{10} \cdot c(d)^5 \cdot n^{3/4} && \text{by (32)} \\
&\geq n^{1/(20d)} \\
&\geq \ln s && \text{by (30)} \;,
\end{aligned}$$

implying the claim (33).

The multi-switching lemma (Lemma 5.1) asserts that when a restriction $\rho : \mathbb{Z}_{n'} \to \{0, 1, *\}$ is drawn randomly according to $\rho \sim \mathsf{R}_{p_*}$, the probability that there is no restriction $\eta : \mathbb{Z}_{N(\rho)} \to \{0, 1, *\}$ of at most $2t$ of the remaining $N(\rho)$ free input bits such that $(\mathcal{C} \restriction_\rho) \restriction_\eta$ is implementable by an NC^0-circuit is bounded, for all sufficiently large n, by

$$\begin{aligned}
\Pr_{\rho \sim \mathsf{R}_{p_*}} [\forall \eta : (\mathcal{C} \restriction_\rho) \restriction_\eta \notin (\mathsf{NC}^0 \text{ of depth } 20d)] &\leq s2^{-t} \\
&\leq 2^{-t/2} \qquad \text{by Eq. (33).}
\end{aligned}$$

Here, by "NC^0 of depth $20d$", we mean the class of classical circuits built from fan-in 2 AND and OR gates and NOT gates, with depth at most $20d$. More precisely, it says the following: Consider the set $\mathcal{T}(\rho)$ consisting of all subsets $T \subset \rho^{-1}(\{*\})$ of size $|T| \leq 2t$ and the property that any restriction η of all the bits of T gives a circuit $(\mathcal{C} \restriction_\rho) \restriction_\eta$ that can be implemented by an NC^0-circuit. Then, for all sufficiently large n, this set is non-empty with high probability for a randomly chosen $\rho \sim \mathsf{R}_{p_*}$, i.e.,

$$\Pr_{\rho \sim \mathsf{R}_{p_*}} [\mathcal{T}(\rho) \neq \emptyset] \geq 1 - 2^{-t/2} \;. \tag{34}$$

Now consider the following probabilistic process defining a distribution P over restrictions $\xi : \mathbb{Z}_{n'} \to \{0, 1, *\}$, see Supplementary Figure 10 for an illustration.

1. Choose a restriction $\rho : \mathbb{Z}_{n'} \to \{0, 1, *\}$ randomly according to $\rho \sim \mathsf{R}_{p_*}$.
2. If the corresponding set $\mathcal{T}(\rho)$ is non-empty, arbitrarily pick an element $T \in \mathcal{T}(\rho)$. Then randomly and uniformly pick a restriction $\eta : \mathbb{Z}_{N(\rho)} \to \{0, 1, *\}$ among all restrictions that fix exactly the inputs associated with T. That is, choose $|T|$ bits $(b_1, \ldots, b_{|T|}) \in \{0, 1\}^{|T|}$ uniformly and independently at random, and then define η by T (the set of fixed bits) and these bits.

   If the set $\mathcal{T}(\rho)$ is empty, set $\eta(x) = *$ for all $x \in \mathbb{Z}_{N(\rho)}$.

3. Consider the concatenated restriction $\eta \restriction \rho : \mathbb{Z}_{n'} \to \{0, 1, *\}$. Partition

$$\mathbb{Z}_{n'} = \bigcup_{j \in \mathbb{Z}_n} B_j \ , \qquad \text{where} \qquad B_j = \{5j, 5j+1, \ldots, 5j+4\} \ ,$$

into blocks consisting of 5 bits each. We call a block B_j partially fixed if $0 < |B_j \cap (\eta \restriction \rho)^{-1}(\{*\})| < 5$, i.e., if the corresponding blocks contains both bits fixed by the restriction $\eta \restriction \rho$, as well as bits that are free. Then randomly choose a restriction τ that fixes (only) every free bit in every partially fixed block B_j, for $j \in \mathbb{Z}_n$, among all restrictions with this property. This amounts to choosing uniformly random bits and assigning them to each free bit located in a partially fixed block.

4. Output the concatenated restriction $\xi := \tau \restriction \eta \restriction \rho : \mathbb{Z}_{n'} \to \{0, 1, *\}$.

We claim that the distribution P over restrictions $\xi : \mathbb{Z}_{n'} \to \{0, 1, *\}$ has the required properties.

Observe first that Step 3 ensures that ξ is a block restriction, i.e., can be interpreted as a function $\xi^{\mathsf{block}} : \mathbb{Z}_n \to \{0, 1\}^5 \cup \{*^{\mathsf{block}}\}$ with certainty. Indeed, this step guarantees that each block is either completely fixed or completely free. Furthermore, since in the process defining ξ, every bit which is fixed is fixed to a uniformly chosen bit, property (iii) of the distribution P is satisfied by construction.

It remains to argue that the constructed distribution P satisfies both Properties (i) and (ii) with high probability. For property (ii) this follows immediately from the switching lemma because of the choice of η in step 2, the fact that fixing bits of an NC^0-circuit preserves NC^0-implementability, and the bound (34): We have

$$\begin{aligned} \Pr_{\xi \sim P} [\xi \text{ does not satisfy (ii)}] &\leq 2^{-t/2} \\ &= 2^{-p_*^5 n/10} && \text{by the choice (31)} \\ &\leq 2^{-c(d)^5 n^{3/4}/10} && \text{by inequality (32)} \end{aligned} \tag{35}$$

For property (i), we need to estimate the number $N(\xi^{\mathsf{block}}) := |\xi^{-1}(\{*^{\mathsf{block}}\})|$ of active blocks. Let $N(\rho)$ be the number of blocks that are (completely) free in the restriction $\rho \sim \mathsf{R}_{p_*}$ chosen in step (1). Since the proposed procedure involves fixing at most $2t$ additional bits in Step 2, the number of free blocks in $\rho \restriction \eta$ can be lower bounded by

$$N(\eta \restriction \rho) \geq N(\rho) - 2t \ .$$

The subsequent step (3) only affects partially fixed block and thus does not change the number of free blocks: We have $N(\xi^{\mathsf{block}}) = N(\eta \restriction \rho)$ and thus

$$N(\xi^{\mathsf{block}}) \geq N(\rho) - 2t \ . \tag{36}$$

It thus remains to study the random variable $N(\rho)$. By definition of the distribution R_{p_*}, the random variable $N(\rho)$ is distributed according to the binomial distribution $\mathsf{BIN}(n'/5, p_*^5)$ with expectation

$$\mathbb{E}[\mathsf{BIN}(n'/5, p_*^5)] = \frac{n'}{5} p_*^5 = n p_*^5 \ .$$

In particular, using Hoeffding's inequality $\Pr[Z \leq \mathbb{E}[Z] - \gamma] \leq \exp\left(-(2\gamma^2)/n\right)$ for a random variable $Z \sim \mathsf{BIN}(n, q)$ and $\gamma \leq \mathbb{E}[Z]$, we obtain

$$\Pr_{\rho \sim \mathsf{R}_{p_*}} \left[N(\rho) \leq \frac{n p_*^5}{2} \right] \leq \exp\left(-\frac{n p_*^{10}}{2} \right) \ ,$$

or, with (36),

$$\Pr_{\xi\sim P}\left[N(\xi^{\mathsf{block}}) \geq \frac{np_*^5}{2} - 2t\right] \geq \Pr_{\rho\sim\mathsf{R}_{p_*}}\left[N(\rho) \geq \frac{np_*^5}{2}\right]$$
$$\geq 1 - \Pr_{\rho\sim\mathsf{R}_{p_*}}\left[N(\rho) \leq \frac{np_*^5}{2}\right]$$
$$\geq 1 - \exp\left(-\frac{np_*^{10}}{2}\right) \ . \quad (37)$$

By the choice (31) of t we have

$$\frac{np_*^5}{2} - 2t = \frac{np_*^5}{10} \geq \frac{c(d)^5}{10}n^{3/4} \ ,$$

where we used (32) to obtain the inequality. Similarly

$$\frac{1}{2}np_*^{10} \geq \frac{c(d)^{10}}{2}n^{1/2} \ ,$$

inequality (37) implies

$$\Pr_{\xi\sim P}\left[N(\xi^{\mathsf{block}}) \geq \frac{c(d)^5}{10}n^{3/4}\right] \geq 1 - \exp\left(-\frac{c(d)^{10}}{2}n^{1/2}\right) \ . \quad (38)$$

We define $c_1(d) = \frac{1}{2}\min\{c(d)^{10}/2, c(d)^5 \cdot \ln 2/10\}$ and $c_2(d) = c(d)^5/10$. Combining (38) and (35) with the union bound shows that for randomly chosen $\xi \in P$, the event

$$\left(N(\xi) \geq c_2(d)n^{3/4}\right) \quad \text{and} \quad \left(\mathcal{C}\restriction_\xi \in (\mathsf{NC}^0 \text{ of depth } 20d)\right)$$

occurs, for every sufficiently large n, with probability at least

$$1 - \left(\exp\left(-\frac{c(d)^{10}}{2}n^{1/2}\right) + 2^{-\frac{c(d)^5}{10}n^{3/4}}\right) \geq 1 - \exp\left(-c_1(d)n^{1/2}\right) \ .$$

This completes the proof. □

5.3 An AC^0 circuit size lower bound for the single-qubit gate-teleportation problem

In this section, we complete the proof for the average-case AC^0 size lower bound on the single-qubit gate-teleportation problem over uniformly random instances.

Since the multi-switching lemma works with circuits taking binary inputs, we show an average case result for AC^0-circuits for uniformly random bitstrings encoding Clifford gates by maps defined in Section 4.2, see Eq. (27). We then rely on results from Section 4.2 to obtain our average case circuit size lower bound for AC^0 circuits: Here we consider uniformly random sequences of Cliffords as input.

Theorem 5.3. *Let* $\mathsf{Enc} \in \mathcal{E}^n$ *be an arbitrary* n*-tuple of valid encoding maps as described in Section 4.2. Suppose* $\mathcal{C}_n : (\{0,1\}^5)^n \to \mathsf{Pauli}^n$ *is a depth-*d AC^0 *circuit which solves the bit encoded single-qubit gate-teleportation problem with probability at least* 0.985 *on average for a bitstrings* $x \in (\{0,1\}^5)^n$ *sampled uniformly at random for all sufficiently large* n*, i.e.,*

$$\Pr_{x\in(\{0,1\}^5)^n}\left[(\mathsf{Enc}(x), \mathcal{C}_n(x)) \in R_{U_n^{\mathsf{Telep}}}\right] \geq 0.985 \ .$$

Then the circuit has size at least

$$\mathsf{size}(\mathcal{C}_n) > e^{n^{1/(20d)}} \ .$$

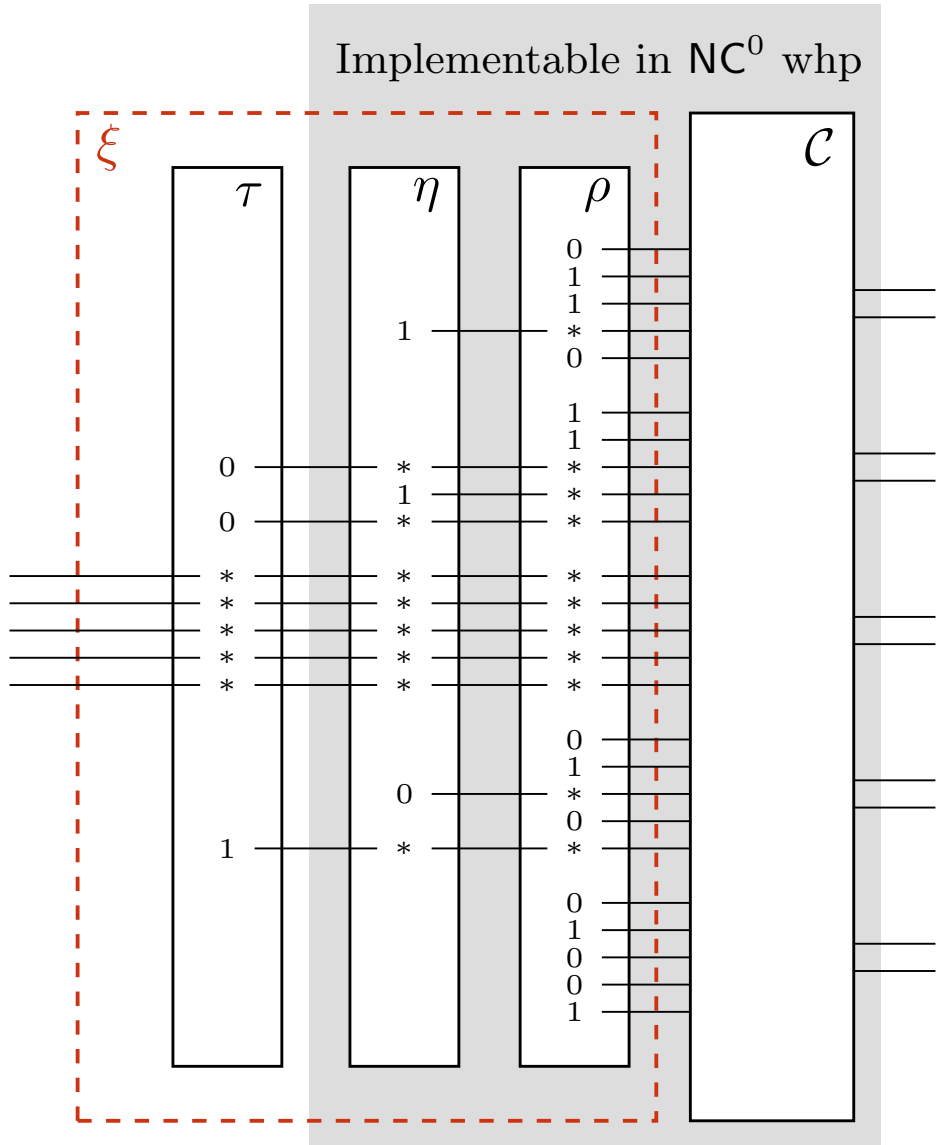

Supplementary Figure 10: Schematic representation of the multiple layers of restriction maps used in the proof of Theorem 5.2: the circuit $\mathcal{C}$ restricted by ρ and subsequently by η is with high probability implementable by an NC^0 circuit and so is $\mathcal{C}$ restricted by block restriction $\xi = \tau \upharpoonright \eta \upharpoonright \rho$.

In Section 7 (see Theorem 7.7), we give a version of this result where instead of the classical soundness parameter $\nu := 0.985$, a positive constant arbitrarily close to 0 can be chosen.

Proof. For the sake of contradiction, assume there exists an n-tuple $\mathsf{Enc} \in \mathcal{E}^n$ of valid encoding maps and an AC^0 circuit $\mathcal{C}_n : (\{0,1\}^5)^n \to \mathsf{Pauli}^n$ having depth $d = \mathsf{depth}(\mathcal{C}_n)$ and $\mathsf{size}(\mathcal{C}_n) = s \leq e^{n^{1/(20d)}}$ such that

$$\Pr_{x \in (\{0,1\}^5)^n} \left[(\mathsf{Enc}(x), \mathcal{C}_n(x)) \in R_{U_n^{\mathsf{Telep}}} \right] \geq 0.985 \ .$$

By Theorem 5.2 there is a probability distribution P over block restrictions $\xi^{\mathsf{block}} : \mathbb{Z}_n \to \{0,1\}^5 \cup \{*^{\mathsf{block}}\}$ such the distribution P satisfies the condition (iii) and a randomly chosen $\xi^{\mathsf{block}} \sim P$ satisfies (i) and (ii) with probability at least

$$1 - \exp\left(-c_1(d) n^{1/2}\right) \ ,$$

where $c_1(d)$ only depends on the circuit depth d. Let us restate these conditions here for convenience:

(i) The number of active blocks satisfies $N(\xi^{\mathsf{block}}) \geq c_2(d) n^{3/4}$, for $c_2(d)$ depending only on d.

(ii) The block restricted circuit $\mathcal{C}_n \upharpoonright_{\xi^{\mathsf{block}}}$ can be implemented by an NC^0 circuit (consisting of fan-in 2 *AND* and *OR* gates and *NOT* gates) $\mathcal{C}'_n$ of depth $20d$.

(iii) For a restriction $\xi^{\mathsf{block}} \sim P$ drawn according to the distribution P the following holds. For any subset $S \subset \mathbb{Z}_n$, the distribution of ξ^{block} conditioned on $S = (\xi^{\mathsf{block}})^{-1}(\{*^{\mathsf{block}}\})$ is uniform over all block restrictions $\tilde{\xi}^{\mathsf{block}} : \mathbb{Z}_n \to \{0,1\}^5 \cup \{*^{\mathsf{block}}\}$ having S as the set of active blocks, i.e., $S = (\tilde{\xi}^{\mathsf{block}})^{-1}(\{*^{\mathsf{block}}\})$.

Let us use $(\xi^{\mathsf{block}})^{-1}(\{*^{\mathsf{block}}\}) = \{i_0 < i_1 < \ldots < i_{N(\xi^{\mathsf{block}})-1}\}$ to denote the active blocks of ξ^{block}. We omit the superscript in ξ^{block} and $*^{\mathsf{block}}$ in the following, and simply write $\xi : \mathbb{Z}_{5n} \to$

$(\{0,1\}^5)^n \times \{*\}$. Let us define the bit-encoding

$$\widehat{\mathsf{Enc}}_\xi(y) = (\mathsf{Enc}^{(i_0)}(y^{(0)}), \ldots, \mathsf{Enc}^{(i_{N(\xi)-1})}(y^{(N(\xi)-1)})) \in \mathsf{Cliff}^{N(\xi)} \qquad \text{for} \qquad y \in (\{0,1\}^5)^{N(\xi)} \ .$$

for input instances of the $R_{U_n^{\mathsf{Telep}}} \restriction_{\mathsf{Enc}(\xi)}$. From Condition (iii) we have

$$\Pr_{x\in(\{0,1\}^5)^n}\left[(\mathsf{Enc}(x), \mathcal{C}_n(x)) \in R_{U_n^{\mathsf{Telep}}}\right] = \Pr_{\substack{\xi\sim P \\ y\in(\{0,1\}^5)^{N(\xi)}}}\left[\left(\widehat{\mathsf{Enc}}_\xi(y), \mathcal{C}_n \restriction_\xi (y)\right) \in R_{U_n^{\mathsf{Telep}}} \restriction_{\mathsf{Enc}(\xi)}\right] \tag{39}$$

Recall that with probability at least $1 - \exp(-c_1(d)n^{1/2})$ the block-restriction ξ is such that there exists an NC^0 circuit $\mathcal{C}'_n$ implementing the restricted circuit $\mathcal{C}_n \restriction \xi$, and this circuit solves instances of size at least $c_2(d)n^{3/4}$. By Theorem 4.5 we have, for all sufficiently large n, that

$$\Pr_{y\in(\{0,1\}^5)^{N(\xi)}}\left[\left(\widehat{\mathsf{Enc}}_\xi(y), \mathcal{C}'_n(y)\right) \in R_{U_n^{\mathsf{Telep}}} \restriction_{\mathsf{Enc}(\xi)}\right] < \frac{63}{64} \ ,$$

over the uniform choice of instances from $(\{0,1\}^5)^{N(\xi)}$.

Therefore we can bound (39) by using the law of total probability, obtaining

$$\begin{aligned}\Pr_{x\in(\{0,1\}^5)^n}\left[(\mathsf{Enc}(x), \mathcal{C}_n(x)) \in R_{U_n^{\mathsf{Telep}}}\right] &< \exp(-c_1(d)n^{1/2}) + \left(1 - \exp(-c_1(d)n^{1/2})\right) \cdot \frac{63}{64} \\ &< 0.985 \ ,\end{aligned}$$

for all sufficiently large n. This is a contradiction. □

By using Lemma 4.3, we obtain the following corollary.

Corollary 5.4. *For all sufficiently large n, the following holds. Suppose $\mathcal{C}_n : \mathsf{Cliff}^n \to \mathsf{Pauli}^n$ is a depth-d AC^0-circuit which solves the single-qubit gate-teleportation problem with probability at least* 0.986 *on average over uniformly randomly chosen instances, i.e.,*

$$\Pr_{C\in\mathsf{Cliff}^n}\left[(C, \mathcal{C}_n(C)) \in R_{U_n^{\mathsf{Telep}}}\right] \geq 0.986 \ .$$

Then the circuit size of $\mathcal{C}_n$ is at least

$$\mathsf{size}(\mathcal{C}_n) > e^{n^{1/(20d)}} \ .$$

In Section 7 (see Corollary 7.8), we show that an analogous statement holds with the soundness parameter $\nu := 0.986$ replaced by any positive constant $\nu \in (0,1)$.

This together with Theorem 2.1 implies the following corollary.

Corollary 5.5 ($1D$-local $\mathsf{QNC}^0 \not\subseteq \mathsf{AC}^0$)**.** *The relation problem $R_{U_n^{\mathsf{Telep}}}$ separates the (relational) complexity classes AC^0 and $1D$-local QNC^0 of polynomial size constant-depth classical circuit with unbounded AND and OR gates as well as NOT gates and $1D$-local constant-depth quantum circuits, i.e., we have ($1D$-local) $\mathsf{QNC}^0 \not\subseteq \mathsf{AC}^0$.*

In contrast to the previous work [4], where a similar result was achieved with ideal $2D$-local constant depth quantum circuits, i.e., ($2D$-local) $\mathsf{QNC}^0 \not\subseteq \mathsf{AC}^0$, Corollary 5.5 establishes average case quantum advantage already with ideal $1D$-local constant depth quantum circuits. This stronger starting point is crucial to obtain the main result of this paper — a quantum advantage with noisy constant depth $3D$-local quantum circuits against AC^0.

6 Quantum advantage with noisy shallow 3D-local circuits against AC^0

In [6], an advantage of ideal shallow quantum circuits against NC^0-circuits (based on the so-called $1D$ magic-square problem) was lifted to an advantage of noisy $3D$-local shallow quantum circuits. This was achieved by incorporating fault tolerance in a non-standard manner into the considered computational problems. We give a brief overview of the general method developed in [6] in Section 6.4. This technique was subsequently applied in [15] to establish a number of complexity-theoretic separations (in both non-interactive and interactive settings) between noisy shallow quantum circuits and classical shallow circuits, but without locality considerations.

6.1 Noisy quantum circuits: Local stochastic noise

To state our result in detail, we use the following notions formalizing (certain) noisy quantum circuits. These were first proposed in pioneering work by Gottesman [14], see also e.g., [11]. A Pauli error E on n qubits (in the following indexed by $\mathbb{Z}_n = \{1, \ldots, n\}$) is a random variable taking values in $\mathsf{Pauli}(n) := \{I, X, Y, Z\}^{\otimes n}$. We denote by $\mathrm{supp}(E) \subseteq [n]$ the (random) subset of qubits acted on non-trivially by E and call this the support of E. We define the p-local stochastic noise as Pauli errors non-trivially affecting any subset of qubits with a probability exponentially suppressed in the size of the subset, as follows.

Definition 6.1 (p-local stochastic noise). *The Pauli error E is called p-local stochastic noise for parameter $p \in [0, 1]$ called the noise strength if*

$$\Pr\left[F \subseteq \mathrm{supp}(E)\right] \leq p^{|F|} \qquad \textit{for every subset} \qquad F \subseteq [n] \ .$$

We write $E \sim \mathcal{N}(p)$ to indicate that E is p-local stochastic noise.

In the following, we consider noisy implementations of processes where a Clifford circuit $U = C_D \cdots C_1$ on n qubits of constant depth $D = O(1)$ is applied to an initial product state $|0^n\rangle$, and the resulting final state is measured in the computational basis. Here each C_j is a gate layer consisting of one- and two-qubit Clifford gates acting on disjoint subsets of qubits. We assume that local stochastic noise E_j acts after each gate layer C_j. For $j \in [D-1]$ this models errors occurring during execution of the gates inside C_j. The error E_D accounts for both errors in C_D as well as readout (measurement) errors. To model errors in the state preparation, we additionally include local stochastic noise E_0 before the application of the first gate layer C_1. Overall, this noisy process results in a sample drawn from the distribution

$$\tilde{p}^U(z) = |\langle z| E_D C_D \cdots E_2 C_2 E_1 C_1 E_0 \, |0^n\rangle \, |^2 \cdot P(E_0, \ldots, E_D) \tag{40}$$

by Born's rule. We constrain the distribution $P(E_0, \ldots, E_D)$ of errors as follows: We make the assumption each of the errors E_j, $j = 0, \ldots, D$ is local stochastic with parameter p, i.e., $E_j \sim \mathcal{N}(p)$. Importantly, this is an assumption on the marginal distribution P_{E_j} of each error only, and the joint distribution $P(E_0, \ldots, E_D)$ may include dependencies. We refer to this process, i.e., sampling from the distribution (40), as an noisy implementation of U with error strength p.

6.2 Fault-tolerance building blocks

The construction of [6] makes use of a CSS-type [10, 25] quantum code $\mathcal{Q}_m$ encoding a single logical qubit into m physical qubits. This code needs to satisfy three conditions: A set of (logical) Clifford group generators should have transversal implementations, logical states should

be preparable in a single-shot fashion, and read-out should also be possible in a single-shot way. Here single-shot refers to fault-tolerance protocols which do not rely on repeated syndrome measurements, following seminal work by Bombín [5]. We refer to a code satisfying these condition as suitable. Formally, this is defined as follows.

Definition 6.2. *Let $\mathcal{Q}_m$ be a CSS-code encoding a single logical qubit into m physical qubits. The code is called suitable if it satisfies the following three conditions:*

Transversal Clifford gates: *The code $\mathcal{Q}_m$ has transversal logical Clifford gates: the logical Hadamard gate, the logical phase gate $S = \mathsf{diag}(1, i)$ and the logical CNOT-gate between two copies of $\mathcal{Q}_m$ have transversal implementations, i.e., can be realized by depth-1 circuits consisting of one- and two-qubit Clifford gates.*

Single-shot state preparation: *The code $\mathcal{Q}_m$ allows for fault-tolerant single-shot state preparation. That is, there is*

(i) a constant-depth quantum circuit W acting on m data qubits and m_{aux} auxiliary qubits, as well as

(ii) two functions $\mathsf{Rep} : \mathsf{Pauli}(m+m_{aux}) \to \mathsf{Pauli}(m)$ and $\mathsf{Rec} : \{0,1\}^{m_{aux}} \to \mathsf{Pauli}(m)$ and two constants $c', c'' > 0$

such that

$$(\mathsf{Rec}(s) \otimes |s\rangle\langle s|) E W (|0^m\rangle \otimes |0^{m_{aux}}\rangle) \propto \mathsf{Rep}(E) \left|\overline{0}\right\rangle \otimes |s\rangle \qquad \textit{for all} \qquad s \in \{0,1\}^{m_{aux}}$$

for any Pauli error $E \in \mathsf{Pauli}(m + m_{aux})$, and

$$\mathsf{Rep}(E) \sim \mathcal{N}(c' p^{c''}) \qquad \textit{whenever} \qquad E \sim \mathcal{N}(p) \tag{41}$$

for any $p \in [0,1]$.

Single-shot readout: *The code allows for single-shot readout, i.e., there is*

(i) a realization $\overline{Z} = \prod_{j\in\mathrm{supp}(\overline{Z})} Z_j$ of the logical Pauli-Z operator, as well as

(ii) a function $\mathsf{Dec} : \{0,1\}^m \to \{0,1\}$, a (noise strength) threshold $q_{thres} > 0$ and constants $c, c' > 0$

such that the following holds. Let $\mathsf{Parity} : \{0,1\}^m \to \{0,1\}$ be the function $\mathsf{Parity}(x) = \sum_{j\in\mathrm{supp}(\overline{Z})} x_j \pmod 2$ defined by the support $\mathrm{supp}(\overline{Z})$ of $\overline{Z}$. Let $\mathcal{L} \subset \{0,1\}^m$ be the subspace of strings which obey all Z-stabilizers, i.e., $\sum_{j\in\mathrm{supp}(S_Z)} x_j \pmod 2 = 0$ for every Z-type stabilizer S_Z. Then we have

$$\Pr\left[\mathsf{Dec}(x \oplus v) = \mathsf{Parity}(x)\right] \geq 1 - \exp(-c' m^c) \qquad \textit{for all} \qquad x \in \mathcal{L} \tag{42}$$

for any $q < q_{thres}$ and any random variable v taking values in $\{0,1\}^m$ such that $X(v) \sim \mathcal{N}(q)$. Here $\oplus$ denotes bitwise addition modulo two

Let us briefly comment on the meaning of these different conditions defining suitability of a code:

The first condition requires that a set of generators of the (logical) Clifford group has favorable implementations in terms of fault-tolerance: This is because error propagation is limited under a depth-1 circuit.

The single-shot state preparation condition specifies a fault-tolerant protocol for preparing the encoded state $\left|\overline{0}\right\rangle$ and provides a guarantee on its behavior. The latter proceeds as follows.

The constant-depth circuit W is applied to $|0^m\rangle \otimes |0^{m_{\text{aux}}}\rangle$, i.e., a product state of the system and auxiliary qubits (all prepared in the state $|0\rangle$). Subsequently, the auxiliary qubits are measured in the computational basis, yielding a measurement outcome $s \in \{0,1\}^{m_{\text{aux}}}$. The post-measurement state (given outcome s) on the system qubits is the logical state $|\overline{0}\rangle$ (the $+1$ eigenstate of the logical Pauli operator $\overline{Z}$) up to a Pauli correction $\mathsf{Rec}(s)$ determined by the measurement outcome $s \in \{0,1\}^{m_{\text{aux}}}$. If an error E occurs before the measurement, then the prepared logical state $|\overline{0}\rangle$ is replaced by a state having a residual Pauli error $\mathsf{Rep}(E)$. Condition (41) states that the strength of this residual error is bounded in terms of a polynomial function of the strength p of the original error $E \sim \mathcal{N}(p)$.

Similarly, the single-shot readout condition shows that the logical $\overline{Z}$-observable of an encoded state can be measured fault-tolerantly by a simple protocol proceeding as follows: Every qubit of the code is measured in the computational basis, yielding a measurement outcome $x \in \{0,1\}^m$. Subsequently, the value $\mathsf{Dec}(x)$ is returned. We note that for a code state $|\overline{\Psi}\rangle \in \mathcal{Q}_m$, the measurement outcome $x \in \{0,1\}^m$ belongs with certainty to the subspace $\mathcal{L}$ of strings which obey all Z-stabilizers. If instead, a state $E|\overline{\Psi}\rangle$ corrupted by a Pauli error $E = X(v)Z(w)$, $v, w \in \{0,1\}^m$ is measured in the computational basis, the measurement outcome is of the form $x \oplus v$ for $x \in \mathcal{L}$. Determination of the expectation value $\langle\overline{\Psi}, \overline{Z\Psi}\rangle$ from the measurement outcome $x \oplus v$ is possible if $\mathsf{Parity}(x)$ can be computed from $x \oplus v$. Condition (42) states that this is the case with high probability when E is local stochastic with a sufficient small noise strength, i.e., noise below some threshold error strength q_{thres}.

Fault-tolerant implementation of a classically controlled Clifford circuit. Consider a classically controlled Clifford circuit U acting on n qubits (and classical control bits). Assume that U has constant depth D. Following [6], we write describe the action of such a circuit in terms of a family $\{C_b\}_{b\in\{0,1\}^v}$ of n-qubit Clifford circuits indexed by the classical input $b \in \{0,1\}^v$. Each Clifford circuit C_b is composed of Clifford gates that act non-trivially on at most $k = O(1)$ qubits and has depth at most D. The action of U is then given by

$$U(|\Psi\rangle \otimes |b\rangle) = (C_b|\Psi\rangle) \otimes |b\rangle \qquad \text{for all} \qquad |\Psi\rangle \in (\mathbb{C}^2)^{\otimes n} \text{ and } b \in \{0,1\}^v .$$

We consider the situation where on input $b \in \{0,1\}^v$, the circuit is applied to the initial state $|0^n\rangle \otimes |b\rangle$, and the output state of the n qubits is measured in the computational basis. This results in a sample z drawn from the distribution

$$p_b^U(z) = |\langle z|C_b 0^n\rangle|^2 \qquad \text{for} \qquad z \in \{0,1\}^n . \tag{43}$$

A suitable quantum code $\mathcal{Q}_m$ in the sense of Definition 6.2 gives rise to an implementation of this process which is fault-tolerant against local stochastic noise below some threshold strength. This fault-tolerant implementation relies on an extended classically controlled circuit U^{ext} on $n\cdot(m+m_{\text{aux}})$ qubits and v classical control bits, as well as classical post-processing of the measurement outcome. The circuit U^{ext} (cf. [6, Figure 8]) is applied to an initial state of the form $(|0^m\rangle \otimes |0\rangle^{m_{\text{aux}}})^{\otimes n}) \otimes |b\rangle$. Here in each of the n factors, the first m qubits correspond to an encoded logical qubit of the initial circuit U (excluding classical controls), whereas the latter m_{aux} qubits are auxiliary qubits for single-shot logical state preparation. The remaining v qubits in the state $|b\rangle$ correspond to the classical control. Let us describe the action of U^{ext} for a classical input $b \in \{0,1\}^v$. It proceeds by applying the state preparation unitary W to each of the m factors. Subsequently, a logical implementation $\overline{C_b}$ of the Clifford C_b is applied to the $\mathcal{Q}_m$-encoded logical qubits. This is realized by replacing each (classically controlled) Clifford gate in C_b by the corresponding transversal implementation.

Now consider the process of

(i) applying U^{ext} to the initial state $(|0^m\rangle \otimes |0\rangle^{m_{\text{aux}}})^{\otimes n}) \otimes |b\rangle$,

(ii) subsequently measuring each auxiliary qubit in the computational basis obtaining outcomes $s = (s^1, \ldots, s^n) \in (\{0,1\}^{m_{\text{aux}}})^n$, and

(iii) measuring each of the remaining $n \cdot m$ qubits obtaining outcomes $y = (y^1, \ldots, y^n) \in (\{0,1\}^m)^n$.

This results in a sample (s, y) drawn from the distribution

$$p_b^{U^{\text{ext}}}(s, y) = |(\langle y| \otimes \langle s|)(\overline{C}_b \otimes I)W^{\otimes n}(|0^{mn}\rangle \otimes |0^{m_{\text{aux}}n}\rangle|^2 .$$

The process described here involving the extended unitary U^{ext} can be used in a black-box manner to generate a sample from the original distribution (43) by post-processing the measurement result (s, y). Furthermore, even if its realization is affected by local stochastic noise acting in between gate layers, the resulting distribution is close to the ideal distribution (43), see Theorem 6.3 below. The correct post-processing map can be described as follows. It is obtained by propagating the Pauli operator $\mathsf{Rec}(s^1) \otimes \cdots \otimes \mathsf{Rec}(s^n)$ associated with the syndrome $s = (s^1, \ldots, s^n)$ through the logical gate $\overline{C_b}$. More precisely, only the Pauli-X-part of the corresponding operators matters. That is, consider the functions $f, h : \{0,1\}^{nm_{\text{aux}}} \times \{0,1\}^v \to (\{0,1\}^m)^n$ defined by

$$\overline{C_b}(\mathsf{Rec}(s^1) \otimes \cdots \otimes \mathsf{Rec}(s^n))\overline{C_b} \propto X(f(s,b))Z(h(s,b)) \tag{44}$$

for all $s = (s^1, \ldots, s^n) \in (\{0,1\}^{m_{\text{aux}}})^n$ and $b \in \{0,1\}^v$. Where we used the notation $X, Z : \{0,1\}^n \to \mathsf{Pauli}^n$ to denote the strings of respective Paulis $X(s) := X_1^{s^1} \cdots X_n^{s^n}$ and $Z(s) := Z_1^{s^1} \cdots Z_n^{s^n}$. Denoting by f^i the restriction of the output of f to the i-th codeblock, a sample (s, y) produced by a potentially noisy implementation of U^{ext} on input $b \in \{0,1\}^v$ should be then postprocessed as follows: Compute

$$z_i = \mathsf{Dec}(y^i \oplus f^i(s,b)) \qquad \text{for} \qquad i \in [n] \tag{45}$$

and output $z = (z_1, \ldots, z_n) \in \{0,1\}^n$.

This post-processing leads to an outcome as described in the following Theorem 6.3. This statement is a slight reformulation of [6, Theorem 17] to which we refer to for the proof. It is a consequence of suitability conditions in Definition 6.2 and the fact that propagating local stochastic errors past through low-depth Clifford circuits composed of one- and two-qubit gates preserves local stochasticity (albeit increasing the noise strength in a controlled manner, see e.g., [6, Lemma 11]).

Theorem 6.3. *Let $\mu \in (0,1)$ be arbitrary. Let U be a constant-depth, classically controlled Clifford circuit on n qubits, with v classical control bits. Consider a noisy implementation of the extended circuit U^{ext} on input $b \in \{0,1\}^v$ resulting in an output $(s, y) \in (\{0,1\}^{m_{aux}})^n \times (\{0,1\}^m)^n$. We assume that the noise strength is $p \in [0,1]$. Let $z \in \{0,1\}^n$ be the output of the post-processing map defined by (45). Then there is a choice of $m = O(\mathsf{poly}(\log n))$ and a threshold value p_{thres} (depending only on the depth of U) such that the following holds for any $p < p_{thres}$. The sample z produced by this noisy process is drawn from a distribution q_b that is close in L^1-norm to the the distribution (43), i.e.,*

$$\left\|q_b - p_b^U\right\|_1 < \mu \ . \tag{46}$$

We refer to μ as the quantum completeness parameter. We note that [6, Theorem 17] states this result for the concrete choice $\mu = 0.01$. However, inspecting the argument of Ref. [6] shows that an analogous statement holds for any (arbitrarily small) $\mu \in (0,1)$, and an identical threshold p_{thres} (independent of μ), provided $m = O(\mathsf{poly}(\log n))$ is chosen sufficiently large. In

other words, the quantum completeness parameter μ only enters the choice of constants in the expression $O(\mathsf{poly}(\log n))$.

The construction discussed in Theorem 6.3 is not exactly what is needed to show a fault-tolerant quantum advantage, for two reasons: First, the given construction does not involve locality considerations: We have made no assumptions, e.g., about a spatial arrangement of the qubits. In particular, we have not considered the question of whether or not the gates in the single-shot preparation circuit W are (geometrically) local. Even if this is the case (for some geometry), this does not necessarily mean that the extended circuit U^{ext} is also geometrically local because the latter involves n logical qubits. As discussed in the introduction, non-local circuits mean that the local stochastic noise model is not physically well-motivated. We address this issue in Section 6.3. There we argue that the circuits relevant to our work are geometrically local in $3D$.

The second reason for which Theorem 6.3 is not directly applicable to quantum advantage considerations is the post-processing involved. As stated, Theorem 6.3 constitutes what can be considered a "standard" use of fault tolerance: Here a noisy implementation is used to weakly (approximately) simulate an ideal quantum circuit as expressed by Eq. (46). In addition to the overhead in the number of qubits and gates, this comes at the cost of having to classically compute the "correction map" given by the post-processing operation (45). The functions $f^i : (\{0,1\}^{m_{\text{aux}}})^n \to \{0,1\}^m$ used here generally fall outside the confines of constant-depth (classical) circuits, and should therefore not appear in a quantum circuit for demonstrating an advantage of shallow circuits. We address how this issue is resolved (following [6]) in Section 6.4: The post-processing can be incorporated into a new computational problem while preserving the hardness for classical circuits. This is what we mean by "non-standard" use of fault-tolerance techniques.

6.3 3D-local fault-tolerant building blocks

In [6], it was shown that the so-called folded surface code [19, 17] (a variant of the original surface code [8]) is a suitable quantum code $\mathcal{Q}_m$ in the sense of Definition 6.2: Transversality of the relevant single-qubit gates was shown by Moussa in earlier work [19]. A proof that single-shot decoding is possible below a threshold error strength was given in [6] following ideas from Ref. [12]. Single-shot state preparation is the most non-trivial aspect of the analysis of [6]. Here a slight variant of the condition was shown: namely, the given circuit prepares a logical Bell state (up to Pauli errors) encoded in two copies of the folded surface code (We note that it is also possible to obtain a single-shot state preparation procedure for the logical state $|\overline{0}\rangle$ from this construction.) In fact, this single-shot logical Bell state preparation is key to making our circuit (as well as the one considered in [6]) local.

The relevant variant of Single-shot state preparation in Definition 6.2 is obtained by replacing the logical state $|\overline{0}\rangle \in (\mathbb{C}^2)^{\otimes m}$ by the logical Bell state $|\overline{\Phi}\rangle = \frac{1}{\sqrt{2}}(|\overline{00}\rangle + |\overline{11}\rangle) \in (\mathbb{C}^2)^{\otimes m} \otimes (\mathbb{C}^2)^{\otimes m}$ encoded in two copies of the code $\mathcal{Q}_m$. It reads as follows:

Single-shot Bell-state preparation: The code $\mathcal{Q}_m$ allows for fault-tolerant single-shot preparation of logical Bell state if there are

(i) a constant-depth quantum circuit W acting on $2m$ data qubits and m_{aux} auxiliary qubits,

(ii) two functions $\mathsf{Rep} : \mathsf{Pauli}(2m + m_{\text{aux}}) \to \mathsf{Pauli}(2m)$ and $\mathsf{Rec} : \{0,1\}^{m_{\text{aux}}} \to \mathsf{Pauli}(2m)$ and two constants $c', c'' > 0$

such that

$$(\mathsf{Rec}(s) \otimes |s\rangle\langle s|) EW(|0^{2m}\rangle \otimes |0^{m_{\mathrm{aux}}}\rangle) \propto \mathsf{Rep}(E)\,|\overline{\Phi}\rangle \otimes |s\rangle \qquad \text{for all} \qquad s \in \{0,1\}^{m_{\mathrm{aux}}}$$

for any Pauli error $E \in \mathsf{Pauli}(2m + m_{\mathrm{aux}})$, and

$$\mathsf{Rep}(E) \sim \mathcal{N}(c' p^{c''}) \qquad \text{whenever } E \sim \mathcal{N}(p) \ .$$

A realization by a local circuit. The construction given in [6] satisfies Definition 6.2 with the relevant Single-shot Bell-state preparation and with the additional property that the circuit W used for the single-shot state preparation is local in $3D$. Here we only give a brief overview of the geometric arrangement of qubits, see [6] for details. We define an interaction graph associated with a quantum circuit (composed of single- and two-qubit gates) as a graph whose vertices are associated with qubits and whose edges are associated with two-qubit gates between them. The locality of the circuit is defined in terms of the corresponding interaction graph: we say that a circuit is $3D$-local if this graph admits an embedding in $\mathbb{R}^3$ in such a way that there is a constant $\kappa > 0$ such that the Euclidean distance between any two adjacent vertices is upper bounded by κ, and any ball of radius κ contains only $O(1)$ qubits. For example, this is the case when the interaction graph is a regular lattice.

The construction of [6] has polylogarithmic dependence

$$m, m_{\mathrm{aux}} \in \Theta(\mathsf{poly}(\log n)) \tag{47}$$

of the parameters m, m_{aux} on n. The $2m$ qubits (corresponding to two copies of the code $\mathcal{Q}_m$) in the definition of W are located on the "left" and "right" triangular faces of a wedge (prism) in $3D$, see Supplementary Figure 11. Each of these two triangular faces form a square sublattice and has m vertices associated with m qubits. The remaining m_{aux} auxiliary qubits constitute the "bulk" of the prism; they are arranged on a regular lattice embedded in the wedge. If d is the linear size of the prism (which is proportial to the distance of the underlying surface code), then $m \in \Theta(d^2)$ and $m_{\mathrm{aux}} \in \Theta(d^3)$. The scaling (47) is a consequence of the choice $d = \Theta(\mathsf{poly}(\log n))$.

To describe the action of the circuit W, it is helpful to recall how the wedge-like geometric arrangement arises. The construction is based on the fact that the cluster state on a 3D cubic lattice of size $L \times L \times L$ has localizable entanglement on two opposing triangluar faces even if corrupted by noise. This was first established in seminal work by Raussendorf, Bravyi, and Harrington [21]: they showed that measuring bulk qubits in the cluster state results in a surface-code encoded Bell state on the two opposing triangular faces, up to a Pauli correction determined by the measurement outcomes. This was shown to be the case even for a certain thermal noise model. The analysis of [6] generalizes this result to local stochastic noise by establishing single-shot Bell-state preparation for the surface code. The corresponding cluster state can be generated from a product state by a depth-6 $3D$-local Clifford circuit. In the context of quantum advantage experiments, one actually needs the folded surface code instead in have transversal Clifford gates. This is obtained by folding the cubic lattice along a diagonal plane, resulting in the described wedge. Thus W is a depth-6 Clifford circuit generating a folded version of the cluster state.

Geometric locality of the extended circuit. Consider a classically controlled Clifford circuit U specified by a family $\{C_b\}_{b\in\{0,1\}^k}$ of Clifford circuits. As explained in [6, p. 24 in Supplementary Information], the associated fault-tolerant extension U^{ext} built using a suitable quantum code $\mathcal{Q}_m$ (see Definition 6.2) is generally not geometrically local. Non-local operations may be required as a consequence of any of the following:

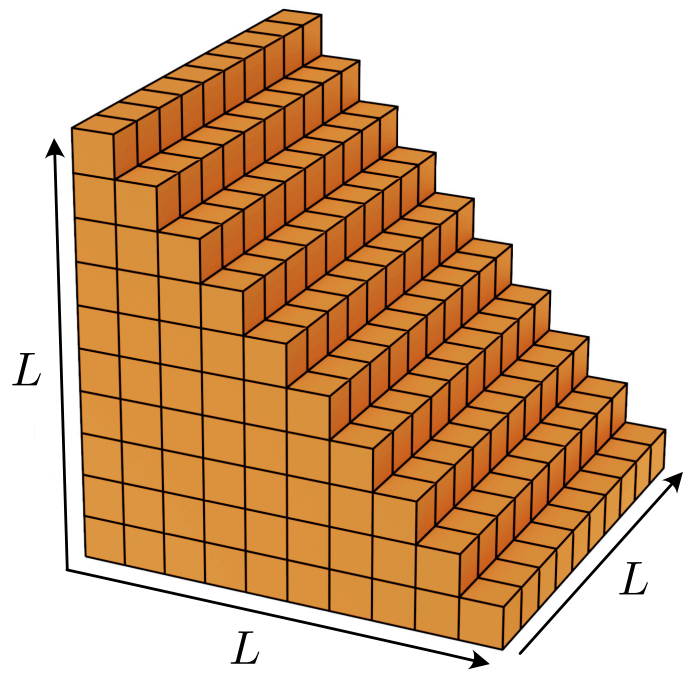

Supplementary Figure 11: Geometric arrangement of qubits, forming a prism, in the definition of the single-shot Bell-state preparation procedure. The linear size L of the prism is indicated.

(i) The state preparation unitary W involves non-local gates.

(ii) The Clifford circuit C_b involves non-local (two-qubit) Clifford gates.

(iii) The transversal implementation of a logical one- or two-qubit Clifford gate may act on all qubits of one or two codeblocks, respectively. If this is the case and the gate is classically controlled, the classical control may need to be geometrically non-local.

Non-locality in classical controls as in (iii) are the least problematic here because they can be addressed by copying corresponding classical input (control) bits. This is achieved by suitably modifying the computational problem, see Section 6.4.

Fortunately, for certain circuits of interest including the gate-teleportation circuit considered in our work, the potential non-locality arising from (i) can be avoided by using the $3D$-local Bell state preparation circuit W. Furthermore, the issue (ii) does not arise in our setup because the gate-teleportation circuit is geometrically $1D$-local (with qubits arranged on a ring). This is similar to the circuit for the extended magic-square game considered in [6], which was geometrically $1D$-local (with qubits arranged on a line). In particular, this means that the fault-tolerant extension U^{ext} associated with the gate-teleportation circuit U_n^{Telep} can be made $3D$-local following the arguments of [6, p. 52]. We state this as follows, where we identify the single-qubit Clifford group (corresponding to inputs) with a subset of $\{0,1\}^5$ (and similarly, identify Paulis (outputs) with a subset of $\{0,1\}^2$). To achieve locality also of the classical controls, each 5-tuple of the input $b \in (\{0,1\}^5)^n$ needs to be provided at m locations, i.e., copies of the corresponding bits are provided in several locations as input.

Theorem 6.4. *Let $\mu \in (0,1)$ be arbitrary. Let U_n^{Telep} be the gate-teleportation circuit on n qubits. Then an associated fault-tolerant circuit U^{ext} can be realized in a $3D$-local manner using a total of $O(n\mathsf{poly}(\log n))$ qubits. Assuming that copies of the input bits $b \in (\{0,1\}^5)^n$ are provided in the relevant locations, the associated interaction graph is obtained by gluing together wedges along a circle, resulting in a graph that tesselates a Colosseum-shaped $3D$ manifold that is homeomorphic to a solid torus in $\mathbb{R}^3$, see Supplementary Figure 12. There is a constant threshold $p_{thres} > 0$ such that for all $p < p_{thres}$, the input-output pair (b,z) obtained by post-processing the measurement result (s,y) for input $b \in (\{0,1\}^5)^n$ according to (45) satisfies*

$$\Pr\left[(b,z) \in R_{U_n^{\mathsf{Telep}}}\right] > 1-\mu$$

for any noisy implementation of U^{ext} with error strength p.

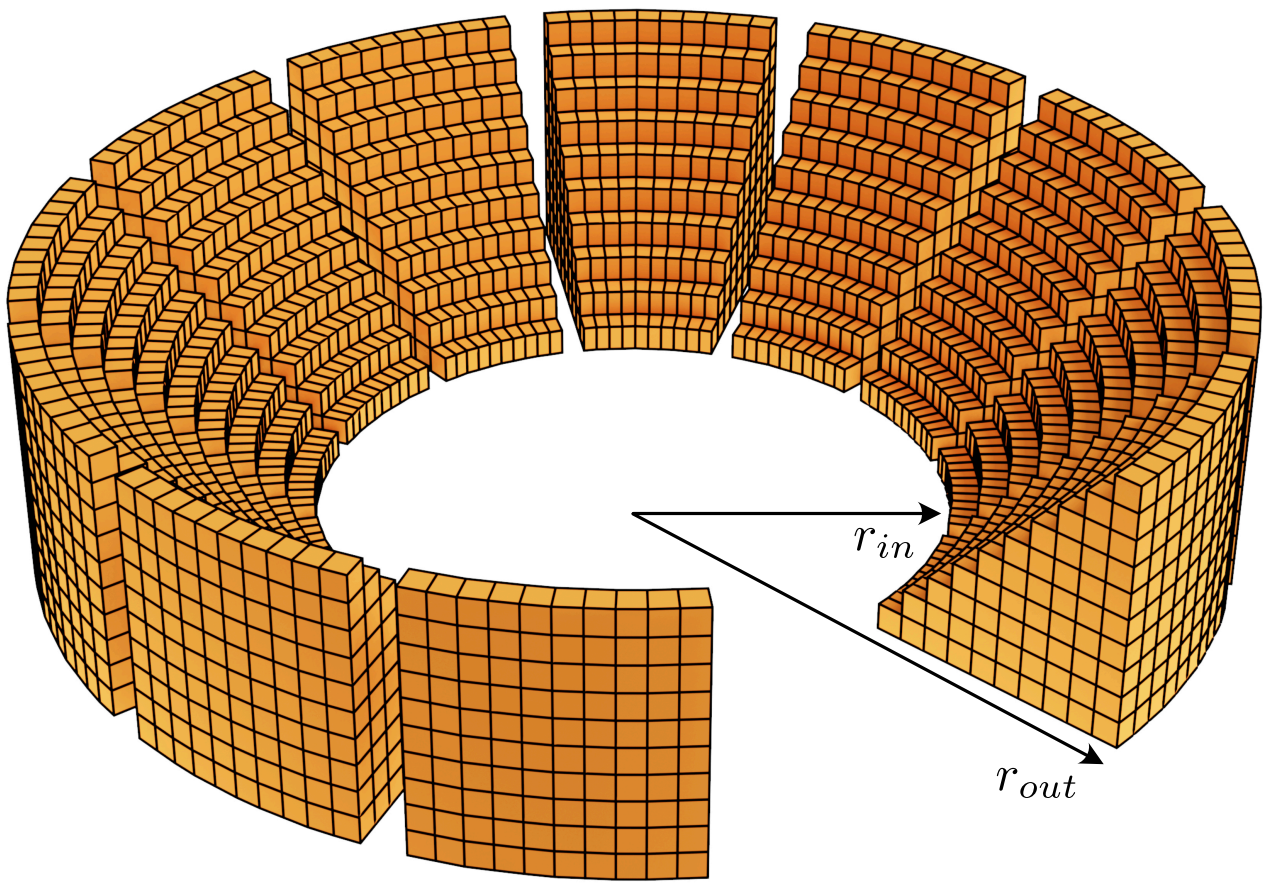

Supplementary Figure 12: The Colosseum-shaped embedding in $\mathbb{R}^3$ of the interaction graph associated with the $3D$-local circuit realizing the fault-tolerant circuit U^{ext}. By suitable post-processing, the circuit's output can be used to fault-tolerantly compute a valid solution to the relation problem $R_{U_n^{\mathsf{Telep}}}$ defined by the single-qubit gate-teleportation circuit U_n^{Telep}. The inner radius r_{in} and outer radius r_{out} of the Colosseum are indicated. The gaps between the prisms (see Supplementary Figure 11) are for illustration purposes only; neighboring triangular faces are connected in the interaction graph, as illustrated in Figure 3 of the main paper.

$3D$-locality of the circuit is a consequence of the fact that there are n wedges of polylogarithmic size: Each wedge has $\Theta(L)$ sites on each of its 9 bounding edges (when considered as a prism), with $L = \mathsf{poly}(\log n)$, see Eq. (47). Let r_{in} and r_{out} be the inner and outer radius of the Colosseum, i.e., r_{in} is the radius of the arena, whereas r_{out} is the radius of the outer wall (see Supplementary Figure 12). Let Δ_{out} be the geodesic distance between two neighboring sites at the base of the outer wall, i.e., on a circle of radius r_{out}. We similarly define Δ_{in}. Let Δ_R be the "radial" (Euclidean) distance between two neighboring sites on the ground as one moves radially outwards from the arena. Then

$$r_{in} = r_{out} - L \cdot \Delta_R \tag{48}$$

by definition. Since there are

$$N_{out} = N_{in} = L \cdot n$$

points on the outer, as well as inner circle, we have

$$\Delta_{out} = \frac{2\pi r_{out}}{N_{out}} = \frac{2\pi}{L \cdot n} r_{out}$$
$$\Delta_{in} = \frac{2\pi r_{in}}{N_{in}} = \frac{2\pi}{L \cdot n} r_{in} \ .$$

Inserting (48) it follows that

$$\Delta_{in} = \Delta_{out} - \frac{2\pi \Delta_R}{n} \ .$$

In particular, choosing e.g., $\Delta_R = \Delta_{out} =: \Delta$ constant, it follows Δ_{in} is lower bounded by a constant (for any sufficiently large n). Since the geodesic distance on a circle of radius r is equivalent to the Euclidean distance in $\mathbb{R}^2$ (with constants independent of r), it follows that for this choice, the Euclidean distance between any pair of neighboring sites is both upper and lower bounded by a constant.

6.4 Rendering quantum advantages noise-resilient

Here we discuss how a quantum advantage can be made fault-tolerant. Our arguments follow [6]. Consider a classically controlled Clifford circuit U that demonstrates a quantum advantage against a classical circuit class, e.g., consider the gate-teleportation circuit U^{Telep} and AC^0. As discussed in Section 6.2, any classically controlled Clifford circuit U such as U^{Telep} gives rise to a circuit U^{ext} with the property that the output of the latter can be post-processed to emulate the action of U. Even though this provides a fault-tolerant solution of the problem R_U of possibilistically simulating U, one cannot directly conclude that U^{ext} demonstrates a quantum advantage against the same classical circuit class. This is because of the additional classical post-processing that is required in the process. Instead, the idea of [6] is to argue that the circuit U^{ext} solves a computational problem $\mathcal{R}_U$ different from the original problem R_U, which is also computationally intractable for the classical circuit class considered.

As with the original problem R_U, the computational problem $\mathcal{R}_U$ is a relation problem, and we can specify it as a subset of input/output-pairs. Concretely, it is the subset

$$\mathcal{R}_U \subset \{0,1\}^v \times (\{0,1\}^{nm_{\mathrm{aux}}} \times \{0,1\}^{nm})$$

defined as follows: We have $(b,(s,y)) \in \mathcal{R}_U$ if and only if

$$(b,z) \in R_U \qquad \text{where} \qquad z_i = \mathsf{Dec}(y^i \oplus f^i(s,b)) \qquad \text{for} \qquad i \in [n] \ .$$

The definition of this problem and Theorem 6.3 immediately imply that even a noisy implementation of U^{ext} provides a solution to the problem $\mathcal{R}_U$ with probability greater than $1-\mu$, for any input. That is, we have

Theorem 6.5. *([6, Theorem 17]) There is a constant threshold p_{thres} such that the following holds for any $\mu \in (0,1)$. Any noisy implementation of the circuit U^{ext} with error strength $p < p_{thres}$ provides, on an arbitrary input $b \in \{0,1\}^v$, an output (s,y) such that*

$$\Pr\left[(b,(s,y)) \in \mathcal{R}_U\right] > 1-\mu \ .$$

It remains to argue that the problem $\mathcal{R}_U$ is hard for classical circuits if this is the case for R_U. This is a consequence of the following result, where we specialize [6, Theorem 18] to the setting of interest here. It shows that a solution to the computational problem $\mathcal{R}_U$ can be "decoded" by an AC^0-circuit to one for the original problem R_U. We note that the observation that this post-processing function can be realized by an AC^0-circuit of quasipolynomial size was first made in [15, Lemma 16].

Theorem 6.6. *([6, Theorem 18] and [15, Lemma 16]) There is an AC^0-circuit $\mathcal{C}_{\mathsf{post}}$ of depth*

$$\mathsf{depth}(\mathcal{C}_{\mathsf{post}}) \in O(\mathsf{depth}(U))$$

and size

$$\mathsf{size}(\mathcal{C}_{\mathsf{post}}) \in O(\exp(\mathsf{poly}(\log n)))$$

such that the following holds. Given any valid solution $(b,(s,y)) \in \mathcal{R}_U$ of the relation problem $\mathcal{R}_U$, the output $(b,z) = \mathcal{C}_{\mathsf{post}}((b,(s,y)))$ of the circuit $\mathcal{C}_{\mathsf{post}}$ is a valid solution of the problem R_U, i.e., $(b,z) \in R_U$.

Proof. As argued in [6, Proof of Theorem 18], the output $z \in \{0,1\}^n$ can be computed from (s,y) from the post-processing defined by (45) by a classical circuit $\mathcal{C}_{\mathsf{post}}$. This circuit first computes the usual $2n$-bit representation of the n-qubit Pauli operator $\mathsf{Rec}(s) = \bigotimes_{j=1}^n \mathsf{Rec}(s^j)$. Here s^j

consists of m_{aux} bits of s associated with the j-th logical qubit. This is achieved by considering each of the n copies of the function $\mathsf{Rec} : \{0,1\}^{m_{\text{aux}}} \to \mathsf{Pauli}(m)$. As noted in [15, Lemma 16], this function can be implemented by an AC^0-circuit of depth 4 and size $O(\exp(m_{\text{aux}}))$ using a truth-table.

Once $\mathsf{Rec}(s) \in \mathsf{Pauli}(m)^{\otimes n} \subset \mathsf{Pauli}(n \cdot m)$ has been computed, we can obtain $f^i(s,b)$ for any fixed $i \in [n]$ as follows. By relying on the standard stabilizer formalism and working in a gate-by-gate fashion, the result $P \mapsto CPC^\dagger$ of propagating a single Pauli operator P through a Clifford circuit C composed of 1- and 2-qubit gates of depth D can be computed by a classical circuit of depth $O(D)$ and size $O(D)$ whose gates have fan-in at most 4. Recalling the definition of the logical circuit $\overline{C}_b$ in terms of transversal Clifford gates, we conclude that for any Pauli operator $P \in \mathsf{Pauli}(n \cdot m)$, the function $b \mapsto \overline{C}_b P \overline{C}_b^\dagger$ can be computed by a circuit of depth $O(\mathsf{depth}(U))$, size $O(n \cdot m)$ and fan-in 4. By definition of f (see Eq. (44)), this means that $f^i(s,b)$ can be computed from (s,b) by an AC^0-circuit of depth $O(\mathsf{depth}(U))$ and size $O(\exp(m_{\text{aux}}))$.

Once $f^i(s,b)$ has been computed, the output z_i (see Eq. (45)) can be obtained by first taking the bitwise XOR $y^i \oplus f^i(s,b)$ of two m_{aux}-bit strings. This can be achieved by a depth-1 circuit of m_{aux} XOR-gates of fan-in 2. Finally, we apply the function $\mathsf{Dec} : \{0,1\}^m \to \{0,1\}$. We again note that Dec can be computed by an AC^0-circuit of depth 4 and size $O(\exp(m))$ by using a truth-table.

In summary, for each $i \in [n]$, the bit z_i is computed by an AC^0-circuit of constant depth and size at most $O(\exp(\max\{m_{\text{aux}}, m\}))$. Recalling that in our case, $\max\{m_{\text{aux}}, m\} = m_{\text{aux}}$ has a polylogarithmic dependence on n and the claim follows. □

6.5 Noise-resilient quantum advantage against AC^0 with a $3D$-local architecture

We can now show our main result:

Theorem 6.7 ((Sub)exponential quantum advantage)**.** *Let $\mu \in (0, 0.014)$ be arbitrary. There are functions $n_i(n), n_o(n) \in \Theta(n\mathsf{poly}(\log n))$, a relation*

$$\tilde{\mathcal{R}}_n \subset \{0,1\}^{n_i(n)} \times \{0,1\}^{n_o(n)} ,$$

a set of instances $\tilde{S}_n \subset \{0,1\}^{n_i(n)}$, a threshold $p_{thres} > 0$ and a constant $\delta > 0$ such that the following holds for all sufficiently large n.

(i) There is a classically controlled, Colosseum-shaped (Supplementary Figure 12), $3D$-local constant-depth quantum circuit $\tilde{U}$ such that for any noisy implementation of $\tilde{U}$ with error strength $p < p_{thres}$, the output $o \in \{0,1\}^{n_o(n)}$ produced by measurement on an arbitrary input $i \in \tilde{S}_n$ satisfies $(i,o) \in \tilde{\mathcal{R}}_n$ with probability at least $1-\mu$.

(ii) Suppose $\mathcal{C}$ is an AC^0-circuit which – with an average probability greater than 0.986 over a uniformly chosen instance $i \in \tilde{S}_n$ – produces an output $o \in \{0,1\}^{n_o(n)}$ such that $(i,o) \in \tilde{\mathcal{R}}_n$. Then its size is at least $\mathsf{size}(\mathcal{C}) > e^{n^{1/(\delta \mathsf{depth}(\mathcal{C}))}}$.

In Section 7 (see Theorem 7.10), we show that for a suitably defined relation, an analogous statement can be shown with the classical soundness parameter $\nu := 0.986$ replaced by an arbitrarily small constant $\nu \in (0,1)$. In other words, a quantum advantage can be demonstrated with any pair $(\mu, \nu) \in (0,1)^2$ of quantum completeness and classical soundness parameters subject to $\nu < 1-\mu$. In particular, the "classical–quantum" gap (difference in probabilities) $1-\mu-\nu$ can be arbitrarily close to 1.

Proof. Recall that the circuit U_n^{Telep} takes elements of Cliff^n as input and produces (upon measurement in the computational basis), an output from Pauli^n. Representing each output Pauli by 2 bits, the fault-tolerance construction of interest is essentially that given by a relation

$$\mathcal{R}_{U_n^{\mathsf{Telep}}} \subset \mathsf{Cliff}^n \times \left(\{0,1\}^{(2n)m_{\text{aux}}} \times \{0,1\}^{(2n)m}\right)$$

where $m, m_{\text{aux}} = \mathsf{poly}(\log n)$, see Eq. (47). The only modification we need to make to $\mathcal{R}_{U_n^{\mathsf{Telep}}}$ is to provide the relevant input entry (Clifford) at each of the n pairs of the $2m$ pairwise neighboring physical qubits (associated with two boundaries of two neighboring wedges) encoding neighboring (logical) qubits. This allows for a local classical control of the transversal Clifford gates in the associated quantum circuit. We also embed the set Cliff into $\{0,1\}^5$ with an arbitrary (but fixed) injective map. This leads to a trivially modified relation

$$\begin{aligned}\tilde{\mathcal{R}}_n &\subset \{0,1\}^{5nm} \times \left(\{0,1\}^{(2n)m_{\text{aux}}} \times \{0,1\}^{(2n)m}\right)\\ &\subset \{0,1\}^{\Theta(n\mathsf{poly}(\log(n)))} \times \left(\{0,1\}^{\Theta(n\mathsf{poly}(\log n))} \times \{0,1\}^{\Theta(n\mathsf{poly}(\log n))}\right)\\ &= \{0,1\}^{\Theta(n\mathsf{poly}(\log(n)))} \times \{0,1\}^{\Theta(n\mathsf{poly}(\log n))}\end{aligned}$$

as claimed. The described way of copying information about input Cliffords naturally defines an injective map $\mathsf{copy} : \mathsf{Cliff}^n \to \{0,1\}^{5nm}$. We define the set $\tilde{S}_n$ of instances as the image $\tilde{S}_n := \mathsf{copy}(\mathsf{Cliff}^n)$ of the set Cliff^n of all instances of the original problem $R_{U_n^{\mathsf{Telep}}}$.

The claim (i) is shown in Theorem 6.5, see the arguments before Theorem 6.4 for the $3D$-locality of the corresponding circuit.

Let us now turn to the proof of Claim (ii). Theorem 6.6 tells us that there is an AC^0-circuit $\mathcal{C}_{\mathsf{post}}$ that, given a pair $(b,(s,y)) \in \mathcal{R}_U$, produces a pair $(b,z) \in R_U$. Furthermore, $\mathcal{C}_{\mathsf{post}}$ is of constant depth $\mathsf{depth}(\mathcal{C}_{\mathsf{post}}) \in O(1)$ (since this is the case for U^{Telep}) and its size is at most $\exp(\mathsf{poly}(\log n))$. Define

$$\delta := 40(1 + \mathsf{depth}(\mathcal{C}_{\mathsf{post}})) \ .$$

The proof of Claim (ii) is now by contradiction: Assume that there is an AC^0-circuit $\mathcal{C}$ satisfying

$$\mathsf{size}(\mathcal{C}) < e^{n^{1/(\delta \mathsf{depth}(\mathcal{C}))}} \tag{49}$$

such that (for sufficiently large n)

$$\Pr_{b\in\tilde{S}_n}[(b,\mathcal{C}(b)) \in \mathcal{R}_n] \geq 0.986 \ .$$

Concatenating both circuits we obtain an AC^0-circuit $\mathcal{C}_{\mathsf{post}} \circ \mathcal{C}$ of depth

$$\begin{aligned}\mathsf{depth}(\mathcal{C}_{\mathsf{post}} \circ \mathcal{C}) &= \mathsf{depth}(\mathcal{C}) + \mathsf{depth}(\mathcal{C}_{\mathsf{post}})\\ &\leq (1 + \mathsf{depth}(\mathcal{C}_{\mathsf{post}})) \cdot \mathsf{depth}(\mathcal{C})\\ &\leq \frac{\delta}{40}\mathsf{depth}(\mathcal{C})\end{aligned} \tag{50}$$

and size

$$\begin{aligned}\mathsf{size}(\mathcal{C}_{\mathsf{post}} \circ \mathcal{C}) &= \mathsf{size}(\mathcal{C}) + \mathsf{size}(\mathcal{C}_{\mathsf{post}})\\ &< e^{n^{1/(\delta\mathsf{depth}(\mathcal{C}))}} + \exp(\mathsf{poly}(\log n))\\ &< e^{n^{2/(\delta\mathsf{depth}(\mathcal{C}))}}\\ &\leq e^{n^{1/(20\mathsf{depth}(\mathcal{C}_{\mathsf{post}}\circ\mathcal{C}))}} \qquad \text{by Eq. (50)}\end{aligned}$$

which satisfies

$$\Pr_{s\in\tilde{S}_n}\left[(\mathsf{copy}^{-1}(s),\mathcal{C}_{\mathsf{post}}\circ\mathcal{C}(s))\in R_{U_n^{\mathsf{Telep}}}\right]\geq 0.986\ .$$

Therefore we can use the concatenated circuit $\mathcal{C}_{\mathsf{post}}\circ\mathcal{C}$, which satisfies

$$\mathsf{size}(\mathcal{C}_{\mathsf{post}}\circ\mathcal{C})<e^{n^{1/(20\mathsf{depth}(\mathcal{C}_{\mathsf{post}}\circ\mathcal{C}))}}$$

to solve the original problem $R_{U_n^{\mathsf{Telep}}}$ with average probability at least 0.986 over uniformly random instances from $\tilde{S}_n$. By definition, $\tilde{S}_n$ is a set of all images of n-tuples $C\in\mathsf{Cliff}^n$ obtained by the copy function. This means that

$$\Pr_{s\in\tilde{S}_n}\left[(\mathsf{copy}^{-1}(s),\mathcal{C}_{\mathsf{post}}\circ\mathcal{C}(s))\in R_{U_n^{\mathsf{Telep}}}\right]=\Pr_{C\in\mathsf{Cliff}^n}\left[(C,\mathcal{C}_{\mathsf{post}}\circ\mathcal{C}\circ\mathsf{copy}(C))\in R_{U_n^{\mathsf{Telep}}}\right]\ ,$$

where we slightly abused the notation and allowed the function copy to accept inputs in the form of a Clifford gate as well as binary representation of Clifford gate. This gives us a circuit $\mathcal{C}'=\mathcal{C}_{\mathsf{post}}\circ\mathcal{C}\circ\mathsf{copy}$ that solves $R_{U_n^{\mathsf{Telep}}}$ with average probability at least 0.986 over uniformly random instances from Cliff^n. This circuit has size at most

$$\mathsf{size}(\mathcal{C}')<e^{n^{1/(20\mathsf{depth}(\mathcal{C}'))}}\ ,$$

since copy can be realised as fan-out of the input nodes and does not contribute to the standard definition of circuit size.

On the other hand, Corollary 5.4 tells us that any AC^0 circuit $\mathcal{C}''_n$ that solves the $R_{U_n^{\mathsf{Telep}}}$ problem with average success probability

$$\Pr_{C\in\mathsf{Cliff}^n}\left[\big(C,\mathcal{C}''_n(C)\big)\in R_{U_n^{\mathsf{Telep}}}\right]\geq 0.986$$

has size at least

$$\mathsf{size}(\mathcal{C}''_n)>e^{n^{1/(20\mathsf{depth}(\mathcal{C}''_n))}}\ .$$

This is a contradiction to (49). □

7 Amplification of the classical–quantum gap

In this section, we prove an amplified version of Theorem 6.7 with arbitrary quantum completeness and classical soundness parameters $\mu,\nu\in(0,1)$ satisfying $\nu<1-\mu$. The amplified theorem is obtained by replacing the underlying gate-teleportation relation by its constant-fold parallel repetition.

More precisely, we consider N-fold parallel repetition of the single-qubit gate teleportation problem, denoted by $R^{(N)}_{U_n^{\mathsf{Telep}}}$. We formalize the repeated relation by replacing single-qubit Cliffords and Paulis with simple N-fold tensor products of single-qubit Cliffords and Paulis, respectively.

For each $N\in\mathbb{N}$, let

$$\mathsf{Cliff}^{\otimes N}:=\left\{\bigotimes_{i=1}^{N}C_i\ \middle|\ C_i\in\mathsf{Cliff}\right\}\qquad\text{and}\qquad\mathsf{Pauli}^{\otimes N}:=\left\{\bigotimes_{i=1}^{N}P_i\ \middle|\ P_i\in\mathsf{Pauli}\right\}$$

denote the sets of N-fold tensor products of single qubit Cliffords and single qubit Paulis, respectively.

The relation problem $R^{(N)}_{U_n^{\mathsf{Telep}}}$ has input set

$$\left(\mathsf{Cliff}^{\otimes N}\right)^n = \left\{(C_0^{(N)}, \ldots, C_{n-1}^{(N)}) \mid C_j^{(N)} \in \mathsf{Cliff}^{\otimes N} \text{ for any } j \in \mathbb{Z}_n\right\}$$

and output set

$$\left(\mathsf{Pauli}^{\otimes N}\right)^n = \left\{(P_0^{(N)}, \ldots, P_{n-1}^{(N)}) \mid P_j^{(N)} \in \mathsf{Pauli}^{\otimes N} \text{ for any } j \in \mathbb{Z}_n\right\} .$$

The relation

$$R^{(N)}_{U_n^{\mathsf{Telep}}} \subset \left(\mathsf{Cliff}^{\otimes N}\right)^n \times \left(\mathsf{Pauli}^{\otimes N}\right)^n$$

is defined by

$$\big((C_0^{(N)}, \ldots, C_{n-1}^{(N)}), (P_0^{(N)}, \ldots, P_{n-1}^{(N)})\big) \in R^{(N)}_{U_n^{\mathsf{Telep}}} \quad \text{if and only if} \quad \operatorname{tr}\left(P_{n-1}^{(N)} C_{n-1}^{(N)} \cdots P_0^{(N)} C_0^{(N)}\right) \neq 0 .$$

By the mixed-product rule and multiplicativity of the trace over tensor product, this tensor-product formulation is, under canonical identifications $\mathsf{Cliff}^N \cong \mathsf{Cliff}^{\otimes N}$ and $\mathsf{Pauli}^N \cong \mathsf{Pauli}^{\otimes N}$, equivalent to the N-fold parallel repetition of the single-qubit gate teleportation problem. Explicitly, let $\pi_i(\otimes_{j=1}^N P_j) = P_i$ denote the canonical projection onto the i-th Pauli in $\mathsf{Pauli}^{\otimes N}$. We use an analogous definition for projections of elements of $\mathsf{Cliff}^{\otimes N}$. Then we have

$$\big((C_0^{(N)}, \ldots, C_{n-1}^{(N)}), (P_0^{(N)}, \ldots, P_{n-1}^{(N)})\big) \in R^{(N)}_{U_n^{\mathsf{Telep}}}$$

if and only if

$$\big((\pi_i(C_0^{(N)}), \ldots, \pi_i(C_{n-1}^{(N)})), (\pi_i(P_0^{(N)}), \ldots, \pi_i(P_{n-1}^{(N)}))\big) \in R_{U_n^{\mathsf{Telep}}} \text{ for all } i \in [N] .$$

7.1 Parallel repetition for the single-qubit-control pseudo-telepathy game

Here we use a variant of Ran Raz's [22] parallel repetition theorem for games to obtain a bound on the probability of winning many parallel copies of the magic-square game with random Clifford inputs as described in Section 1.2.

A free two-prover one-round game is a 6-tuple $\mathcal{G} = (\mathcal{A}, \mathcal{B}, \mathcal{X}, \mathcal{Y}, P_A \times P_B, \mathrm{Ver})$, where $\mathcal{A}, \mathcal{B}$ are the finite sets of questions to Alice and Bob, respectively; $\mathcal{X}, \mathcal{Y}$ are the finite sets of answers of Alice and Bob, respectively; $P_A \times P_B$ is a product distribution on $\mathcal{A} \times \mathcal{B}$; and $\mathrm{Ver} : \mathcal{A} \times \mathcal{B} \times \mathcal{X} \times \mathcal{Y} \to \{0, 1\}$ is the verification predicate. Upon receiving questions $\alpha \in \mathcal{A}$ and $\beta \in \mathcal{B}$ and returning answers $x \in \mathcal{X}$ and $y \in \mathcal{Y}$, the players win if and only if $\mathrm{Ver}(\alpha, \beta, x, y) = 1$.

Theorem 7.1 (Parallel repetition for free games [2])**.** *Let $\mathcal{G} = (\mathcal{A}, \mathcal{B}, \mathcal{X}, \mathcal{Y}, P_A \times P_B, \mathrm{Ver})$ be a free two-prover one-round game. Assume that $0 < \varepsilon < 1/2$ and that every deterministic classical strategy for $\mathcal{G}$ given by a pair (f, g) of functions $f : \mathcal{A} \to \mathcal{X}$ and $g : \mathcal{B} \to \mathcal{Y}$ has winning probability at most $1 - \varepsilon$, i.e.,*

$$\Pr_{(\alpha, \beta) \sim P_A \times P_B}[\mathrm{Ver}(\alpha, \beta, f(\alpha), g(\beta)) = 1] \leq 1 - \varepsilon .$$

Let $N \geq 1$ and write $\alpha^{(N)} = (\alpha_1, \ldots, \alpha_N) \in \mathcal{A}^N$ and $\beta^{(N)} = (\beta_1, \ldots, \beta_N) \in \mathcal{B}^N$. Then for all functions $F = (F_1, \ldots, F_N) : \mathcal{A}^N \to \mathcal{X}^N$ and $G = (G_1, \ldots, G_N) : \mathcal{B}^N \to \mathcal{Y}^N$, we have

$$\Pr_{(\alpha^{(N)}, \beta^{(N)}) \sim (P_A \times P_B)^N}\Big[\mathrm{Ver}\big(\alpha_i, \beta_i, F_i(\alpha^{(N)}), G_i(\beta^{(N)})\big) = 1 \quad \forall i \in [N]\Big] \leq \left(1 - \frac{\varepsilon^2}{9}\right)^{N/(18 \log_2 |\mathcal{X} \times \mathcal{Y}| + 3)} .$$

We use this theorem to obtain a parallel-repetition bound for a generalized variant of the magic-square game with random Clifford inputs. This result is analogous to Lemma 1.3.

Lemma 7.2. *Let $N \in \mathbb{N}$ and let $F' : \mathsf{Cliff}^{\otimes N} \to \mathsf{Pauli}^{\otimes N}$ and $G' : \mathsf{Cliff}^{\otimes N} \to \mathsf{Pauli}^{\otimes N}$ be arbitrary functions. Then*

$$\Pr_{(U,V)\in\left(\mathsf{Cliff}^{\otimes N}\right)^2}\left[\operatorname{tr}(UF'(U)VG'(V)) = 0\right] \geq 1 - \exp(-N/(75 \cdot 9 \cdot 36^2)) \ ,$$

where U and V are chosen uniformly and independently at random from $\mathsf{Cliff}^{\otimes N}$.

Proof. Using the notation of Theorem 7.1, the single-round game $\mathcal{G}$ analyzed in Lemma 1.3 has the question sets $\mathcal{A} = \mathcal{B} = \mathsf{Cliff}$, answer sets $\mathcal{X} = \mathcal{Y} = \mathsf{Pauli}$ and the distribution of questions $P_A \times P_B$, where both P_A and P_B are the uniform distributions on Cliff. Its verification predicate is

$$\operatorname{Ver}(U, V, P, Q) = 1 \qquad \text{if and only if} \qquad \operatorname{tr}(UPVQ) \neq 0 \ .$$

By Lemma 1.3, every deterministic classical strategy for $\mathcal{G}$ wins with probability at most $1 - \frac{1}{36}$. Hence Theorem 7.1 applies with

$$\varepsilon = \frac{1}{36} \qquad \text{and} \qquad |\mathsf{Pauli} \times \mathsf{Pauli}| = 16 \ .$$

Fix arbitrary functions $F', G' : \mathsf{Cliff}^{\otimes N} \to \mathsf{Pauli}^{\otimes N}$. Since $\mathsf{Cliff}^{\otimes N} \cong \mathsf{Cliff}^N$ and $\mathsf{Pauli}^{\otimes N} \cong \mathsf{Pauli}^N$, the maps F' and G' can be viewed as strategies for the N-fold repeated game. Let $U = U_1 \otimes \ldots \otimes U_N \in \mathsf{Cliff}^{\otimes N}$ and $V = V_1 \otimes \ldots \otimes V_N \in \mathsf{Cliff}^{\otimes N}$. For each $i \in [N]$, let $F'_i = \pi_i \circ F' : \mathsf{Cliff}^{\otimes N} \to \mathsf{Pauli}$ and $G'_i = \pi_i \circ G' : \mathsf{Cliff}^{\otimes N} \to \mathsf{Pauli}$, where $\pi_i(\otimes_{j=1}^N P_j) = P_i$ denotes the canonical projection onto the i-th Pauli. By the mixed-product rule and multiplicativity of the trace over tensor products, we have

$$\operatorname{tr}(UF'(U)VG'(V)) = \prod_{i=1}^{N} \operatorname{tr}\left(U_i F'_i(U) V_i G'_i(V)\right) \ .$$

Therefore

$$\operatorname{tr}(UF'(U)VG'(V)) \neq 0 \qquad \text{if and only if} \qquad \forall i \in [N],\ \operatorname{tr}\left(U_i F'_i(U) V_i G'_i(V)\right) \neq 0 \ .$$

If $U, V \in \mathsf{Cliff}^{\otimes N}$ are drawn uniformly and independently at random, then the corresponding tuples $(U_1, \ldots, U_N)$ and $(V_1, \ldots, V_N)$ are drawn uniformly and independently at random from Cliff^N. Therefore, for the strategies induced by F' and G', the event $\operatorname{tr}(UF'(U)VG'(V)) \neq 0$ is exactly the event that the repeated game is won in every coordinate.

Applying Theorem 7.1 we have

$$\begin{aligned}\Pr_{(U,V)\in\left(\mathsf{Cliff}^{\otimes N}\right)^2}\left[\operatorname{tr}(UF'(U)VG'(V)) \neq 0\right] &\leq \left(1 - \frac{1}{9 \cdot 36^2}\right)^{N/(18\log_2 16 + 3)} \\ &= \left(1 - \frac{1}{9 \cdot 36^2}\right)^{N/75} .\end{aligned}$$

By considering the complementary event, we obtain

$$\begin{aligned}\Pr_{(U,V)\in\left(\mathsf{Cliff}^{\otimes N}\right)^2}\left[\operatorname{tr}(UF'(U)VG'(V)) = 0\right] &\geq 1 - \left(1 - \frac{1}{9 \cdot 36^2}\right)^{N/75} \\ &\geq 1 - \exp(-N/(75 \cdot 9 \cdot 36^2)) \ ,\end{aligned}$$

where we used $1 - x \leq \exp(-x)$ for all $x \in \mathbb{R}$. □

7.2 Hardness amplification for NC^0 and AC^0 circuits

The following theorems establishing hardness amplification for NC^0 circuits are proved by the same arguments as in Sections 3 and 4 after replacing Cliff and Pauli by $\mathsf{Cliff}^{\otimes N}$ and $\mathsf{Pauli}^{\otimes N}$, respectively. This is justified by the fact that $\mathsf{Cliff}^{\otimes N}$ normalizes $\mathsf{Pauli}^{\otimes N}$ and that any two elements of $\mathsf{Pauli}^{\otimes N}$ either commute or anticommute.

Substituting the result of Lemma 7.2 for Lemma 1.3 in Theorem 3.1 we obtain the following result, which can be understood as a strengthening of Theorem 3.1.

Theorem 7.3. *Let $N \in \mathbb{N}$ be a constant. Let $\mathcal{C}_n^{(N)} : \left(\mathsf{Cliff}^{\otimes N}\right)^n \to \left(\mathsf{Pauli}^{\otimes N}\right)^n$ be an NC^0 circuit with gates of fan-in bounded by a constant K, such that*

$$\Pr_{C^{(N)} \in \left(\mathsf{Cliff}^{\otimes N}\right)^n} \left[(C^{(N)}, \mathcal{C}_n^{(N)}(C^{(N)})) \in R^{(N)}_{U_n^{\mathsf{Telep}}}\right] > \exp(-N/(75 \cdot 9 \cdot 36^2)) \ .$$

Then the circuit depth of $\mathcal{C}_n$ satisfies

$$\mathsf{depth}(\mathcal{C}_n) \in \Omega(\log n) \ .$$

We now turn to the restricted problems, which will be used to obtain the AC^0 hardness result below. Replacing Theorem 3.1 by the result of the preceding theorem in the proof of Theorem 4.1 gives the following result, which strengthens Theorem 4.1.

Theorem 7.4. *Let $N \in \mathbb{N}$, $0 < \gamma \leq 1$ and $1/2 < \delta \leq 1$ be constants. Let $\xi : \mathbb{Z}_n \to \mathsf{Cliff}^{\otimes N} \cup \{*\}$ be a restriction that keeps $m \geq \gamma n^\delta$ inputs active. Then any NC^0-circuit $\mathcal{C}_n : \left(\mathsf{Cliff}^{\otimes N}\right)^m \to \left(\mathsf{Pauli}^{\otimes N}\right)^n$ solves the restricted repeated gate-teleportation problem $R^{(N)}_{U_n^{\mathsf{Telep}}} \restriction_\xi$ with average probability at most*

$$\Pr_{D^{(N)} \in \left(\mathsf{Cliff}^{\otimes N}\right)^m} \left[\left(D^{(N)}, \mathcal{C}_n^N(D^{(N)})\right) \in R^{(N)}_{U_n^{\mathsf{Telep}}} \restriction_\xi\right] < \exp(-N/(75 \cdot 9 \cdot 36^2))$$

for an instance $D^{(N)}$ chosen uniformly from the set $\left(\mathsf{Cliff}^{\otimes N}\right)^m$.

As in Sections 4.2 and 4.3, we consider bit-encoded inputs. Applying the same reasoning as before, but replacing the 5-bit encoding of Cliff by the $5N$-bit encoding of $\mathsf{Cliff}^{\otimes N}$. We first prove the following analogue of Lemma 4.4.

Lemma 7.5. *Let $N > 0$ be an integer. Let $\mathsf{Enc}^{(1)}, \mathsf{Enc}^{(2)} \in \mathcal{E}_N$ be valid encodings (analogous to the ones described in Section 4.2). Let $F : \{0,1\}^{5N} \to \mathsf{Pauli}^{\otimes N}$ and $G : \{0,1\}^{5N} \to \mathsf{Pauli}^{\otimes N}$ be arbitrary functions. Then*

$$\Pr_{(x,y) \in (\{0,1\}^{5N})^2} \left[\mathrm{tr}\left(\mathsf{Enc}^{(1)}(x) F(x) \mathsf{Enc}^{(2)}(y) G(y)\right) = 0\right] \geq 1 - \exp\left(-N/(32 \cdot 75 \cdot 36^2)\right) \ .$$

Proof. Recall that for the valid encoding maps we fixed an injective map $\iota : \mathsf{Cliff} \to \{0,1\}^5$, so that $\iota(\mathsf{Cliff}) \subset \{0,1\}^5$ is the distinguished set of bit strings encoding Clifford elements.

Let $x = (x_1, \ldots, x_N)$ and $y = (y_1, \ldots, y_N)$ be drawn independently and uniformly from $(\{0,1\}^5)^N$, where $x_i, y_i \in \{0,1\}^5$ for each $i \in [N]$. We call a coordinate i good if $(x_i, y_i) \in (\iota(\mathsf{Cliff}))^2$. Define the set of all good coordinates

$$\mathsf{Good} := \{i \in [N] : (x_i, y_i) \in \iota(\mathsf{Cliff})^2\} \ .$$

Define an indicator random variable

$$I_i = \begin{cases} 1 & \text{if } (x_i, y_i) \in \iota(\mathsf{Cliff})^2 \\ 0 & \text{otherwise.} \end{cases}$$

Then $I_1, \ldots, I_N$ are independent Bernoulli random variables with

$$\Pr_{(x,y)\in(\{0,1\}^{5N})^2}[I_i = 1] = \Pr_{(x,y)\in(\{0,1\}^{5N})^2}[(x_i, y_i) \in (\iota(\mathsf{Cliff}))^2] = \frac{9}{16} \ .$$

Define a random variable

$$K := \sum_{i=1}^{N} I_i$$

and note that $K = |\mathsf{Good}|$. The random variable K is distributed according to the binomial distribution $\mathsf{BIN}(N, 9/16)$. Fix a set $H = \{h_1 < \ldots < h_k\} \subset [N]$ of size $|H| = k$. We use $H^c = [N] \setminus H$ to denote the complement of H. Define the event

$$E_H := \{\mathsf{Good} = H\}$$

(and thus also $K = k$). Let

$$\chi = \Big((a_i, b_i)\Big)_{i\in H^c} \in \big((\{0,1\}^5 \setminus \iota(\mathsf{Cliff}))^2\big)^{N-k}$$

be a fixed assignment of the values of all coordinates in the complement H^c. Define an event

$$E_{H^c,\chi} = \{(x_i, y_i) = (a_i, b_i) \text{ for all } i \in H^c\} \ .$$

Condition on both events E_H and $E_{H^c,\chi}$. Under this conditioning, the random variables

$$U_\ell = \iota^{-1}(x_{h_\ell}) \qquad \text{and} \qquad V_\ell = \iota^{-1}(y_{h_\ell}) \ ,$$

for $\ell \in [k]$, are drawn uniformly and independently from Cliff. Thus

$$U = \bigotimes_{\ell=1}^{k} U_\ell \qquad \text{and} \qquad V = \bigotimes_{\ell=1}^{k} V_\ell \ .$$

are drawn uniformly and independently from $\mathsf{Cliff}^{\otimes k}$. Fixing the values of coordinates in the complement H^c according to χ, and restricting to the tensor factors indexed by H, the functions F and G determine functions

$$F_H, G_H : \mathsf{Cliff}^{\otimes k} \to \mathsf{Pauli}^{\otimes k} \ .$$

Using the Lemma 7.2 we have

$$\Pr\Big[\mathrm{tr}\left(UF_{H,\chi}(U)VG_{H,\chi}(V)\right) = 0 \ \Big|\ E_H \cap E_{H^c,\chi}\Big] \geq 1 - e^{-k/(75\cdot 9\cdot 36^2)} \ . \tag{51}$$

Furthermore, under the conditioning on $E_H \cap E_{H^c,\chi}$ the operator $\mathsf{Enc}^{(1)}(x)F(x)\mathsf{Enc}^{(2)}(y)G(y)$ factorizes as tensor product on coordinates in H and H^c. By multiplicativity of trace over tensor products, the identity $\mathrm{tr}\left(UF_{H,\chi}(U)VG_{H,\chi}(V)\right) = 0$ implies that $\mathrm{tr}\Big(\mathsf{Enc}^{(1)}(x)F(x)\mathsf{Enc}^{(2)}(y)G(y)\Big) = 0$. With Eq. (51), this implies that for every subset $H \subset [N]$ of size $|H| = k$ and every $\chi \in \big((\{0,1\}^5 \setminus \iota(\mathsf{Cliff}))^2\big)^{N-k}$ we have

$$\Pr\Big[\mathrm{tr}\Big(\mathsf{Enc}^{(1)}(x)F(x)\mathsf{Enc}^{(2)}(y)G(y)\Big) = 0 \ \Big|\ E_H \cap E_{H^c,\chi}\Big] \geq 1 - e^{-k/(75\cdot 9\cdot 36^2)} \ .$$

Now consider the random subset $\mathsf{Good} \subset [N]$ defined above. Let E denote the event

$$E := \Big\{\mathrm{tr}\Big(\mathsf{Enc}^{(1)}(x)F(x)\mathsf{Enc}^{(2)}(y)G(y)\Big) = 0\Big\} \ .$$

By the law of total probability we have

$$\Pr[E \mid K=k] = \sum_{\substack{H\subset[N]\\|H|=k}} \sum_{\chi\in((\{0,1\}^5\setminus\iota(\mathsf{Cliff}))^2)^{N-k}} \Pr[E_H\cap E_{H^c,\chi} \mid K=k]\Pr[E \mid E_H\cap E_{H^c,\chi}]$$
$$\geq 1-e^{-k/(75\cdot 9\cdot 36^2)} \tag{52}$$

for every $k\in\{0,\ldots,N\}$. Finally, by averaging over k (according to the binomial distribution), using the bound (52) obtained above, and interpreting the sum as an expectation, we obtain

$$\begin{aligned}
\Pr_{(x,y)\in(\{0,1\}^{5N})^2}\left[\mathrm{tr}\left(\mathsf{Enc}^{(1)}(x)F(x)\mathsf{Enc}^{(2)}(y)G(y)\right)=0\right] &= \sum_{k=0}^{N}\Pr[E \mid K=k]\Pr[K=k]\\
&\geq \sum_{k=1}^{N}\left(1-e^{-k/(75\cdot 9\cdot 36^2)}\right)\Pr[K=k]\\
&= 1-\mathbb{E}[e^{-K/(75\cdot 9\cdot 36^2)}]\\
&= 1-\left(1-\frac{9}{16}\left(1-e^{-1/(75\cdot 9\cdot 36^2)}\right)\right)^N\\
&\geq 1-\exp\left(-N\frac{9}{16}\left(1-e^{-1/(75\cdot 9\cdot 36^2)}\right)\right)\\
&\geq 1-\exp(-N/(32\cdot 75\cdot 36^2))\ ,
\end{aligned}$$

where we used the binomial theorem to obtain the equality on the fourth line and $1-x\leq e^{-x}$ and $1-e^{-x}\geq x/2$ for all $x\in[0,1]$ to obtain the last two inequalities. □

By substituting the result of Lemma 7.5 for Lemma 4.4 in the proof of Theorem 4.5 we obtain the following theorem.

Theorem 7.6. *For any $N\in\mathbb{N}$. Let $0<\gamma\leq 1$ and $1/2<\delta\leq 1$ be constants. Let $\mathsf{Enc}=(\mathsf{Enc}^{(0)},\ldots,\mathsf{Enc}^{(n-1)})\in(\mathcal{E}_N)^n$ be an n-tuple of valid block-encoding maps*

$$\mathsf{Enc}^{(i)}:\{0,1\}^{5N}\to\mathsf{Cliff}^{\otimes N}\qquad \textit{for}\ \ i\in\mathbb{Z}_n$$

(analogous to the ones described in Section 4.2). Let $\xi:\mathbb{Z}_n\to\{0,1\}^{5N}\cup\{*\}$ be a restriction that keeps $m\geq\gamma n^\delta$ inputs active. Assume that the set of active inputs is $\xi^{-1}(\{*\})=\{i_0<\ldots<i_{m-1}\}$. Then any NC^0 circuit $\mathcal{C}^{(N)}:(\{0,1\}^{5N})^m\to\left(\mathsf{Pauli}^{(N)}\right)^n$ satisfies

$$\Pr_{x\in(\{0,1\}^M)^m}\left[(\widehat{\mathsf{Enc}}(x),\mathcal{C}^{(N)}(x))\in R^{(N)}_{U_n^{\mathsf{Telep}}}\restriction_{\mathsf{Enc}(\xi)}\right]<\exp\left(-N/(32\cdot 75\cdot 36^2)\right)$$

for a uniformly randomly chosen input $x=(x^{(0)},\ldots,x^{(m-1)})\in(\{0,1\}^{5N})^m$, where we introduced

$$\widehat{\mathsf{Enc}}(x)=(\mathsf{Enc}^{(i_0)}(x^{(0)}),\ldots,\mathsf{Enc}^{(i_{m-1})}(x^{(m-1)}))\in\left(\mathsf{Cliff}^{\otimes N}\right)^m\qquad \textit{for}\qquad x\in(\{0,1\}^{5N})^m\ .$$

The following result establishing hardness amplification for AC^0-circuits follows by the same arguments as in Section 5. There, the switching lemma was applied with block-restriction using blocks of 5 bits to encode Clifford inputs and blocks of 2 bits to encode Pauli outputs. In the present setting, these blocks are replaced by blocks of $5N$ bits encoding elements of $\mathsf{Cliff}^{\otimes N}$ and blocks of $2N$ bits encoding elements of $\mathsf{Pauli}^{\otimes N}$. Together with Theorem 7.6, this yields the following.

Theorem 7.7. *Let $N \in \mathbb{N}$ be a constant. Let $\mathsf{Enc} \in \mathcal{E}_N^n$ be an arbitrary n-tuple of valid encoding maps (analogous to the ones described in Section 4.2). Then, for all sufficiently large n, every non-uniform AC^0 circuit $\mathcal{C}_n^{(N)} : (\{0,1\}^{5N})^n \to (\mathsf{Pauli}^{\otimes N})^n$ of depth d satisfying*

$$\Pr_{x \in (\{0,1\}^{5N})^n}[(\mathsf{Enc}(x), \mathcal{C}_n^{(N)}(x)) \in R^{(N)}_{U_n^{\mathsf{Telep}}}] \geq 2\exp\left(-N/(32 \cdot 75 \cdot 36^2)\right) ,$$

has size at least $e^{(nN)^{1/(20Nd)}}$.

This theorem can be understood as a strengthening of Theorem 5.3.

Proof. The proof is identical to that of Theorem 5.3, with the following two modifications. First, we use the analogue of Theorem 5.2 in which the input blocks have size $5N$ bits rather than 5 bits. Second, we invoke Theorem 7.6 in place of Theorem 4.5.

Assume, for the sake of contradiction, that there exists an n-tuple $\mathsf{Enc} \in \mathcal{E}_N^n$ of valid encoding maps and an AC^0 circuit $\mathcal{C}_n^{(N)} : (\{0,1\}^{5N})^n \to (\mathsf{Pauli}^{\otimes n})^N$ of depth $d = \mathsf{depth}(\mathcal{C}_n)$ and $\mathsf{size}(\mathcal{C}_n) = s \leq e^{(nN)^{1/(20dN)}}$ such that

$$\Pr_{x \in (\{0,1\}^{5N})^n}[(\mathsf{Enc}(x), \mathcal{C}_n^{(N)}(x)) \in R^{(N)}_{U_n^{\mathsf{Telep}}}] \geq 2\exp\left(-N/(32 \cdot 75 \cdot 36^2)\right) .$$

To obtain this $5N$-bit block version, we repeat the proof of Theorem 5.2 with the following parameter substitutions:

$$n' = 5Nn , \qquad m' = 2Nn , \qquad s \leq e^{(Nn)^{1/(20Nd)}} , \qquad q = 20Nd , \qquad t = np_*^{5N}/5$$

in place of

$$n' = 5n , \qquad m' = 2n , \qquad s \leq e^{n^{1/(20d)}} , \qquad q = 20d , \qquad t = np_*^5/5 ,$$

respectively. Thus we obtain a probability distribution P over block restrictions $\xi^{\mathsf{block}} : \mathbb{Z}_n \to \{0,1\}^{5N} \cup \{*^{\mathsf{block}}\}$, where $*^{\mathsf{block}}$ denotes a free block of $5N$ bits, with the following properties. Condition (iii) below holds for P, and a random restriction $\xi^{\mathsf{block}} \sim P$ satisfies conditions (i) and (ii) with probability at least

$$1 - \exp\left(-c_1(d,N)n^{1/2}\right) ,$$

where $c_1(d,N) > 0$ depends only on d and N.

(i) The number of active blocks satisfies $N(\xi) \geq c_2(d,N)n^{3/4}$, where $c_2(d,N) > 0$ is a constant depending only on d and N.

(ii) The block restricted circuit $\mathcal{C}_n^{(N)} \restriction_{\xi^{\mathsf{block}}}$ can be implemented by an NC^0 circuit of depth $20Nd$.

(iii) For a restriction $\xi^{\mathsf{block}} \sim P$ drawn according to the distribution P the following holds. For any subset $S \subset \mathbb{Z}_n$, the distribution of ξ^{block} conditioned on $S = (\xi^{\mathsf{block}})^{-1}(\{*^{\mathsf{block}}\})$ is uniform over all block restrictions $\tilde{\xi}^{\mathsf{block}} : \mathbb{Z}_n \to \{0,1\}^{5N} \cup \{*^{\mathsf{block}}\}$ having S as the set of active blocks, i.e., $S = (\tilde{\xi}^{\mathsf{block}})^{-1}(\{*^{\mathsf{block}}\})$.

The remainder of the proof is identical to the part of the proof of Theorem 5.3 following the application of Theorem 5.2. Using the $5N$-bit block analogue of Theorem 5.2 above and Theorem 7.6 in place of Theorem 4.5, we obtain

$$\begin{aligned}\Pr_{x \in (\{0,1\}^{5N})^n}[(\mathsf{Enc}(x), \mathcal{C}_n^{(N)}(x)) \in R^{(N)}_{U_n^{\mathsf{Telep}}}] \leq &\exp(-c_1(d,N)n^{1/2}) \\ &+ (1 - \exp(-c_1(d,N)n^{1/2}))\exp\left(-N/(32 \cdot 75 \cdot 36^2)\right) \\ < &2\exp\left(-N/(32 \cdot 75 \cdot 36^2)\right) ,\end{aligned}$$

for all sufficiently large n. This contradicts the assumed lower bound. □

By using an analogue of Lemma 4.3, in which the input blocks have size $5N$ bits rather than 5 bits, we obtain the following corollary.

Corollary 7.8. *Let $N \in \mathbb{N}$ be a constant. For all sufficiently large n, the following holds. Suppose $\mathcal{C}_n^{(N)} : (\mathsf{Cliff}^{\otimes N})^n \to (\mathsf{Pauli}^{\otimes N})^n$ is a depth-d AC^0-circuit solving the single-qubit gate-teleportation problem with success probability at least $3\exp\left(-N/(32 \cdot 75 \cdot 36^2)\right)$ over uniformly random instances. That is,*

$$\Pr_{C^{(N)} \in (\mathsf{Cliff}^{\otimes N})^n}\left[\left(C^{(N)}, \mathcal{C}_n^{(N)}(C^{(N)})\right) \in R^{(N)}_{U_n^{\mathsf{Telep}}}\right] \geq 3\exp\left(-N/(32 \cdot 75 \cdot 36^2)\right) \ .$$

Then

$$\mathsf{size}(\mathcal{C}_n^{(N)}) > e^{(nN)^{1/(20Nd)}} \ .$$

Corollary 7.8 strengthens Corollary 5.4: The classical soundness parameter ν can be made arbitrarily small by a suitable choice of N.

7.3 Amplified noise-resilient quantum advantage against AC^0 with $3D$-local architecture

The N-fold single qubit gate teleportation problem $R^{(N)}_{U_n^{\mathsf{Telep}}} \subset (\mathsf{Cliff}^{\otimes N})^n \times (\mathsf{Pauli}^{\otimes N})^n$ can be solved deterministically by applying N copies of the circuit U_n^{Telep} from Supplementary Figure 6a in parallel. We denote the resulting constant depth circuit by $U_{n,N}^{\mathsf{Telep}}$. As in Section 6, we follow Ref. [6] and apply fault tolerance in the "non-standard way" to obtain quantum advantage with constant-depth noisy $3D$-local quantum circuits. That is, by applying Theorem 6.3, we obtain the following result, which is a "parallel repetition"-version of Theorem 6.4.

Theorem 7.9. *There exists a universal constant threshold $p_{thres} > 0$ such that for every constant $\mu \in (0,1)$ the following holds. For every $n, N \in \mathbb{N}$, let $U_{n,N}^{\mathsf{Telep}} = (U_n^{\mathsf{Telep}})^{\otimes N}$ be the N-fold parallel repetition of the single-qubit gate-teleportation circuit U_n^{Telep} on n qubits. Then an associated fault-tolerant circuit U^{ext} can be realized in a $3D$-local manner using a total of $O(Nn\mathsf{poly}(\log(Nn)))$ qubits. Assuming that copies of the input bits $b \in (\{0,1\}^{5N})^n$ are provided in the relevant locations, the associated interaction graph is obtained by stacking N-layers of Colosseum-shaped $3D$ manifolds on top of each other, see Supplementary Figure 13. For every $p < p_{thres}$ and every noisy implementation of U^{ext} with error strength p, the input-output pair (b, z) obtained by post-processing the measurement result (s, y) for input $b \in (\{0,1\}^{5N})^n$ according to (45) satisfies*

$$\Pr\left[(b, z) \in R^{(N)}_{U_n^{\mathsf{Telep}}}\right] > 1 - \mu \ .$$

By applying Theorem 6.5 with Corollary 7.8 (instead of Corollary 5.4), we obtain the following amplified variant of Theorem 6.7.

Theorem 7.10 ((Sub)exponential quantum advantage with arbitrarily large classical–quantum gap)**.** *There exists a universal threshold $p_{thres} > 0$ such that the following holds for every pair of constants $\mu, \nu \in (0,1)$ with $\nu < 1 - \mu$. There exists a constant $N \in \mathbb{N}$ depending only on ν, functions $n_i, n_o : \mathbb{N} \to \mathbb{N}$ with $n_o(n) \in \Theta(n\mathsf{poly}(\log n))$, a relation*

$$\tilde{\mathcal{R}}_n^{(N)} \subset \{0,1\}^{n_i(n) \cdot N} \times \{0,1\}^{n_o(n) \cdot N} \ ,$$

a set of instances $\tilde{S}_n^{(N)} \subset \{0,1\}^{n_i(n) \cdot N}$, and a constant $\delta(N) > 0$ depending only on N such that for all sufficiently large n, we have the following:

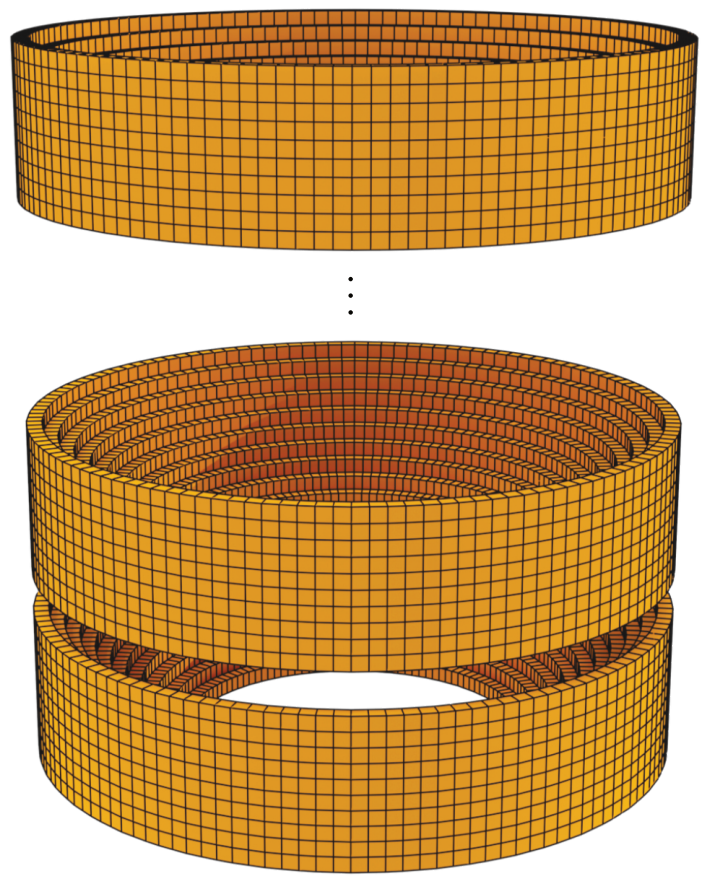

Supplementary Figure 13: The N-layer stacked Colosseum-shaped embedding in $\mathbb{R}^3$ of the interaction graph of the fault-tolerant quantum circuit used for the amplified relation problem. Notice that there is no interaction between the individual layers.

(i) There is a classically controlled, N-layer stacked Colosseum-shaped (Supplementary Figure 13), $3D$-local constant-depth quantum circuit $\tilde{U}$ such that for any noisy implementation of $\tilde{U}$ with error strength $p < p_{thres}$, the output $o \in \{0,1\}^{n_o(n)\cdot N}$ produced by measurement on an arbitrary input $i \in \tilde{S}_n^{(N)}$ satisfies $(i, o) \in \tilde{\mathcal{R}}_n^{(N)}$ with probability at least $1 - \mu$.

(ii) Suppose $\mathcal{C}$ is an AC^0-circuit which – with an average probability greater than ν over a uniformly chosen instance $i \in \tilde{S}_n^{(N)}$ – produces an output $o \in \{0,1\}^{n_o(n)\cdot N}$ such that $(i, o) \in \tilde{\mathcal{R}}_n^{(N)}$. Then its size is at least $\mathsf{size}(\mathcal{C}) > e^{(nN)^{1/(\delta(N)^{\mathsf{depth}(\mathcal{C})})}}$.

Supplementary References

[1] Miklós Ajtai. Σ_1^1-Formulae on finite structures. Ann. Pure Appl. Log., 24:1–48, 1983.

[2] Boaz Barak, Anup Rao, Ran Raz, Ricky Rosen, and Ronen Shaltiel. Strong parallel repetition theorem for free projection games. In Irit Dinur, Klaus Jansen, Joseph Naor, and José Rolim, editors, Approximation, Randomization, and Combinatorial Optimization. Algorithms and Techniques, pages 352–365, Berlin, Heidelberg, 2009. Springer Berlin Heidelberg.

[3] Paul Beame. A switching lemma primer. Technical report, Technical Report UW-CSE-95-07-01, Department of Computer Science and Engineering, University of Washington, 1994.

[4] Adam Bene Watts, Robin Kothari, Luke Schaeffer, and Avishay Tal. Exponential separation between shallow quantum circuits and unbounded fan-in shallow classical circuits. In Proceedings of the 51st Annual ACM SIGACT Symposium on Theory of Computing, STOC 2019, page 515–526, New York, NY, USA, 2019. Association for Computing Machinery.

[5] Héctor Bombín. Single-shot fault-tolerant quantum error correction. Physical Review X, 5(3):031043, 2015.

[6] Sergey Bravyi, David Gosset, Robert König, and Marco Tomamichel. Quantum advantage with noisy shallow circuits. Nature Physics, 16(10):1040–1045, 2020.

[7] Sergey Bravyi, David Gosset, and Robert König. Quantum advantage with shallow circuits. Science, 362(6412):308–311, 2018.

[8] Sergey B Bravyi and A Yu Kitaev. Quantum codes on a lattice with boundary, 1998. arXiv:9811052.

[9] Libor Caha, Xavier Coiteux-Roy, and Robert Koenig. Single-qubit gate teleportation provides a quantum advantage. Quantum, 8:1548, December 2024.

[10] A Robert Calderbank and Peter W Shor. Good quantum error-correcting codes exist. Physical Review A, 54(2):1098, 1996.

[11] Omar Fawzi, Antoine Grospellier, and Anthony Leverrier. Constant overhead quantum fault-tolerance with quantum expander codes. In 2018 IEEE 59th Annual Symposium on Foundations of Computer Science (FOCS), pages 743–754. IEEE, 2018.

[12] Austin G Fowler. Proof of finite surface code threshold for matching. Physical review letters, 109(18):180502, 2012.

[13] Merrick Furst, James B. Saxe, and Michael Sipser. Parity, circuits, and the polynomial-time hierarchy. Mathematical systems theory, 17(1):13–27, December 1984.

[14] Daniel Gottesman. Fault-tolerant quantum computation with constant overhead. Quantum Info. Comput., 14(15–16):1338–1372, nov 2014.

[15] Daniel Grier, Nathan Ju, and Luke Schaeffer. Interactive quantum advantage with noisy, shallow clifford circuits, 2021. arXiv:2102.06833.

[16] Johan Håstad. Almost Optimal Lower Bounds for Small Depth Circuits. In Proceedings of the Eighteenth Annual ACM Symposium on Theory of Computing, STOC '86, page 6–20, New York, NY, USA, 1986. Association for Computing Machinery.

[17] Aleksander Kubica, Beni Yoshida, and Fernando Pastawski. Unfolding the color code. New Journal of Physics, 17(8):083026, 2015.

[18] David Mermin. Extreme quantum entanglement in a superposition of macroscopically distinct states. Physical Review Letters, 65(15):1838, 1990.

[19] Jonathan E Moussa. Transversal clifford gates on folded surface codes. Physical Review A, 94(4):042316, 2016.

[20] Asher Peres. Incompatible results of quantum measurements. Physics Letters A, 151(3):107–108, 1990.

[21] Robert Raussendorf, Sergey Bravyi, and Jim Harrington. Long-range quantum entanglement in noisy cluster states. Physical Review A, 71(6):062313, 2005.

[22] Ran Raz. A parallel repetition theorem. In Proceedings of the Twenty-Seventh Annual ACM Symposium on Theory of Computing, STOC '95, page 447–456, New York, NY, USA, 1995. Association for Computing Machinery.

[23] Benjamin Rossman. An entropy proof of the switching lemma and tight bounds on the decision-tree size of AC0. 2017. `http://www.math.toronto.edu/rossman/logsize.pdf`.

[24] Johan Håstad. Computational limitations for small-depth circuits. PhD thesis, MIT, 1987.

[25] Andrew Steane. Multiple-particle interference and quantum error correction. Proceedings of the Royal Society of London. Series A: Mathematical, Physical and Engineering Sciences, 452(1954):2551–2577, 1996.

[26] Andrew Chi-Chih Yao. Separating the Polynomial-time Hierarchy by Oracles. In 26th Annual Symposium on Foundations of Computer Science (SFCS 1985), pages 1–10, 1985.
