# Supplementary material for: 3D-local noisy shallow quantum circuits defeat unbounded fan-in classical circuits
Source: Nat Commun. 2026 Aug 11;17:8174. doi: 10.1038/s41467-026-76560-x (PMC13463102; doi:10.1038/s41467-026-76560-x)
Supplement: Supplementary file 2 [file 41467_2026_76560_MOESM2_ESM.pdf]

nature portfolio

Peer Review File

3D-local noisy shallow quantum circuits defeat unbounded fan-in classical circuits

Corresponding Author: Dr Libor Caha

This file contains all reviewer reports in order by version, followed by all author rebuttals in order by version.

Version 0:

Reviewer comments:

Reviewer #1

(Remarks to the Author)
Finding problems — regardless of their day-to-day usefulness — where noisy and geometrically constrained quantum computers can outperform their classical counterparts is a hot topic in theoretical computer science. This paper introduces such a problem that the authors prove can be solved with a noisy, low (constant) depth polynomial size, and geometrically constrained quantum circuit; however, this problem cannot be solved by any low (constant) depth polynomial size classical circuit - even one featuring gates with unbounded fan-in (i.e. AC^0).

The problem introduced in this paper is related to pseudo telepathy games, which are games where two players receive independent inputs x_1, x_2 and then must produce outputs y_1, y_2 such that the tuple (x_1, x_2, y_1, y_2) satisfies some relation R to win the game. The players cannot communicate with one another; however, they are able to share an entangled quantum state before the start of the game. These games are called “pseudo telepathy” as access to this shared entangled state allows the players to win the game with probability 1 whereas any strategy without shared entanglement would have a success probability p strictly smaller than 1.

One can use pseudo telepathy games to prove a gap between constant depth quantum and classical circuits by designing a problem where the inputs represent those in a pseudo telepathy game and the outputs represent many outputs of such games. Then the limited depth (and number of gates) available to a classical circuit imply that some of these outputs must be trying to solve the game without any communication, which means these outputs can only be correct with probability at most p. This problem does not hold for low depth quantum circuits, as they can still produce the correct output with probability 1 without communication by using entanglement.

This result builds on ideas from some previously published constructions:

In [1] the authors constructed a similar problem that features a similar gap between quantum and classical circuits; however, their classical lower bound only applies to circuits where the gates have constant fan-in (NC^0 circuits). Thus this work can be seen as an extension of [1] to work against classical circuits with unbounded fan-in (AC^0 circuits). The authors are able to extend their work to this context by using switching lemmas that convert AC^0 circuits into NC^0 circuits when some of the inputs have fixed assignments.

In [2] the authors use a similar switching lemma to construct a different problem where they can prove that (non noisy) constant depth quantum circuits can solve the problem but constant depth classical circuits with unbounded fan-in (AC^0) cannot. The authors in [2], however, do not address the locality or noise constraints addressed in this paper. It is unclear to me how easy it would be to add these constraints to the quantum algorithm for the problem posed in [2].

This work seems like an important step toward a realizable quantum advantage. However, I have a couple of questions for the authors that I would like to see clarified:

1. In theorem 1.1 (i) you state that “Any AC^0-circuit solving the problem with probability at least 0.9888 on average over a randomly chosen instance has superpolynomial size.” Can this value be made smaller by requiring solutions to multiple independent instances of the problem?
2. What makes your problem more amenable to a hardness result against classical AC^0 circuits than that of [1]?

3. Is it a priori obvious that the quantum algorithm in [2] cannot be made geometrically local and error correcting?
4. Can your construction be modified to work on qubits arranged in a 2D lattice or a line?

In addition, I do not support using the word 'colossal advantage' in the title: while this is intended to be a refrence to the shape of their 3d archetecture, it implies a much more significant gap between quantum and classical circuits than they are able to show.

[1] Sergey Bravyi, David Gosset, Robert König and Marco Tomamichel, Quantum advantage with noisy shallow circuits
[2] Adam Bene Watts, Robin Kothari, Luke Schaeffer and Avishay Tal, Exponential Separation between Shallow Quantum Circuits and Unbounded Fan-In Shallow Classical Circuits

Reviewer #2

(Remarks to the Author)
Result:

The authors extend a line of work of unconditional search separations between quantum and classical circuits. They introduce a search problem that can be solved by a constant-depth bounded fan-in quantum circuit (QNC0) that is geometrically local in 3D, even in the presence of noise. On the other hand, they prove hardness against classical AC0 circuits of polynomial size.

Context:

It was previously known that these noisy 3D QNC0 circuits can solve search problems that cannot be solved by classical constant-depth bounded fan-in (NC0) [BGKT20], and that noiseless QNC0 can solve problems that AC0 cannot [BKST19]. The authors also note that by combining previous results [BKST19] and [GJS21], one can also see that noisy QNC that is not necessarily 3D-local can solve problems not in AC0. Thus, their main result advances the state-of-the-art by improving this separation for 3D-local quantum circuits, which the authors argue is necessary for practical implementations on quantum devices.

Key insights I takeaway:

Their problem construction and quantum algorithm is an elegant generalization of that used in [BGKT20] based on the magic square game. They distill the key machinery used in previous shallow circuit separations. Typical quantum circuits used in these shallow circuit separations can be stated as a bunch of Bell pairs, some classically-controlled Clifford gates, and Bell basis measurements. The authors observe that the probability of any particular measurement outcome can be written as the trace of a sequence of single-qubit Clifford and Pauli gates. Exploiting this observation, the authors strengthen the result of [BGKT20] with a simpler proof. These nice ideas are in Lemma 2.3 the proof of thm 4.1.

Techniques:

Before dealing with the noisy setting, they first show a separation between QNC0 and AC0. For this, they introduce a more general version of the 1D magic square problem used by [BGKT20]. Their QNC0 circuit construction characterizes this definition. Proving classical hardness follows the following steps:

- they first show hardness for NC0
- then they show that even if we restrict the problem inputs, the problem is still hard for NC0 (when there's still n^c inputs that are still free, c\in (0,1]).
- next, they show, using the multi-switching lemma from [BKST19] that there's a distribution over random restrictions under which with high probability an AC0 circuit becomes an NC0 circuit, still with n^c free inputs.
- AC0 hardness follows from connecting the previous two points.

Then, to show a separation in the presence of noise, they make use of previous works. They follow the argument used in [BGKT20] to construct a noise-robust version of the problem statement. Combined with a lemma in [GJS21] this completes their argument.

Decision: accept
This result concretely advances our known unconditional separations between quantum and classical computation, by improving the best-known separation to one where the quantum circuit is geometrically local. This makes the quantum algorithm more feasible to actually implement on a quantum device. Their techniques generalizing the magic square game are particularly nice in that they generalize some previous works in this area.

Reviewer #3

(Remarks to the Author)
This paper makes a significant addition to the string of works proving unconditional separations between low-depth quantum and classical circuits. While main driver in this setting is the strength of the separation, other considerations, such as noise-resilience and locality, are of interest; this work shows a separation between noisy 3D-local QNC^0 and AC^0, a combination that was not previously known. There techniques are borrowed and adapted from prior work.

I have verified the arguments and calculations (mainly up up to section 6, inclusive) and found no reason for concern regarding the correctness of the results. I do have some concerns regarding section 7, particularly with respect to the amount of detail of proofs in that compared to previous sections.

** High-level/general comments **

The paper is generally well-written, with detailed proofs that are easy to verify. However, my impression is that the effort and detail are not dedicated uniformly or according to the importance of each result; more to the point, the separations shown in Sections 1-6 were previously known (albeit the new results extend to the noisy 3D case, which is not the case for prior separations against AC^0) and take 37 pages, while arguably the main contribution, Section 7, is much less formal -- indeed, most of the section reads like a technical overview where the formal content is concentrated on pages 46-49. The section would greatly benefit from a formal treatment at the same level as the previous ones, e.g., by stating the relevant definitions in definition environments, clearly stating the results derived from previous work and omitting what does not directly apply. (More details follow.)

** Lower-level/detailed comments **

- Section 4.1: the main goal seems to be to extend the authors' earlier result [9] to the average case -- which is most relevant not for its own sake, but because it is amenable to the AC^0 hardness proved later on. It would be useful to make the connection both to [9] and to the future sections explicitly: e.g., what exactly does Corollary 4.2 yield that is new compared to [9]? The key techniques/tools look very similar to those of [9] (namely, Pauli conjugation and light-cone arguments), so it is not immediately clear what is new. In particular, the proof of Theorem 4.1 is quite long and seems like a simple extension of prior work; if so, it could be moved to an appendix. (And if not, the novelty should be made explicit.)

- Lemma 5.2: The last inequality is the only part where the assumption \delta < 1/2 is needed; it is worth pointing out.

- Section 5.2: the issue of "compatibility" of random restrictions to problems whose domain size is not a power of 2 is technically important, but in my view distracts from the main message of the paper; a reasonable alternative is to treat it accordingly and move proofs to an appendix, only keeping the formal versions of statements of the sort "representing Cliffords by bit strings makes no material difference" in the body. Moreover, it's not entirely clear why the proof of Lemma 5.3 makes use of random encodings: can't one choose the images of the 32^n - 24^n "leftover" strings so that the fraction of (Enc(x), F \circ Enc(x)) is close to the fraction of (C, F(C))? It seems like this lemma can have a much shorter proof. Note, also, that this proposed simplification and the current proof both assume at least one C is such that (C, F(C)) is not in R (otherwise q = 1 and the last inequality of the lemma fails to hold).

- Section 6.2: properties (i), (ii) and (iii) of the distribution P should be stated either in a definition environment (which is probably best, since it's referred to elsewhere) or in the statement of Theorem 7.2.

- Theorem 7.2:
- Only q and t are chosen parameters but the wording suggests p_* is also "chosen". Moreover, the expression for p_* does not take the constant factor of Lemma 6.1 into account.
- The notation for "NC^0 of depth 20d" is undefined; one could either explicitly define an event or define notation for the set "NC^0 of depth 20d".
- \mathcal{E}(\rho) seems like an odd choice of notation: \mathcal{E} is a set of encodings, and \rho is a random restriction; these symbols are associated to other objects, while \mathcal{E}(\rho) seems to not relate to either in the way the notation suggests. It would be best to choose another, previously unused, symbol.
- The second paragraph of step 2 in the definition of P mentions \eta as "an additional" restriction, but it is not "additional". But more importantly, the case where the set is empty is the low-probability "bad" event; it seems much more explicit to say "do nothing" or "choose an arbitrary restriction" rather than choosing a random one (which suggests there is something interesting happening in this event).

- Figure 10: \eta and \rho are swapped, and the box "Implementable in NC^0" doesn't quite communicate what I think was intended: that C and C restricted by \rho may not be implementable, but C restricted by the concatenation of \rho and \eta (and \tau) is (with high probability).

- Section 7.1 and 7.2: these read like technical overviews; I would strongly suggest separating formal definitions (e.g., local stochastic noise, transversality, single-shot, etc) from discussions (e.g., the sentences starting with "By the CSS-nature..." or the discussion of the subspace to which a measurement outcome x belongs). Generally, here and in the remainder of Section 7 I found it difficult to differentiate formal definitions and results from prior work from high-level discussions.

- Section 7.3:
- The sentence "It is possible..." until the end of the paragraph is contradictory: it states that single-shot state preparation is "not needed" and "key to making the circuit local".
- Quite a few terms/values are undefined: "interaction graph", the faces of the prism (I assume they are the triangular faces, but this isn't immediate), "tesselation by a square lattice", "linear size of the prism", the size L, etc. This section seems like it would greatly benefit from crisp definitions and prior results (in particular, omitting reference to terms/defs that are not immediately relevant for the results that follow). Labeling Figure 11 with some relevant quantities would also help.
- r_in and r_out are not defined; they could be referenced in Figure 12, and it could also refer back to quantities in Figure 11.

The figure also suggests that there's a gap between wedges, which I imagine is not the case (or the embedding would be homeomorphic to a sphere).

** Minor comments/typos **

- Page 1: The sentence "Subexponential..." seems disconnected, and doesn't seem relevant enough to appear in the abstract.
- Page 3:
- "subexponential" usually denotes an upper bound, so the current use is a bit confusing.
- noise strength -> noise rate?
- Page 12: can use \qedhere to align with the expression at the end of Lemma 2.3
- Page 13: the parenthetical in Figure 13 "(i.e., this ... gate.)" is worded oddly; perhaps "they constitute classically controlled Clifford gates"?
- Page 18: A number of C_j and C_k are swapped in items (i) and (ii) (after "This implies the following)
- Page 23: |V_I| \leq -> |V_I| \geq, and can use \qedhere too
- Page 25: Expectation is equal to probability, not just lower bounded by it
- Page 26: \mathbb{Z}^n -> \mathbb{Z}_n (end of 1st paragraph of Section 5.3)
- Page 29: *^\textsf{block} is undefined; perhaps use *^5, or a placeholder symbol other than *?
- Page 30:
- computing output -> computing the output
- \log is used to mean \ln; either convention is fine, but since there are powers of 2 and powers of e in the paper, best to state what it is
- the inputs Clifford -> the input Clifford
- Page 31: The denominator of (32) needs parentheses around d - 1 in the exponent of n in the denominator
- with high probability -> with probability 1 - o(1)? (Maybe best to define earlier on what "whp" means throughout the paper)
- all block restriction -> all block restrictions
- Page 34:
- (37) should have a strict inequality inside the probability
- Since by -> By
- Page 35 and 36: Stating "depth-d AC^0 circuit" in Theorem 6.3 and Corollary 6.4 simplifies the expression for size(C_n)
- Page 37: Corollary 6.5 does not need the definitions in English ("of constant ... quantum circuits" can be omitted). The corollary would also benefit from a discussion of what, if anything, is novel: is the 1D separation against AC^0 already new?
- Page 39:
- what is known about the constants c', c''? Larger than 0? Less than 1? (Likewise for Condition 2' in Page 42.)
- q_th -> q
- \Psi \in (\mathbb{C}^2 -> \ket{\Psi} \in (\mathbb{C}^2
- Page 43: extend circuit -> extended circuit
- Page 44: subset of {0,1}^n. -> subset of {0,1}^n).
- Page 47:
- size D of circuit is undefined
- m_aux-bits -> m_aux-bit
- case, both -> case,
- Page 48: O(exp(polylog(n))) -> exp(polylog(n))

Version 1:

Reviewer comments:

Reviewer #1

(Remarks to the Author)
The authors have adequately addressed my prior concerns about the paper

Reviewer #3

(Remarks to the Author)
The revised manuscript addresses all of the points raised in my earlier review, including comments/highlights that successfully clarify my mistaken interpretation of the importance of each result (i.e., the technical contribution of 1D-local perfect QNC0 advantage against NC0 vs. that of "replacing" the perfect 1D-local circuit by a 3D-local but noise-resilient one). I have no further suggestions other than the (very) minor ones below.

p.1 (of main PDF): "circuits (respectively associated complexity classes)" seems to be missing a connective in the parenthetical: "(and/thus/etc. respectively..."

p. 17 (of supplementary PDF): (obtained by parallel) repetition -> (obtained by parallel repetition)
p. 32: there's an instance of q_{th}, which I assume was meant to be q_{thres}
p. 34: Refs. [12] -> Ref. [12]
p. 42: the product distribution -> a product distribution?

Open Access This Peer Review File is licensed under a Creative Commons Attribution 4.0 International License, which permits use, sharing, adaptation, distribution and reproduction in any medium or format, as long as you give appropriate credit to the original author(s) and the source, provide a link to the Creative Commons license, and indicate if changes were made.
In cases where reviewers are anonymous, credit should be given to 'Anonymous Referee' and the source.
The images or other third party material in this Peer Review File are included in the article's Creative Commons license, unless indicated otherwise in a credit line to the material. If material is not included in the article's Creative Commons license and your intended use is not permitted by statutory regulation or exceeds the permitted use, you will need to obtain permission directly from the copyright holder.
To view a copy of this license, visit https://creativecommons.org/licenses/by/4.0/

We thank the referees for their careful evaluation of our manuscript and for their helpful comments and suggestions. We have revised the manuscript accordingly and have addressed all comments raised. In particular, in response to the open question raised by Reviewer #1, we have added new material on the amplification of the classical–quantum gap, now presented as Section 7 of the Supplementary Information. Below, we provide our point-by-point responses and indicate how each comment has been addressed in the revised manuscript.

Reviewer #1 (Remarks to the Author):

Finding problems — regardless of their day-to-day usefulness — where noisy and geometrically constrained quantum computers can outperform their classical counterparts is a hot topic in theoretical computer science. This paper introduces such a problem that the authors prove can be solved with a noisy, low (constant) depth polynomial size, and geometrically constrained quantum circuit; however, this problem cannot be solved by any low (constant) depth polynomial size classical circuit - even one featuring gates with unbounded fan-in (i.e. AC^0).

The problem introduced in this paper is related to pseudo telepathy games, which are games where two players receive independent inputs x_1, x_2 and then must produce outputs y_1, y_2 such that the tuple (x_1, x_2, y_1, y_2) satisfies some relation R to win the game. The players cannot communicate with one another; however, they are able to share an entangled quantum state before the start of the game. These games are called "pseudo telepathy" as access to this shared entangled state allows the players to win the game with probability 1 whereas any strategy without shared entanglement would have a success probability p strictly smaller than 1.

One can use pseudo telepathy games to prove a gap between constant depth quantum and classical circuits by designing a problem where the inputs represent those in a pseudo telepathy game and the outputs represent many outputs of such games. Then the limited depth (and number of gates) available to a classical circuit imply that some of these outputs must be trying to solve the game without any communication, which means these outputs can only be correct with probability at most p. This problem does not hold for low depth quantum circuits, as they can still produce the correct output with probability 1 without communication by using entanglement.

This result builds on ideas from some previously published constructions:

In [1] the authors constructed a similar problem that features a similar gap between quantum and classical circuits; however, their classical lower bound only applies to circuits where the gates have constant fan-in (NC^0 circuits). Thus this work can be seen as an extension of [1] to work against classical circuits with unbounded fan-in (AC^0 circuits). The authors are able to extend their work to this context by using switching lemmas that convert AC^0 circuits into NC^0 circuits when some of the inputs have fixed assignments.

In [2] the authors use a similar switching lemma to construct a different problem where they can prove that (non noisy) constant depth quantum circuits can solve the problem but constant depth classical circuits with unbounded fan-in (AC^0) cannot. The authors in [2], however, do not address the locality or noise constraints addressed in this paper. It is unclear to me how easy it would be to add these constraints to the quantum algorithm for the problem posed in [2].

This work seems like an important step toward a realizable quantum advantage.

Response: We sincerely thank the referee for their careful reading and positive assessment of our work. We are grateful for their thoughtful and clear summary of the relation between our result and prior work.

However, I have a couple of questions for the authors that I would like to see clarified:

1. *In theorem 1.1 (i) you state that "Any AC^0-circuit solving the problem with probability*

at least 0.9888 on average over a randomly chosen instance has superpolynomial size." Can this value be made smaller by requiring solutions to multiple independent instances of the problem?

Response: We thank the referee for this question. Indeed, this is possible. We address this point in Section 7 of the Supplementary Information (under the new numbering), where we consider multiple independent instances and prove the corresponding amplification result.

Changes: We have included Section 7 in the Supplementary Information (under the new numbering), which addresses amplification of the classical–quantum gap and significantly strengthens our result.

2. *What makes your problem more amenable to a hardness result against classical AC^0 circuits than that of [1]?*

   Response: In [1], the arguments were based on a violation of a bipartite Bell inequality. The considered computational problem had the property that every instance corresponded to a bipartite Bell inequality setting. This allowed to prove a quantum advantage against NC^0-circuits (which by definition cannot violate Bell inequalities by lightcone arguments).

   To prove a quantum advantage against the stronger class of AC^0-circuits, we rely on the technique of random restrictions. For our arguments to work, a random restriction of the original problem has to correspond to a computational problem of similar type, i.e., the random restriction (more precisely: most random restrictions) essentially preserve the hardness of the "remaining" problem (when considering the free input bits).

   For the problem in [1], this is not the case: a random restriction does not necessarily need to correspond to a bipartite Bell scenario. This is in contrast to the problem considered here.

3. *Is it a priori obvious that the quantum algorithm in [2] cannot be made geometrically local and error correcting?*

   Response: The algorithm in Ref. [2] needs a 2D-local circuit in the noiseless case (specifically for generating the poor man's cat states). The noise-resilient embedding technique from [1] generically increases the dimensionality by 2 to produce a local, noise-robust circuit. Hence, naively applying Ref. [1] to the construction of Ref. [2] yields a 4D-local circuit; see Grier, Ju, and Schaeffer, *Interactive quantum advantage with noisy, shallow Clifford circuits*, arXiv:2102.06833.

   It is unclear if different fault-tolerance techniques could lead to a lower-dimensional embedding. If such a construction could be found, this would most likely also improve our work, possibly resulting in a 2D-local noise-resilient quantum advantage proposal.

4. *Can your construction be modified to work on qubits arranged in a 2D lattice or a line?*

Response: See the last question. We don't expect to obtain a fault-tolerance construction on a line, since there are no quantum error-correcting codes with 1D-local stabilizer generators. It is not known whether fault tolerance can be achieved in any 2D circuits.

In addition, I do not support using the word 'colossal advantage' in the title: while this is intended to be a refrence to the shape of their 3d architecture, it implies a much more significant gap between quantum and classical circuits than they are able to show.

Response: In fact, we have some reservations about the traditional use of "exponential" to describe this kind of advantage (to us, this sounds like a more significant gap than what is actually shown, namely a subexponential lower bound). This is why we used an alternative expression together with a comment on the traditional terminology.

Changes: Following the referee's suggestion, we have now eliminated the use of "colossal" and stick to the traditional terminology.

[1] Sergey Bravyi, David Gosset, Robert König and Marco Tomamichel, Quantum advantage with noisy shallow circuits

[2] Adam Bene Watts, Robin Kothari, Luke Schaeffer and Avishay Tal, Exponential Separation between Shallow Quantum Circuits and Unbounded Fan-In Shallow Classical Circuits

Reviewer #2 (Remarks to the Author):

Result: *The authors extend a line of work of unconditional search separations between quantum and classical circuits. They introduce a search problem that can be solved by a constant-depth bounded fan-in quantum circuit (QNC^0) that is geometrically local in 3D, even in the presence of noise. On the other hand, they prove hardness against classical AC^0 circuits of polynomial size.*

Context: *It was previously known that these noisy 3D QNC^0 circuits can solve search problems that cannot be solved by classical constant-depth bounded fan-in (NC^0) [BGKT20], and that noiseless QNC^0 can solve problems that AC^0 cannot [BKST19]. The authors also note that by combining previous results [BKST19] and [GJS21], one can also see that noisy QNC that is not necessarily 3D-local can solve problems not in AC^0. Thus, their main result advances the state-of-the-art by improving this separation for 3D-local quantum circuits, which the authors argue is necessary for practical implementations on quantum devices.*

Response: We believe this accurately summarizes our contribution and its context.

Key insights I takeaway: *Their problem construction and quantum algorithm is an elegant generalization of that used in [BGKT20] based on the magic square game. They distill the key machinery used in previous shallow circuit separations. Typical quantum circuits used in these shallow circuit separations can be stated as a bunch of Bell pairs, some classically-controlled Clifford gates, and Bell basis measurements. The authors observe that the probability of any particular measurement outcome can be written as the trace of a sequence of single-qubit Clifford and Pauli gates. Exploiting this observation, the authors strengthen the result of [BGKT20] with a simpler proof. These nice ideas are in Lemma 2.3 the proof of thm 4.1.*

Response: We thank the referee for their positive comments. Indeed, we believe that the single-qubit gate teleportation problem is arguably the simplest computational problem one can consider that leads to our complexity-theoretic separations. It is precisely this simplicity that enabled our strongest complexity-theoretic separation.

Techniques: *Before dealing with the noisy setting, they first show a separation between QNC^0 and AC^0. For this, they introduce a more general version of the 1D magic square problem used by [BGKT20]. Their QNC^0 circuit construction characterizes this definition. Proving classical hardness follows the following steps:*

- *they first show hardness for NC^0*
- *then they show that even if we restrict the problem inputs, the problem is still hard for NC^0 (when there's still n^c inputs that are still free, $c \in (0, 1]$).*
- *next, they show, using the multi-switching lemma from [BKST19] that there's a distribution over random restrictions under which with high probability an AC^0 circuit becomes an NC^0 circuit, still with n^c free inputs.*
- *AC^0 hardness follows from connecting the previous two points.*

Then, to show a separation in the presence of noise, they make use of previous works. They follow the argument used in [BGKT20] to construct a noise-robust version of the problem statement. Combined with a lemma in [GJS21] this completes their argument.

Response: We believe this summarizes the main steps. In the second step, the bound should be $\Omega(n^c)$ with $c \in (1/2, 1]$. The lower bound $c > 1/2$ is important for applying the light-cone argument.

Decision: accept *This result concretely advances our known unconditional separations between quantum and classical computation, by improving the best-known separation to one where the quantum circuit is geometrically local. This makes the quantum algorithm more feasible to actually implement on a quantum device. Their techniques generalizing the magic square game are particularly nice in that they generalize some previous works in this area.*

Response: We thank the referee for their positive evaluation and encouraging comments. We are grateful that they appreciated the significance and elegance of our approach.

Reviewer #3 (Remarks to the Author):

This paper makes a significant addition to the string of works proving unconditional separations between low-depth quantum and classical circuits. While main driver in this setting is the strength of the separation, other considerations, such as noise-resilience and locality, are of interest; this work shows a separation between noisy 3D-local QNC^0 and AC^0, a combination that was not previously known. There techniques are borrowed and adapted from prior work.

I have verified the arguments and calculations (mainly up up to section 6, inclusive) and found no reason for concern regarding the correctness of the results. I do have some concerns regarding section 7, particularly with respect to the amount of detail of proofs in that compared to previous sections.

Response: We thank the referee for their deep and thorough assessment and encouraging evaluation.

High-level/general comments

The paper is generally well-written, with detailed proofs that are easy to verify. However, my impression is that the effort and detail are not dedicated uniformly or according to the importance of each result;

Response: We are happy to receive the feedback that the presented proofs are easily verifiable. We also agree with the statement that the level of detail is not uniform. However, we disagree with the referee's view that this non-uniformity does not match the importance of the corresponding results. In fact, we have spent considerable efforts detailing derivations and providing intuition for those parts of our work which are novel. In contrast, we have kept the discussion of preexisting techniques and their application self-contained but brief. We comment on our weighting of the importance/novelty and how it differs from the referee's view below.

more to the point, the separations shown in Sections 1-6 were previously known (albeit the new results extend to the noisy 3D case, which is not the case for prior separations against AC^0) and take 37 pages, while arguably the main contribution, Section 7, is much less formal – indeed, most of the section reads like a technical overview where the formal content is concentrated on pages 46-49.

Response: In fact, we see the relative importance of Sections 1-6 and Section 7 differently (under the old numbering): Sections 1-6 are our main technical contribution, whereas Section 7 is a sort of brief corollary (immediate consequence of our results and existing work):

(I) The separation shown in Sections 1-6 is new for 1D-local QNC^0 circuits. Such a separation between AC^0 and QNC^0 was previously only known for 2D-local circuits [BKST'19]. We consider the identification of a natural problem (that of gate teleportation) amenable to a 1D-local circuit, together with a quantum advantage proof to be our main technical advance: it required a refined analysis of a suitable version of the magic square game, a new average-case analysis of the restricted problem includ-

ing light-cone argument for circuits with biased number of inputs and outputs, and a non-trivial application of the switching lemma with block restrictions.

Prior work on QNC^0 (Ref. [BGKT '20]) only established a quantum advantage against NC^0, and it was unclear whether a quantum advantage against AC^0 can even been shown in $1D$.

(II) The fault-tolerance techniques discussed in Section 7 were developed in Ref. [BGKT'20]. They are described in very general terms there, and show how to transform a 1D-local classically controlled shallow Clifford circuit into a $3D$-local fault-tolerant shallow circuit, while preserving computational hardness. More generally, it is easy to see that more generally, the construction of [BGKT'20] can be used to transform any local circuit in d dimensions into a fault-tolerant local circuit in $d+2$ dimensions. Indeed, this transformation was applied in [GJS'21] to obtain a fault-tolerant quantum advantage proposal with a local quantum circuit in 4D. As we discuss in Section 2. A, such a circuit involves non-local gates when embedded in 3D, hence is not immediately physically relevant.

The main result established in Section 7 is made possible by the advance (I), giving a $1D$-local quantum advantage proposal. The applied techniques are those of [BGKT'20], which we summarize in Section 7 to the extent necessary to understand and verify the construction. While important in establishing our main result, we consider this to be the more straightforward part of our arguments.

Changes: We emphasized the $1D$-local quantum advantage against AC^0 in both the main text, as well as in the Supplementary Information.

Reviewer's comment: *The section would greatly benefit from a formal treatment at the same level as the previous ones, e.g., by stating the relevant definitions in definition environments, clearly stating the results derived from previous work and omitting what does not directly apply. (More details follow.)*

Response: As previously mentioned, Section 7 (under the old numbering) indeed primarily discusses existing techniques. We do believe we have given a sufficient amount of detail (basically restating what is defined and discussed in similar detail in the original reference [BGKT'20]). At the same time, it is not clear to us what the referee means by "omitting what does not directly apply" – we discuss the reduction of [BGKT'20] which is used in its entirety here.

The proof of our main result in Sections 6.4 and 6.5 (previously Sections 7.4 and 7.5) is indeed a formal, and fully rigorous application of the techniques outlined in the previous sections.

Lower-level/detailed comments

Reviewer's comment: *Section 4.1: the main goal seems to be to extend the authors' earlier result [9] to the average case – which is most relevant not for its own sake, but because it is*

amenable to the AC^0 hardness proved later on. It would be useful to make the connection both to [9] and to the future sections explicitly: e.g., what exactly does Corollary 4.2 yield that is new compared to [9]? The key techniques/tools look very similar to those of [9] (namely, Pauli conjugation and light-cone arguments), so it is not immediately clear what is new. In particular, the proof of Theorem 4.1 is quite long and seems like a simple extension of prior work; if so, it could be moved to an appendix. (And if not, the novelty should be made explicit.)

Response: It is indeed the case a quantum advantage against NC^0-circuit as shown in Corollary 3.2 (new numbering; previously Corollary 4.2) was previously already known, including in the average-case setting (see e.g., in Ref. [BGKT'20]). Also, the computational problem considered here has previously been studied in [9], where a worst-case hardness result was shown.

The new average-case hardness result established by Corollary 3.2 (new numbering; previously Corollary 4.2) is much stronger because of the average-case property, and because of the particular problem considered: Average-case hardness is shown for a randomly chosen n-tuple of inputs. In contrast, the problem considered in [BGKT'20], average-case hardness is only shown for a uniform distribution over a particular subset of instances. Similarly, the arguments used in the worst-case analysis of [9] center around the consideration of a particular subset of instances (related to computing the XOR-function). In other words, in [BGKT'20] and [9], the hardness is partly derived from a promise on the input instance (it cannot be arbitrary/uniform over all instances).

Such promises are typically incompatible with the technique of random restriction, as it cannot guaranteed that the surviving restricted instances still satisfy the promise (i.e., the restriction can break the promise).

In contrast, the result established in Corollary 3.2 (new numbering; previously Corollary 4.2) makes no assumptions about the input instance (it is uniform). Establishing this result does involve some standard techniques as pointed out by the referee: Light cone arguments are essentially the only available tool here. However, a few innovations are necessary to establish this result:

First, we use a modification of the magic square game to argue about the (in)existence of certain pairs of functions (F, G) (see Lemma 1.3), giving a versatile algebraic formulation of a consequence of the magic square game In contrast, [BGKT'20] essentially uses a bound on the winning probability of an extended magic square game (i.e., classical Bell value) in place of this, whereas [9] relies on a non-contextuality property of the observables in the magic square game.

Second, we then use commutation relations between Paulis and Cliffords to argue that the existence of an NC^0-circuit with large success probability implies the existence of a pair of functions (F, G) with the relevant property. (In contrast, [BGKT'20] shows the existence of a classical strategy for violating a Bell inequality, and [9] argues that such a circuit implies a limited-depth circuit computing parity.)

In summary, though there are similarities to existing work, the details of the corresponding reductions are somewhat different. In particular, Corollary 3.2 (new numbering; previously Corollary 4.2) does not simply follow by augmenting the argument of [9] with additional detail; rather, it follows a different logic. The only essential overlap is that it

concerns the same computational problem.

Changes: We have added the following explanation before Corollary 3.2 (new numbering; Theorem 3.1 was previously Theorem 4.1, Corollary 3.2 was previously Corollary 4.2, Section 4 was previously Section 5, and Ref. [8] was previously Ref. [9]):

> "In more detail, the work [8] considers a particular subset of instances and shows that a circuit succeeding on this subset implies a constant-depth circuit computing parity, a contradiction. For Theorem 3.1 and Corollary 3.2, we consider the uniform distribution over all instances (i.e., n-tuples of Cliffords) instead. This is essential to be able to apply random restriction techniques (see Section 4), as random restrictions are generally not compatible with specific constraints on the set of considered instances."

Reviewer's comment: *Lemma 5.2: The last inequality is the only part where the assumption $\delta < 1/2$ is needed; it is worth pointing out.*

Response: There is a typo in the comment: it should be $\delta > 1/2$.

Changes: We have made the justification of the conclusion more explicit by adding the explanation

> "since $\delta > 1/2$ by assumption."

Reviewer's comment: *Section 5.2: the issue of "compatibility" of random restrictions to problems whose domain size is not a power of 2 is technically important, but in my view distracts from the main message of the paper; a reasonable alternative is to treat it accordingly and move proofs to an appendix, only keeping the formal versions of statements of the sort "representing Cliffords by bit strings makes no material difference" in the body. Moreover, it's not entirely clear why the proof of Lemma 5.3 makes use of random encodings: can't one choose the images of the $32^n - 24^n$ "leftover" strings so that the fraction of $(Enc(x), F \circ Enc(x))$ is close to the fraction of $(C, F(C))$? It seems like this lemma can have a much shorter proof. Note, also, that this proposed simplification and the current proof both assume at least one C is such that $(C, F(C))$ is not in R (otherwise $q = 1$ and the last inequality of the lemma fails to hold).*

Response: We thank the referee for this suggestion. We agree that these technical details can distract from the main message of the paper, and this material is now part of the Supplementary Information.

Reviewer's comment: *Section 6.2: properties (i), (ii) and (iii) of the distribution P should be stated either in a definition environment (which is probably best, since it's referred to elsewhere) or in the statement of Theorem 7.2.*

Response: We believe there is a typo here and that the comment should refer to Theorem 6.2 (now Theorem 5.2).

Changes: We have included properties (i), (ii), and (iii) of the distribution P in the statement of Theorem 6.2 (now Theorem 5.2 in the Supplementary Information) and provided only an informal explanation before the statement of the theorem.

Theorem 7.2:

Response: As in the previous case, the following comments relate not to Theorem 7.2 but to Theorem 6.2 (now Theorem 5.2 in the Supplementary Information).

Reviewer's comment: *Only q and t are chosen parameters but the wording suggests p_* is also "chosen". Moreover, the expression for p_* does not take the constant factor of Lemma 6.1 into account.*

Changes: We have revised the wording and propagated the O-notation from p_* throughout the proof.

Reviewer's comment: *The notation for "NC^0 of depth $20d$" is undefined; one could either explicitly define an event or define notation for the set "NC^0 of depth $20d$".*

Changes: We have added the following explanation:

> Here, by "NC^0 of depth $20d$", we mean the class of classical circuits built from fan-in 2 AND and OR gates and NOT gates, with depth at most $20d$.

We have also added the following clarification to condition (ii) of Theorem 5.2 (formerly Theorem 6.2) and to the statement of the multi-switching lemma, Lemma 5.1 (formerly Lemma 6.1):

> "(built from fan-in 2 AND and OR gates and NOT gates)"

Reviewer's comment: *$\mathcal{E}(\rho)$ seems like an odd choice of notation: $\mathcal{E}$ is a set of encodings, and ρ is a random restriction; these symbols are associated to other objects, while $\mathcal{E}(\rho)$ seems to not relate to either in the way the notation suggests. It would be best to choose another, previously unused, symbol.*

Changes: We replaced the overloaded symbol $\mathcal{E}$ with $\mathcal{T}$.

Reviewer's comment: *The second paragraph of step 2 in the definition of P mentions η as "an additional" restriction, but it is not "additional". But more importantly, the case where the set is empty is the low-probability "bad" event; it seems much more explicit to say "do nothing" or "choose an arbitrary restriction" rather than choosing a random one (which suggests there is something interesting happening in this event).*

Changes: We have changed the formulation from:

> "If the set $\mathcal{E}(\rho)$ is empty, choose an additional restriction η of the remaining free bits uniformly at random, among all such restrictions."

to ($\mathcal{E}$ was replaced by $\mathcal{T}$):

> "If the set $\mathcal{T}(\rho)$ is empty, set $\eta(x) = *$ for all $x \in \mathbb{Z}_{N(\rho)}$."

Reviewer's comment: *Figure 10: η and ρ are swapped, and the box "Implementable in NC^0" doesn't quite communicate what I think was intended: that C and C restricted by ρ may not be implementable, but C restricted by the concatenation of ρ and η (and τ) is (with high probability).*

Changes: We have corrected the figure and added the following explanation to its caption:

> "the circuit $\mathcal{C}$ restricted by ρ and subsequently by η is with high probability implementable by an NC^0 circuit and so is $\mathcal{C}$ restricted by block restriction $\xi = \tau \restriction \eta \restriction \rho$."

Reviewer's comment: *Section 7.1 and 7.2: these read like technical overviews; I would strongly suggest separating formal definitions (e.g., local stochastic noise, transversality, single-shot, etc) from discussions (e.g., the sentences starting with "By the CSS-nature..." or the discussion of the subspace to which a measurement outcome x belongs). Generally, here and in the remainder of Section 7 I found it difficult to differentiate formal definitions and results from prior work from high-level discussions.*

Changes: We have separated the definitions of p-local stochastic noise in Section 6.1 (formerly Section 7.1) and of transversal Clifford gates, single-shot state preparation, and single-shot readout in Section 6.2 (formerly Section 7.2).

Section 7.3:

Reviewer's comment: *The sentence "It is possible..." until the end of the paragraph is contradictory: it states that single-shot state preparation is "not needed" and "key to making the circuit local".*

Changes: We have changed the wording from:

> "It is possible to obtain a single-shot state preparation procedure for the logical state $|\overline{0}\rangle$, i.e., Condition 2, from this construction, but we will not need this here."

to:

> "(We note that it is also possible to obtain a single-shot state preparation procedure for the logical state $|\overline{0}\rangle$, i.e., Condition 2, from this construction.)"

Reviewer's comment: *Quite a few terms/values are undefined: "interaction graph", the faces of the prism (I assume they are the triangular faces, but this isn't immediate), "tesselation by a square lattice", "linear size of the prism", the size L, etc. This section seems like it*

would greatly benefit from crisp definitions and prior results (in particular, omitting reference to terms/defs that are not immediately relevant for the results that follow). Labeling Figure 11 with some relevant quantities would also help.

Changes:

1. We have emphasized the definition of the "interaction graph". We have changed:

   > "Locality of the circuit is defined in terms of a corresponding interaction graph (lattice): Qubits are associated with vertices of the graph, and a two-qubit gate is a nearest-neighbor gate if it acts on two qubits associated with adjacent vertices. We say that a circuit is $3D$-local if it consists of single-qubit and nearest-neighbor gates, and if the graph is embedded in $\mathbb{R}^3$ ..."

   to:

   > "We define an interaction graph associated with a quantum circuit (composed of single- and two-qubit gates) as a graph whose vertices are associated with qubits and whose edges are associated with two-qubit gates between them. The locality of the circuit is defined in terms of the corresponding interaction graph: we say that a circuit is $3D$-local if this graph admits an embedding in $\mathbb{R}^3$ ..."

2. We have included the word "triangular" to describe the faces. We have changed:

   > "are located on the "left" and "right" faces of a wedge (prism)"

   to:

   > "are located on the "left" and "right" triangular faces of a wedge (prism)"

3. We have changed:

   > "Each of these two faces is tesselated by a square lattice and has m qubits sitting on a sublattice."

   to:

   > "Each of these two triangular faces form a square sublattice and has m vertices associated with m qubits."

4. We have annotated the linear size L in Figure 11 and changed the caption of Figure 11 from:

   > "Geometric arrangement of qubits in the definition of the Single-shot Bell-state preparation procedure."

   to:

"Geometric arrangement of qubits, forming a prism, in the definition of the single-shot Bell-state preparation procedure. The linear size L of the prism is indicated."

Reviewer's comment: *r_{in} and r_{out} are not defined; they could be referenced in Figure 12, and it could also refer back to quantities in Figure 11. The figure also suggests that there's a gap between wedges, which I imagine is not the case (or the embedding would be homeomorphic to a sphere).*

Changes: We have annotated r_{in} and r_{out} in Figure 12 and added the following note to the caption:

> "The inner radius r_{in} and outer radius r_{out} of the Colosseum are indicated. The gaps between the prisms (see Figure 11) are for illustration purposes only; neighboring triangular faces are connected in the interaction graph, as illustrated in Figure 3 of the main paper."

Minor comments/typos

Reviewer's comment: *Page 1: The sentence "Subexponential..." seems disconnected, and doesn't seem relevant enough to appear in the abstract.*

Changes: We have modified the following part:

> "Such an advantage against unbounded fan-in classical circuits was previously only known in the noise-free case or without locality constraints. We overcome these limitations, proposing a quantum advantage demonstration amenable to experimental realizations. Subexponential circuit-complexity lower bounds have traditionally been referred to as exponential. We use the term colossal since our fault-tolerant 3D architecture resembles a certain Roman monument."

to:

> "Such an advantage against unbounded fan-in classical circuits has traditionally been referred to as exponential, and was previously only known in the noise-free case or when ignoring locality constraints. By overcoming these limitations, we are thus proposing the strongest unconditional, fault-tolerant quantum-advantage demonstration to date."

Page 3:

Reviewer's comment: *"subexponential" usually denotes an upper bound, so the current use is a bit confusing.*

Changes: We have removed

> “(in fact subexponential)”

from the informal version of the theorem.

Reviewer’s comment: *noise strength* $\rightarrow$ *noise rate?*

Response: Noise strength is a parameter of local stochastic noise, while noise rate of i.i.d. noise.

Changes: We have corrected this terminology in Single-shot state preparation and Single-shot Bell-state preparation conditions (formerly Condition 2 and 2’).

Reviewer’s comment: *Page 12: can use qedhere to align with the expression at the end of Lemma 2.3*

Changes: We have added qedhere to the align environment.

Reviewer’s comment: *Page 13: the parenthetical in Figure 13 "(i.e., this ... gate.)" is worded oddly; perhaps "they constitute classically controlled Clifford gates"?*

Changes: We have removed the parenthetical.

Reviewer’s comment: *Page 18: A number of* C_j *and* C_k *are swapped in items (i) and (ii) (after "This implies the following)*

Changes: We have implemented the correction.

Reviewer’s comment: *Page 23:* $|V_I| \leq \rightarrow |V_I| \geq$*, and can use qedhere too*

Changes: We have implemented the correction.

Reviewer’s comment: *Page 25: Expectation is equal to probability, not just lower bounded by it*

Changes: We have implemented the correction.

Reviewer’s comment: *Page 26:* $\mathbb{Z}^n \rightarrow \mathbb{Z}_n$ *(end of 1st paragraph of Section 5.3)*

Changes: We have implemented the correction.

Reviewer’s comment: *Page 29:* $*^{\mathsf{block}}$ *is undefined; perhaps use* $*^5$*, or a placeholder symbol other than* $*$*?*

Changes: We have added the following explanation at the first occurrence of $*^{\mathsf{block}}$:

> “Here $*^{\mathsf{block}}$ is a new symbol indicating that the whole block of 5 bits is not fixed.”

Page 30:

Reviewer's comment: *computing output → computing the output*

Changes: We have implemented the correction.

Reviewer's comment: log *is used to mean* ln*; either convention is fine, but since there are powers of 2 and powers of e in the paper, best to state what it is*

Changes: We have replaced the log by ln where logarithm of base e was used.

Reviewer's comment: *the inputs Clifford → the input Clifford*

Changes: We have implemented the correction.

Reviewer's comment: *Page 31: The denominator of (32) needs parentheses around $d-1$ in the exponent of n in the denominator*

Changes: We have implemented the correction.

Reviewer's comment: *with high probability → with probability $1-o(1)$? (Maybe best to define earlier on what "whp" means throughout the paper)*

Changes: We have harmonized the use of "with high probability" throughout the paper. We have changed the formulation:

> "In this section, we show a lower bound on the circuit depth of any classical circuit with bounded fan-in gates solving $R_{U_n^{\mathsf{Telep}}}$ with high probability on average."

to:

> "In this section, we show an average-case lower bound on the circuit depth of any bounded fan-in classical circuit solving $R_{U_n^{\mathsf{Telep}}}$ for a uniformly random input."

We have added an explanation to the first occurrence of "with high probability", i.e., we changed:

> "With high probability over the choice of $\xi^{\mathsf{block}} \sim P$, we have:"

to:

> "with high probability, i.e., with probability $1-o(1)$, over the choice of $\xi^{\mathsf{block}} \sim P$, we have:"

(Here, the lowercase "w" in "with" is due to additional changes.)

We have changed the formulation:

> "The definition of this problem and Theorem 7.1 immediately imply that even a noisy implementation of U^{ext} provides a solution to the problem $\mathcal{R}_U$ with high probability, for any input."

to (Theorem 7.1 in the previous version is now Theorem 6.1):

> "The definition of this problem and Theorem 6.1 immediately imply that even a noisy implementation of U^{ext} provides a solution to the problem $\mathcal{R}_U$ with probability greater than $1-\mu$, for any input."

Reviewer's comment: *all block restriction → all block restrictions*

Changes: We have implemented the correction.

Page 34:

Reviewer's comment: *(37) should have a strict inequality inside the probability*

Changes: We have explicit the bound leading to the desired probability.

Reviewer's comment: *Since by → By*

Changes: We have implemented the correction.

Reviewer's comment: *Page 35 and 36: Stating "depth-d AC^0 circuit" in Theorem 6.3 and Corollary 6.4 simplifies the expression for size(C_n)*

Changes: We have implemented the change.

Reviewer's comment: *Page 37: Corollary 6.5 does not need the definitions in English ("of constant ... quantum circuits" can be omitted). The corollary would also benefit from a discussion of what, if anything, is novel: is the 1D separation against AC^0 already new?*

Response: We prefer to explicit the definitions in Corollary 5.5. (formerly Corollary 6.5.).

Changes: We have emphasized separation with $1D$-local QNC^0 circuits in Corollary 5.5 (formerly Corollary 6.5) and added an explanation to emphasized the novelty:

> "In contrast to the previous work [3], where a similar result was achieved with ideal $2D$-local constant depth quantum circuits, i.e., ($2D$-local) $QNC^0 \not\subseteq AC^0$, Corollary 5.5 establishes average case quantum advantage already with ideal $1D$-local constant depth quantum circuits. This stronger starting point is crucial to obtain the main result of this paper — a quantum advantage with noisy constant depth $3D$-local quantum circuits against AC^0."

Page 39:

Reviewer's comment: *what is known about the constants c', c''? Larger than 0? Less than 1? (Likewise for Condition 2' in Page 42.)*

Changes: We have added $c', c'' > 0$ to both. (Condition 2 is now renamed to Single-shot state preparation and Condition 2' to Single-shot Bell-state preparation)

Reviewer's comment: $q_{\mathrm{th}} \to q$

Response: The notation q_{th} is correct here.

Changes: We have added missing $q < q_{\mathrm{thres}}$. The notation q_{th} has been harmonized to q_{thres}.

Reviewer's comment: $\Psi \in (\mathbb{C}^2 \to |\Psi\rangle \in (\mathbb{C}^2$

Changes: We have implemented the correction.

Reviewer's comment: *Page 43: extend circuit → extended circuit*

Changes: We have implemented the correction.

Reviewer's comment: *Page 44: subset of* $\{0,1\}^n$. → *subset of* $\{0,1\}^n$*).*

Changes: We have implemented the correction.

Page 47:

Reviewer's comment: *size D of circuit is undefined*

Changes: We have corrected this omission, included additional details, and fixed some typos in the proof of this theorem. Concretely, we have changed:

> "By relying on the standard stabilizer formalism and working in a gate-by-gate fashion, the result $P \mapsto CPC^\dagger$ of propagating a single Pauli operator P through a Clifford circuit C composed of 1- and 2-qubit gates can be computed by a depth-1 classical circuit of size D whose gates have fan-in at most 4. Recalling the definition of the logical circuit $\overline{C}_b$ in terms of transversal Clifford gates, we conclude that for any Pauli operator $P \in \mathsf{Pauli}(n \cdot m)$, the function $b \mapsto \overline{C}_b P \overline{C}_b^\dagger$ can be computed by a circuit of depth $\mathsf{depth}(U)$ and fan-in 4."

to:

> "By relying on the standard stabilizer formalism and working in a gate-by-gate fashion, the result $P \mapsto CPC^\dagger$ of propagating a single Pauli operator P through a Clifford circuit C composed of 1- and 2-qubit gates of depth D can be computed by a classical circuit of depth $O(D)$ and size $O(D)$ whose gates have fan-in at most 4. Recalling the definition of the logical circuit $\overline{C}_b$ in terms of transversal Clifford gates, we conclude that for any Pauli operator $P \in \mathsf{Pauli}(n \cdot m)$, the function $b \mapsto \overline{C}_b P \overline{C}_b^\dagger$ can be computed by a circuit of depth $O(\mathsf{depth}(U))$, size $O(n \cdot m)$ and fan-in 4."

Reviewer's comment: m_{aux}-*bits* $\to$ m_{aux}-*bit*

Changes: We have implemented the correction.

Reviewer's comment: *case, both* $\to$ *case,*

Changes: We have implemented the correction.

Reviewer's comment: *Page 48:* $O(\exp(polylog(n))) \to \exp(polylog(n))$

Changes: We have implemented the correction.

Response: Once again, we thank all three reviewers for their constructive comments.
